# Astrocytic autophagy plasticity modulates Aβ clearance and cognitive function in Alzheimer’s disease

**DOI:** 10.1186/s13024-024-00740-w

**Published:** 2024-07-23

**Authors:** Suhyun Kim, Heejung Chun, Yunha Kim, Yeyun Kim, Uiyeol Park, Jiyeon Chu, Mridula Bhalla, Seung-Hye Choi, Ali Yousefian-Jazi, Sojung Kim, Seung Jae Hyeon, Seungchan Kim, Yeonseo Kim, Yeon Ha Ju, Seung Eun Lee, Hyunbeom Lee, Kyungeun Lee, Soo-Jin Oh, Eun Mi Hwang, Junghee Lee, C. Justin Lee, Hoon Ryu

**Affiliations:** 1 K-Laboratory, Center for Brain Disorders, Brain Science Institute, Korea Institute of Science and Technology (KIST), Seoul, 02792 Republic of Korea; 2 College of Pharmacy, Yonsei-SL Bigen Institute (YSLI), Yonsei University, Incheon, 21983 Republic of Korea; 3 Department of Integrated Biomedical and Life Science, Graduate School, Korea University, Seoul, 02841 Republic of Korea; 4 Deaprtment of Medicine, Hanyang University Medical School, Seoul, 04763 Republic of Korea; 5 Center for Cognition and Sociality, Institute for Basic Science (IBS), Daejeon, 34126 Republic of Korea; 6 IBS School, University of Science and Technology (UST), Daejeon, 34113 Republic of Korea; 7 Severance Biomedical Science Institute, Graduate School of Medical Science, Brain Korea 21 Project, Yonsei University College of Medicine, Seoul, 03722 Republic of Korea; 8 Center for Advanced Biomolecular Recognition, Korea Institute of Science and Technology, Seoul, 02792 Republic of Korea; 9 Research Animal Resource Center, Korea Institute of Science and Technology (KIST), Seoul, 02792 Republic of Korea; 10 Advanced Analysis and Data Center, Korea Institute of Science and Technology, Seoul, 02792 Republic of Korea; 11 Center for Brain Function, Brain Science Institute, Korea Institute of Science and Technology (KIST), Seoul, 02792 Republic of Korea; 12 Department of Neurology, Boston University Alzheimer’s Disease Research Center, Boston University Chobanian & Avedisian School of Medicine, Boston, MA 02118 USA; 13 VA Boston Healthcare System, Boston, MA 02130 USA; 14 Department of Converging Science and Technology, KHU-KIST, Kyung Hee University, Seoul, 02447 Republic of Korea

**Keywords:** Alzheimer’s disease, Astrocytes, Amyloid beta (Aβ), Autophagy, Mitochondria, Aβ clearance

## Abstract

**Background:**

Astrocytes, one of the most resilient cells in the brain, transform into reactive astrocytes in response to toxic proteins such as amyloid beta (Aβ) in Alzheimer’s disease (AD). However, reactive astrocyte-mediated non-cell autonomous neuropathological mechanism is not fully understood yet. We aimed our study to find out whether Aβ-induced proteotoxic stress affects the expression of autophagy genes and the modulation of autophagic flux in astrocytes, and if yes, how Aβ-induced autophagy-associated genes are involved Aβ clearance in astrocytes of animal model of AD.

**Methods:**

Whole RNA sequencing (RNA-seq) was performed to detect gene expression patterns in Aβ-treated human astrocytes in a time-dependent manner. To verify the role of astrocytic autophagy in an AD mouse model, we developed AAVs expressing shRNAs for *MAP1LC3B/LC3B (LC3B)* and *Sequestosome1* (*SQSTM1)* based on AAV-R-CREon vector, which is a Cre recombinase-dependent gene-silencing system. Also, the effect of astrocyte-specific overexpression of LC3B on the neuropathology in AD (APP/PS1) mice was determined. Neuropathological alterations of AD mice with astrocytic autophagy dysfunction were observed by confocal microscopy and transmission electron microscope (TEM). Behavioral changes of mice were examined through novel object recognition test (NOR) and novel object place recognition test (NOPR).

**Results:**

Here, we show that astrocytes, unlike neurons, undergo plastic changes in autophagic processes to remove Aβ. Aβ transiently induces expression of *LC3B* gene and turns on a prolonged transcription of *SQSTM1* gene. The Aβ-induced astrocytic autophagy accelerates urea cycle and putrescine degradation pathway. Pharmacological inhibition of autophagy exacerbates mitochondrial dysfunction and oxidative stress in astrocytes. Astrocyte-specific knockdown of *LC3B* and *SQSTM1* significantly increases Aβ plaque formation and GFAP-positive astrocytes in APP/PS1 mice, along with a significant reduction of neuronal marker and cognitive function. In contrast, astrocyte-specific overexpression of LC3B reduced Aβ aggregates in the brain of APP/PS1 mice. An increase of LC3B and SQSTM1 protein is found in astrocytes of the hippocampus in AD patients.

**Conclusions:**

Taken together, our data indicates that Aβ-induced astrocytic autophagic plasticity is an important cellular event to modulate Aβ clearance and maintain cognitive function in AD mice.

**Supplementary Information:**

The online version contains supplementary material available at 10.1186/s13024-024-00740-w.

Alzheimer’s disease (AD) is a progressive neurodegenerative disorder characterized by excessive deposition of Aβ, gliosis, and neurodegeneration in the brain. Reactive astrocytes are detected in the early phases of AD even before neurodegeneration, in which astrocytes are triggered by toxic protein aggregates such as Aβ [1, 2]. The reactive astrocytes dynamically change in terms of gene expression and metabolic profiles as well as of functional properties according to the states of reactivity and disease progression [2–7]. In animal models of AD and in vitro, the detrimental mechanisms of reactive astrocytes related to excessive monoamine oxidase-B (MAO-B)/Gamma-aminobutyric acid (GABA)/H_2_O_2_ pathway have been extensively reported [6–8]. The Aβ-induced reactive astrocytes release inhibitory gliotransmitter, GABA, resulting in neuronal inhibition through MAO-B-mediated putrescine degradation pathway [7]. More importantly, this catabolic process leads to an over-production of toxic H_2_O_2_, resulting in the appearance of iNOS-positive severe reactive astrocytes and the induction of neurodegeneration [6].

## Background

In addition to the detrimental side, the beneficial side of reactive astrocytes has been also reported in AD [2–4]. Aβ is preferentially engulfed and degraded by astrocytes [9, 10], accompanying with metabolic alterations [11]. In our recent study, astrocytes are shown to degrade Aβ into toxic ammonia and detoxify ammonia into less-toxic metabolites such as urea and putrescine by turning-on the urea cycle [12]. Thus, the urea cycle serves as a beneficial metabolic pathway in reactive astrocytes. However, the excessive production of putrescine becomes the source of toxic materials by turning-on the putrescine degradation pathway, eventually leading to the production of toxic byproducts such as MAO-B-mediated GABA/H_2_O_2_/ammonia. This identification of the astrocytic urea cycle led us to postulate that there exists an upstream mechanism of the urea cycle that mediates the degradation of Aβ. Our recent study suggested that, compared to other brain cell types, astrocytes showed strong resilience against toxins due to the presence of an active autophagic process [6]. In addition, we found that autophagy is critical for Aβ-induced GABA production and release from reactive astrocytes [12]. These lines of evidence implicate that the autophagy pathway could be an upstream mechanism of the astrocytic urea cycle in AD.

Autophagy is a catabolic process that is involved in the degradation of cellular components such as damaged organelles or abnormal protein aggregates and occurs spontaneously to maintain cell homeostasis [13]. It is a multistep process that is characterized by *de novo* formation and sequestration of double-membrane vesicles, autophagosome, and lysosome-fused vesicles, autolysosome. Three major types of autophagy have been described in mammalian cells, including macroautophagy, microautophagy and chaperon-mediated autophagy [13, 14]. Depending on its substrates, macroautophagy can be divided into aggrephagy or mitophagy [15, 16]. It has been reported that autophagy dysfunction is linked to various brain diseases such as AD, amyotrophic lateral sclerosis (ALS), Huntington’s disease (HD), Parkinson’s disease (PD) and stroke [17–21]. In the brain, astrocytes display active metabolism with more oxidative and glycolytic properties than other brain cell types and respond to the disease-associated molecules. Astrocytes preferentially uptake Aβ protofibrils and produce large endosomes, but incomplete degradation of Aβ protofibrils eventually leads to astrocytic lysosomal dysfunction and neuronal cell death [10]. Moreover, astrocytic autophagy function is known to be critical for the regeneration of mitochondrial networks and viability of astrocytes [22]. Although it has been reported that the basal autophagy machinery is indispensable for astrocytic function, the exact molecular mechanism of astrocytic autophagy in Aβ catabolism and AD pathogenesis has not been fully elucidated yet.

In this study, we comprehensively tested the hypothesis that the astrocytic autophagy is critical for clearance of Aβ, maintenance of mitochondrial activity, reactivity of astrocytes and survival of neurons. In this regard, we performed our study to find out whether Aβ-induced proteotoxic stress affects the expression of autophagy genes and the modulation of autophagic flux in astrocytes, and if yes, how Aβ-induced autophagy-associated genes are involved Aβ clearance in astrocytes of animal model of AD. Using cultured astrocytes, AD mouse models and samples from human AD patients, we delineate and establish the detailed molecular mechanisms and their time-courses of astrocytic autophagy, which is closely associated with the AD pathologies such as Aβ plaque formation, reactive astrogliosis, and neurodegeneration.

## Methods

### Animals

APPswe/PSEN1dE9 (APP/PS1) mice of B6C3 hybrid background (RRID: MMRRC_034829-JAX, from stock number 004462) and 5XFAD mice were originated from Jackson Laboratory (USA). APP/PS1 maintained as hemizygotes by crossing transgenic mice to B6C3 F1 mice. Genotypes were determined by PCR using the following primers – APP/PS1f − 5’ AAT AGA GAA CGG CAG GAG CA 3’; APP/PS1r − 5’ GCC ATG AGG GCA CTA ATC AT 3’. All mice were maintained under 12:12-h light-dark cycle (lights on at 8:00 AM) and has ad libitum access to food and water. Animal care and handling were performed according to the directives of the Animal Care and Use Committee and institutional guidelines of KIST (Seoul 02792, South Korea). Immunohistochemistry and behavioral tests were performed on virus-injected mice, for which both sexes of 10- to 13-month-old transgenic mice and wild-type littermates were used.

### Chemicals

We used several autophagy inhibitors: chloroquine (CQ) (Sigma, C6628, USA), a lysosome fusion inhibitor; epoxysuccinyl-leucylamide(3-methyl-butane ethyl ester)(E64D) (Sigma, E8640, USA), a cysteine protease inhibitor; Pepstatin A (P) (Sigma, 77,170, USA), an aspartate protease inhibitor; 3-methyladenine (3-MA), a PI3-kinase inhibitor (Sigma, 189,490, USA). KDS2010, an ROS scavenger and reversible MAO-B inhibitor was obtained from Institute of Basic Science (IBS, Daejeon, South Korea).

### Cell culture

Primary cortical astrocytes were prepared from postnatal day (P) P0–P3 C57BL/6 mice as described [23]. The cerebral cortex was dissected free of adherent meninges, minced and dissociated into single-cell suspension by trituration. Dissociated cells were plated onto plates coated with 0.1 mg/ml poly-D-lysine (Sigma, USA). Cells were grown in DMEM (Invitrogen, USA) supplemented with 25 mM of glucose, 10% heat-inactivated horse serum, 10% heat-inactivated FBS, 2 mM of glutamine and 1,000 Uml^− 1^ penicillin-streptomycin. Cultures were maintained at 37 °C in a humidified 5% CO_2_ incubator. On the third day of culture, cells were vigorously washed with repeated pipetting and the medium was replaced to get rid of debris and other floating cell types. Human astrocyte cell line was purchased from Applied Biological Materials Inc. company (Immortalized Human Astrocytes, fetal-SV40; Cat. No. T0280; Canada). The human astrocyte cell line was maintained in DMEM supplemented with 25 mM of glucose, 10% heat-inactivated FBS, 2 mM of glutamine and 1,000 Uml^− 1^ penicillin-streptomycin. The passages of astrocytes between 10 and 20 were used for in vitro experiments.

### Preparation of oligomeric form of Aβ42

Amyloid-beta 1–42 (Aβ; DAEFRHDSGYEVHHQKLVFFAEDVGSNKGAIIGLMVGGVVIA; Abcam, USA) was prepared as previously described [24]. Aβ was dissolved in dimethyl sulfoxide (DMSO) at 10 mM and further diluted to 1 mM in PBS and incubated at 37 °C for 1 week and stored at -80 °C for further use as an oligomeric form of Aβ (Aβ oligomer). The monomeric form of Aβ (Aβ monomer) was prepared using same sequence without oligomerization process.

### Immunocytochemistry

For immunostaining in cultured cells, the cells were rinsed with PBS twice and fixed with 4% PFA for 10 min at room temperature. Cells were then washed with PBS twice and permeabilized through incubation in 0.1% Triton X-100 in PBS for 15 min at room temperature. Nonspecific binding was blocked through incubation in 3% bovine serum albumin (Sigma, USA), normal goat serum (Abcam, USA) or normal donkey serum (Abcam, USA) in PBS with 0.1% Triton X-100 for 2 h at room temperature. Afterwards, cells were incubated with the following antibodies for 24 h: anti-LC3B antibody (1:500, Novus bio, USA); anti-SQSTM1 antibody (1:500, Abcam, USA), anti-glial fibrillary acidic protein (GFAP; 1:500, Abcam, USA), anti-calcium-binding protein B (S100B; 1:500, Synaptic systems, Germany), anti-microtubule associated protein 2 (MAP2; 1:500, Abcam, USA) and anti-Neuronal nuclear antigen (NeuN; 1:400, Abcam, USA). After secondary antibody reaction, samples were washed 3 times with PBS and examined under a confocal microscope.

### Immunohistochemistry

Mice were deeply anesthetized with 2% avertin (20 mg/ml, 20 µl/mouse weight (g); intraperitoneal injection) and perfused with 0.9% saline followed by ice-cold 4% paraformaldehyde (PFA). Excised brains were postfixed overnight at 4 °C in 4% PFA and dehydrated in 15% and 30% sucrose for 48 h. Coronal hippocampal sections were cut at 30 μm in a cryostat and stored in storage solution at 4 °C. Sections from storage solution were washed in PBS and incubated for 1 h in a blocking solution (0.3% Triton X-100, 2% normal donkey serum in 0.1 M of PBS). Primary antibodies in blocking solution were immunostained on a shaker at 4 °C overnight. After washing in PBS 3 times, sections were incubated with corresponding fluorescent secondary antibodies for 1 h at room temperature and then washed with PBS at 3 times. The nuclei were counter stained with 4′,6-diamidino-2-phenylindole (DAPI) was adding in solution (1:5,000; Abcam, USA) during the second washing step. Finally, sections were mounted with fluorescent mounting medium (Dako, USA) and dried at room temperature. A series of fluorescent images were obtained with a Nikon A1 confocal microscope (Nikon, Japan) with 26 μm Z stack images in 2 μm steps were processed for Sholl analysis using the NIS-Elements software (*Ver.* 4.5, Nikon, Japan) and ImageJ software (*Ver.* 1.52s, NIH, USA). Primary antibodies were diluted to the following amounts: anti-LC3B (1:500, Abcam, USA), anti-SQSTM1 (1:200, MBL, USA), anti-AT8 (1:200, Abcam, USA), anti-GFAP (1:500, Abcam, USA), and anti-NeuN (1:400, Millipore, USA). Secondary antibodies were diluted 1:500 in the blocking solution for 2 h at room temperature.

### Human brain samples

Neuropathological examination of postmortem brain samples from normal subjects, (NeuroPathologically and Clinically Diagnosed AD)-MCI (NPCAD) and severe AD (SAD) patients was determined using procedures previously established by the Boston University Alzheimer’s Disease Center (BUADC) [25]. Next of kin provided informed consent for participation and brain donation. Institutional review board approval for ethical permission was obtained through the BUADC center. This study was reviewed by the Institutional Review Board of the Boston University School of Medicine and was approved for exemption because it only included tissues collected from post-mortem subjects not classified as human subjects. The study was performed in accordance with institutional regulatory guidelines and principles of human subject protection in the Declaration of Helsinki. The sample information is listed in Supplementary Table S3.

### Double chromogenic staining for human brain tissues

#### First staining

Paraffin-embedded human postmortem brain tissues were sectioned in a coronal plane at 10 μm. BLOXALL^®^ Blocking solution (SP-600, Vector Laboratories, USA) was used to block endogenous alkaline phosphatase. Hippocampal tissue sections were blocked with 2.5% normal horse serum (S-2000, Vector Laboratories, USA) for 1 h and then incubated with GFAP antibody (1:400 dilution) (AB5541, Millipore, USA) for 24 h. After washing three times with PBS, tissue slides were processed with Vector ABC Kit (PK-4000, Vector Laboratories, USA). The GFAP immunoreactive signals were developed with DAB chromogen (D7304, Thermo Fisher Scientific, USA).

#### Second staining

Tissue slides stained with GFAP were incubated with LC3B antibody (1:200 dilution) (ab192890, Abcam, USA) for 24 h. After reaction with secondary antibodies, sections were incubated with ImmPRESS-AP anti-rabbit IgG (Aalkaline phosphatase) polymer detection reagent (MP-5401, Vector Laboratories, USA) for 2 h at room temperature. A Vector Blue alkaline phosphatase substrate kit (SK-5300, Vector Laboratories, USA) was used to develop LC3B signals. Double-stained tissue slides were gradually processed back to Histo-clear (HS-200, National Diagnostics, USA) through an increasing ethanol gradient [70%, 80%, 90%, 95%, and 100% (1 time)] and subsequently mounted. The chromogenic signals of GFAP (brown) and LC3B (blue) were examined under a light microscopy (BX63, Olympus, Japan) equipped with high definition (1920 × 1200 pixel) digital camera (DP74) (Olympus, Japan).

### 3D reconstruction of microscopic image

Images were analyzed using a Spinning Disk Confocal microscope (IX2-DSU, Olympus, Japan) that has z-stack modulation stage. The 40x objective images were acquired z-stack in a 0.25 μm steps and deconvolved using Cell Sense software (Olympus, Japan). The deconvolution images were applied to the process of 3D reconstruction with IMARIS software (*Ver. 13*, Oxford Instrument, UK). The clipping plane mode was used to clearly visualize the localization of Aβ plaques and astrocytes.

### Illumina Hiseq library preparation and sequencing

Sample libraries were prepared by Ultra RNA Library Prep Kit (#E7530, NEBNEXT, USA), Multiplex Oligos for Illumina (#E7335, NEBNEXT, USA) and poly(A) mRNA Magnetic Isolation Module (#E74900, NEBNEXT, USA) according to the manufacturer’s instructions. Full details of the library preparation and sequencing protocol are provided on the website (https://international.neb.com/products/e7530-nebnext-ultra-rna-library-prep-kit-for-illumina#Product%20Information). The Agilent 2100 Bioanalyzer (Agilent Technologies, USA) and the associated High Sensitivity DNA kit (Agilent Technologies, USA) were used to determine quality and concentration of the libraries. Sample libraries for sequencing were prepared by the HiSeq Reagent Kit Preparation Guide (Illumina, USA) as described previously [26]. Briefly, the combined sample library was diluted to 2 nM, denatured with 0.2 N fresh NaOH, diluted to 20 pM by addition of Illumina HT1 buffer. The library (600 µl) was loaded with read 1, read 2 and index sequencing primers on a 150-cycle (2 × 75 paired ends) reagent cartridge (HiSeq Reagent kit, Illumina, USA), and run on a HiSeq NEXT generation high-throughput sequencer (Illumina, USA). After the 2 × 75 bp Illumina HiSeq paired-end sequencing run, the data were base-called and reads with the same barcode were collected and assigned to a sample on the instrument, which generated Illumina FASTQ files.

### Quantitative real time-PCR (qPCR)

Total RNA was isolated from cells and brain tissues using a commercial extraction system (Qiagen, USA). 1 ﻿µg total RNA has been used for cDNA preparation with iScript cDNA Synthesis Kit (Bio-Rad, USA) according to manufacturer’s protocols. cDNA from each sample was amplified by real-time PCR using iQ SYBR Green Supermix (Bio-Rad, USA). RNA quantities were normalized using glyceraldehyde-3-phosphate dehydrogenase *(GAPDH)* mRNA. PCR cycling conditions were denaturation for 3 min at 95°C; then 40 cycles of amplification for 15 sec at 95°C, 15 sec at 60°C, 20 sec at 70°C; followed with 30 sec at 72°C. For melt curve data collection has been used 33 cycles, 6 sec each, with the temperature increased from 60°C to 92°C (increase set point temperature after cycle 2 by 1°C). The PCR primer for *LC3B* were as following: forward, 5’-ATC CCG GTG ATA ATA GAA CG -3’ and reverse, 5’-GAA GAA GGC CTG ATT AGC AT -3’; The PCR primer for *SQSTM1* were as following: forward, 5’-ATG ACT GGA CCC ATC TGT CT-3’ and reverse, 5’-TCA TCA GAG AAG CCC ATG GA-3’, The PCR primer for Beclin1 *(BECN1)* were as following: forward, 5’-AGG TAC CGA CTT GTT CCC TA-3’ and reverse, 5’-TCC ATC CTG TAC GGA AGA CA-3’. The PCR primer for *GAPDH* were as following: forward, 5’- GAA ATC CCA TCA CCA TCT TCC-3’ and reverse, 5’- GAG GCT GTT GTC ATA CTT CTC-3’.

### Western blot analysis

Western blot analyses were performed as described previously. The transferred blots were incubated with the following primary antibodies at 4 °C for 24 h: anti-LC3B (1:2000, Abcam, USA); anti-SQSTM1(1:2000, Abcam, USA); anti-ACTB/β-actin (1:2000, ACTB; Abcam, USA) and anti-TUBB3/tubulin (1:2000, TUBB3; Abcam, USA). After washing 3 times with Tris-buffered saline with 0.05% Tween 20), the blots were incubated with the appropriate secondary antibodies conjugated to horseradish peroxidase (HRP) by anti-rabbit HRP (Amersham Pharmacia, USA) at room temperature for 2 h. Then, the blots were developed by Immobilon Western ECL solution (Merck Millipore, USA) and immunoreactive bands were visualized using an Image Station 4000MM (#745,280; Kodak, Japan). ACTB or TUBB3 was used as the loading control.

### Cell viability assay and cell death assay

Cell viability of primary astrocyte cells were assessed using a 3-(4,5-dimethylthiazol-2-yl)-5-(3-carboxy-methoxyphenyl)-2-(4-sulfophenyl)-2 H-tetazolium inner salt (MTS) assay, which is based on a tetrazolium compound, MTS, and an electron acceptor agent, phenazine methosulfate (PMS; Promega, USA). Briefly, cells were seeded into a 96-well plate at a density of 3 × 10^4^ cells per well in 200 µl of medium, stabilized for growth, and then treated with various concentrations of Aβ oligomers and autophagy inhibitors in 100 µl of medium. After 24 h of incubation at 37 °C, 20 µl of MTS/PMS mixture solution was added to the culture medium, and cells were further incubated for 3 ∼#x2009;4 hr at 37 °C. Finally, absorption readings were performed at 490 nm using a spectrophotometry. Astrocyte cells were seeded in a 96-well plate at 1 × 10^4^ cells per well. After overnight, the Aβ oligomers were added to the wells with 1 µg/ml. After 24 h, inhibitors added to the wells with E/P. After indicated times, the cellular viability was determined by Cell Titer-Glo reagent (#G7572, Promega, USA). Dose-response curve was fitted using Graphpad prism 8.0 software (GraphPad Software, USA). All assays were performed in triplicate, and standard deviation (SD) was determined from three independent experiments. Apoptosis and necrosis of astrocyte cells was quantified by Annexin V and EthD-III based quantification assay kit (#30,065, Biotium, USA). Astrocyte cells were treated with Aβ oligomers and autophagy inhibitors for 24 h, followed by incubation with a staining solution at room temperature for 15 min. Live cell imaging was obtained by Image ExFluorer (Live Cell Instrument, Korea). The intensity and quantification of cell death signals were analyzed with ImageJ software (Fiji; NIH, USA).

### Live cell imaging in astrocyte culture with MitoTracker and MitoSOX

MitoTracker-green (Thermo Scientific, USA) staining was performed to measure the mitochondrial morphology in astrocyte cells as previously described [27]. Mito-Sox (Thermo Scientific, USA) staining was performed to measure the oxidative stress in astrocyte cells. MitoTracker-green (0.1 µM) and Mito-Sox (2.5 µM) staining for 30 min at 37 °C incubator prior to acquire images. Live cell imaging was obtained by Image ExFluorer (Live Cell Instrument, Korea). The intensity of mitochondria oxidative stress and mitochondria morphology were analyzed by ImageJ software (NIH, USA).

### DCF-DA assay

Intracellular ROS levels were detected using cell-permeable non-florescent probe 2′,7′-Dichlorofluorescin diacetate (#D6883, DCFDA; Sigma, USA). DCF-DA is de-esterified into its fluorescent form after action of intracellular esterases and oxidation by reactive oxygen species within the cell [28]. Primary astrocyte culture was seeded onto 48-well plates (Corning, USA) and treated with Aβ oligomer (1 µM) in the presence or absence of KDS2010 (100 nM), a ROS scavenger and reversible MAO-B inhibitor, or CQ (20 µM) for 1 day. Then, cells were washed twice with Hanks Buffered Salt Solution (#LB-003-002, HBSS; Welgene, USA) and incubated with 30 µM DCF-DA in Hanks′ Balanced Salt solution (HBSS) at room temperature for 30 min in the dark. The DCFDA was replaced with HBSS and fluorescence was measured using SpectraMax iD5 Multi-Mode Microplate Reader (Excitation 485 nm and emission 538 nm, Molecular Devices, USA).

### Amplex Red assay

Extracellular ROS levels were detected using Amplex Red reagent after collecting the media from cell culture treated with Aβ oligomer (1 µM) in the presence or absence of KDS2010 (100 nM) or CQ (20 µM) for 1 day.

### Metabolite analysis

For metabolite analysis, aspartate, ornithine, ^15^N-ornithine, arginine, ^15^N-arginine, citrulline, ^15^N-citrulline, glutamate, putrescine, ^15^N-putrescine, GABA and ^15^N-GABA were analyzed. The system used for the analyses was an Exion LC AD UPLC coupled with an MS/MS (Triple Quad 4500 System, AB Sciex LLC, Framingham, USA) using an Acquity^®^ UPLC BEH C18 column (1.7 μm, 2.1 mm x 75 mm, Waters, USA) at 50℃, controlled by Analyst 1.6.2 software (AB Sciex LP, Ontario, Canada). 70% methanol (100 µl, with internal standard d_5_-glutamine at a final concentration of 1 µM) was added to the astrocyte sample pellets and vortexed for 30 s. Cells were lysed by three consecutive freeze/thaw cycles using liquid nitrogen, and the lysate was centrifuged for 10 min at 20,817 x g (14,000 rpm) at 4℃. The supernatant (5 µl) from each sample was used for DNA normalization. DNA concentrations were analyzed using a Nano-MD UV-Vis spectrophotometer (Scinco, Seoul, Korea). 40 µl of the supernatant from each sample was evaporated to dryness at 37℃ under a gentle stream of nitrogen. Phenylisothiocyanate (PITC) derivatization was performed by adding 50 µl of a mixture of 19:19:19:3 ethanol:water pyridine:PITC (v/v) and the mixture was vortexed for 30 s and shaken for 20 min. Then the mixture was evaporated to dryness at 37℃ under a gentle stream of nitrogen. The residue was reconstituted by adding 50 µl of the mobile phase A (0.2% formic acid in deionized water): B (0.2% formic acid in acetonitrile) = 5:5 solvent and vortexing for 30 s. The initial chromatographic conditions were 100% solvent A at a flow rate of 0.4 mL·min^− 1^. After 0.9 min at 15% B, solvent B was set to 15% over the next 4.1 min, solvent B was set to 70% over the next 5 min, solvent B was set to 100% over the next 0.5 min, and these conditions were retained for an additional 2 min. The system was then returned to the initial conditions over the next 0.5 min. The system was re-equilibrated for the next 2.5 min in the initial conditions. The total running time was 15 min. All samples were maintained at 4℃ during the analysis, and the injection volume was 5 µl. The MS analysis was performed using ESI in positive mode. The ion spray voltage and vaporizer temperature were 5.5 kV and 500℃, respectively. The curtain gas was kept at 45 psi, and the collision gas was maintained at 9 psi. The nebulizer gas was 60 psi, while the turbo gas flow rate was 70 psi. The metabolites were detected selectively using their unique multiple reaction monitoring (MRM) pairs. The following MRM mode (Q1 / Q3) was selected: arginine (m/z 310.000 / 217.000), ^15^N-arginine(m/z 311.000 / 218.000), ornithine (m/z 403.200 / 310.200), ^15^N-ornithine (m/z 404.000 / 311.200), citrulline (m/z 311.200 / 113.100), ^15^N-citrulline(m/z 313.200 / 114.100), glutamate (m/z 283.200 / 130.200), aspartate (m/z 269.200 / 116.200), putrescine (m/z 359.200 / 266.100), ^15^N-putrescine(m/z 360.200 / 267.100), GABA (m/z 238.875/ 87.103), ^15^N-GABA(m/z 239.875 / 87.103). As to monitor specific parent-to-product transitions, the standard calibration curve for each metabolite was used for absolute quantification.

### Electron microscopy of primary astrocyte culture

In order to fix human astrocytes, media was replaced with pH 7.2-4 buffered 2.5% glutaraldehyde for 1 h. And cells were collected and embedded in epon resin. Subsequently, 70 nm sections were obtained using an ultra-microtome and stained with uranyl acetate and lead citrate. Cell sections were finally analyzed using an 80 kV transmission electron microscope. To quantify the autophagic compartments, size and number were measured autophagic vesicles which were defined by dark and circular shape vesicles in cytoplasmic region [29]. For immune-gold labelling, astrocyte culture cells were fixed and incubated with anti-LC3B (NB100-2220, Novus, USA) antibody in blocking solution (0.5% Ttiton-X100 with 5% NGS in PBS) and proceed with goat anti-rabbit-gold particle antibody (G3779, 10 nm, Sigma, USA, ).

### Viral plasmids and virus production

For gene silencing in astrocytes of mouse hippocampus in vivo, we generated adeno-associated virus (AAV)-CREon shRNAs for *LC3B* (GenBank NM_026160.5) and *SQSTM1* (GenBank NM_011018.3) by applying a newly developed pAAV-R-CREon vector that turns on shRNA in a GFAP promoter (pGFAP)-driven CRE-dependent manner through AAV-pGFAP-CRE as previously described [30]. In order to generate an astrocyte-specific viral vector, we subcloned a 681 bp of human GFAP promoter, gfaABC1D, to AAV2 vector [31]. It is well established that the 681 bp gfaABC1D promoter sequence possess higher transcriptional activity in an astrocyte-specific manner similar the 2.2 kb gfa2 promoter [31]. Otherwise, to induce astrocyte-specific cell death and observe the degree of Aβ deposition in the hippocampus of APP/PS1 mice., we applied AAV-LoxP-active Caspase 3 and AAV-GFAP-Cre together [32]. LC3B shRNA sequence, 5’-GCAGCTTCCTGTTCTGGATAA-3’; SQSTM1 shRNA sequence, 5’-ACTGGACCCATCTGTCTTCAA-3’. On the other hand, to determine the gain of LC3B function in astrocytes, we inserted the cDNA of human *LC3B* (GenBank NM_022818.5) into AAV-pGFAP overexpression system. To generate high-titer AAV at concentrations ranging from 1 × 10^9^ to 10^11^ plaque-forming units per milliliter (pfu/ml), desired plasmids and pRC5, along with the pHelper plasmids, were transiently introduced into HEK293TN cells. Following a 72 h incubation, cell lysates were subjected to benzonase treatment (50 units/ml; Sigma, USA). Then, viral particles were purified and concentrated using a heparin column (GE Healthcare, Sweden) in conjunction with a 100k filtering tube (Millipore, USA). Quantitation of viral titers was measured by qPCR method.

### Stereotaxic injection

Mice were anesthetized with 2% avertin (200 mg/kg; Sigma, USA) in saline (20 µl/g; saline volume/mouse body weight) and placed in a stereotaxic frame (Stoelting Co, USA). AAV-R-CREon-LC3B or SQSTM1 shRNA and AAV-pGFAP-CRE overexpression viruses were co-injected using a stereotaxic micro-injector (Stoelting Co. USA). Control groups were injected with AAV-scramble shRNA. AAV containing solution (2 µl) was injected into the molecular layer of dentate gyrus (DG) in dorsal hippocampus; anterior-posterior (AP): -2.0 mm, medial lateral (ML): ±1.5 mm, dorsal ventral (DV): -1.85 mm to bregma using a micro-syringe pump (Micro 4, WPI, USA) with a 33-gauge needle (WPI, USA) (0.1 µl/min). Neuropathological experiments were performed at 3 weeks after injection. Mice were housed on a 12:12 h light-dark cycle and maintained at 18 ∼ 23 °C with humidity between 40 and 60% in pathogen-free facilities at Korea Institute of Science and Technology.

### Behavior tests

#### Novel object recognition (NOR) and novel object place recognition (NOPR)

NOR and NOPR tasks were performed in a white open field box (40 × 40 × 40 cm) with slight modifications from the procedures. Two types of objects were different in shape, color and texture. One of them was a yellow regular tetrahedron, made of acryl. The other one was a black and red color sphere, made of urethane. The objects were fixed to the ground of the box, not to be moved by mice. Sniffing objects was considered as the explorative action of mouse. NOR test was composed of 3 steps such as habituation, training and test, and given once per day. During the habituation step, mouse was placed in an open field box for 10 min without objects. Then, during 2 times of training period, two identical objects were presented to the mouse for 10 min. At 2 h after the last training, one of the familiar objects was replaced with a novel object and presented to the mouse for 10 min for the test. Procedures for NOPR task were similar to NOR task except that one of the objects was moved to a different location for the test. The test was video-recorded with encoding software (Ethovision XT, Noldus, USA) and the results including total number of arm entries and alternation behavior were analyzed later.

#### Quantification and statistical analysis

Cell culture preparation and mice with the required genotypes were randomly assigned to the experimental groups and treated in the same way. All quantitation analyses were done blindly. Numbers and individual dots refer to individual samples (individual cells, separate batches of cultured cells or animals) unless clarified otherwise in figure legends. N represents number of animals used for the experiment, while *n* refers to number of cells or culture batches. Data distribution was assumed to be normal but this was not formally tested. Data are presented as the mean ± SEM. For behavioral analysis, Ethovision XT (Noldus, USA) was used. For image analysis, ImageJ software (NIH, USA) and IMARIS software (Oxford instrument, UK) was used. All statistical analyses were performed using Prism v.8.4.3 (GraphPad Software, USA); an unpaired, two-tailed Student’s t-test was used to compare two groups. A one-way analysis of variance (ANOVA) with Tukey’s multiple comparisons test was used to compare more than two groups as indicated in the figure legends. A two-way ANOVA with Tukey’s multiple comparisons test was used to compare groups having two independent variables. Statistical differences were considered significant when *p* < 0.05 and the significance was set at *, *p* < 0.5 and **, *p* < 0.01, which are indicated in the figures or figure legends. No statistical methods were used to predetermine sample sizes but sample sizes are similar to those reported in previous publications. The number of experimental samples, mean, SEM and additional statistics values are listed in Supplementary Tables S1 and S2.

## Results

### Aβ induces expression of autophagy components and increases autophagy flux in astrocytes

**Fig. 1:**
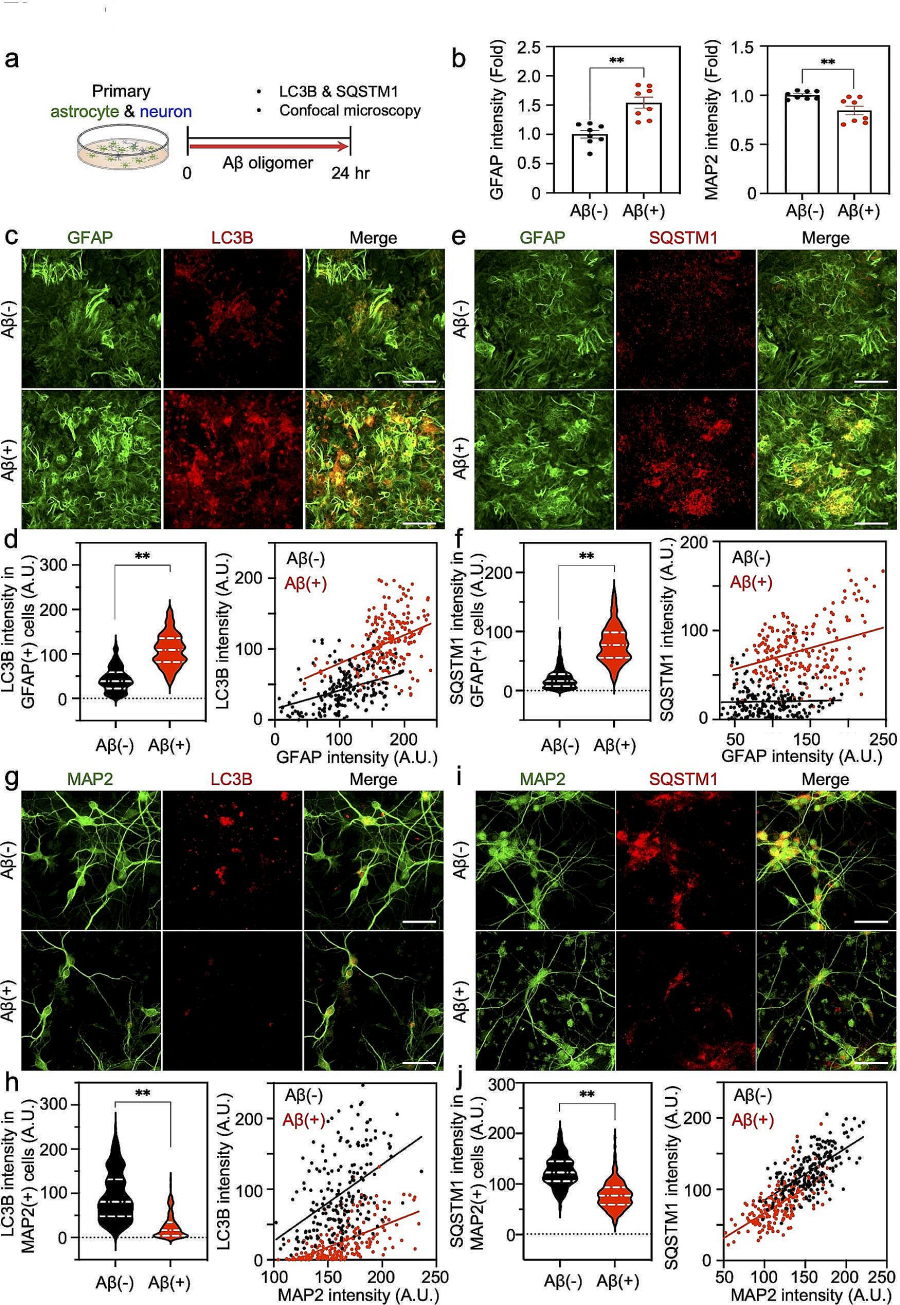
Astrocytes induce autophagy components (LC3B and SQSTM1) in response to Aβ oligomer but neurons do not. **a**, Experimental scheme for detecting autophagy components in primary mouse astrocyte and neuron culture. **b**, Quantification of GFAP or MAP2 immunoreactivity in the primary mouse astrocyte and neuron coculture system with or without Aβ oligomer. A total of 400 cell count, 50 cells/well, *n* = 8 wells. **c**, Double immunostaining with GFAP and LC3B antibodies in the primary culture system. Scale bar (white): 50 μm **d**, Quantification of LC3B immunoreactivity in GFAP-positive astrocytes and their correlation graphs with GFAP intensity. A total of 200 cell count, 50 cells/well, *n* = 4 wells. **e**, Double immunostaining with GFAP and SQSTM1 antibodies in the primary culture system. Scale bars (white): 50 μm. **f**, Quantification of SQSTM1 immunoreactivity in GFAP-positive astrocytes and their correlation graphs with GFAP intensity. A total of 200 cell count, 50 cells/well, *n* = 4 wells. **g**, Double immunostaining with MAP2 and LC3B antibodies in the primary culture system. Scale bar (white): 50 μm. **h**, Quantification of LC3B immunoreactivity in MAP2-positive regions and their correlation graphs with MAP2 intensity. A total of 200 cell count, 50 cells/well, *n* = 4 wells. **i**, Double immunostaining with MAP2 and SQSTM1 antibodies in the culture system. Scale bar: 50 μm. **j**, Quantification of SQSTM1 immunoreactivity in MAP2-positive regions and their correlation graphs with MAP2 intensity. A total of 200 cell count, 50 cells/well, *n* = 4 wells. A.U.: arbitrary unit, pixel. Significantly different at **, *p* < 0.01

To examine whether autophagy components are regulated by Aβ in astrocytes, we adopted primary mouse astrocyte-neuron cultures, treated with synthetic Aβ in vitro. We prepared Aβ oligomers and treated them to the culture medium at a concentration of 1 µM (Fig. 1a). We performed immunocytochemistry (ICC) using antibodies against astrocytic and neuronal markers, GFAP and MAP2, and found that the GFAP level was significantly increased (Fig. 1b, c, e), whereas the MAP2 level was significantly decreased in response to Aβ (Fig. 1b, g, i). This was consistent with our previous report on non-cell autonomous mechanism of severe reactive astrocytes causing neurodegeneration via H_2_O_2_ in AD [6]. We also observed that the major autophagy components, LC3B and SQSTM1, were significantly and correlatively up-regulated along with the increased GFAP levels (Fig. 1c-f), whereas those of neuron were significantly down-regulated along with the decreased MAP2 levels (Fig. 1g-j.

We also verified that Aβ oligomer treatments increased LC3B and SQSTM1 levels in S100B-positive primary astrocytes (Supplementary Fig. 1a-d). In contrast, Aβ oligomer treatments decreased LC3B and SQSTM1 levels in NeuN-positive primary neurons (Supplementary Fig. 1a-d).

**Fig. 2:**
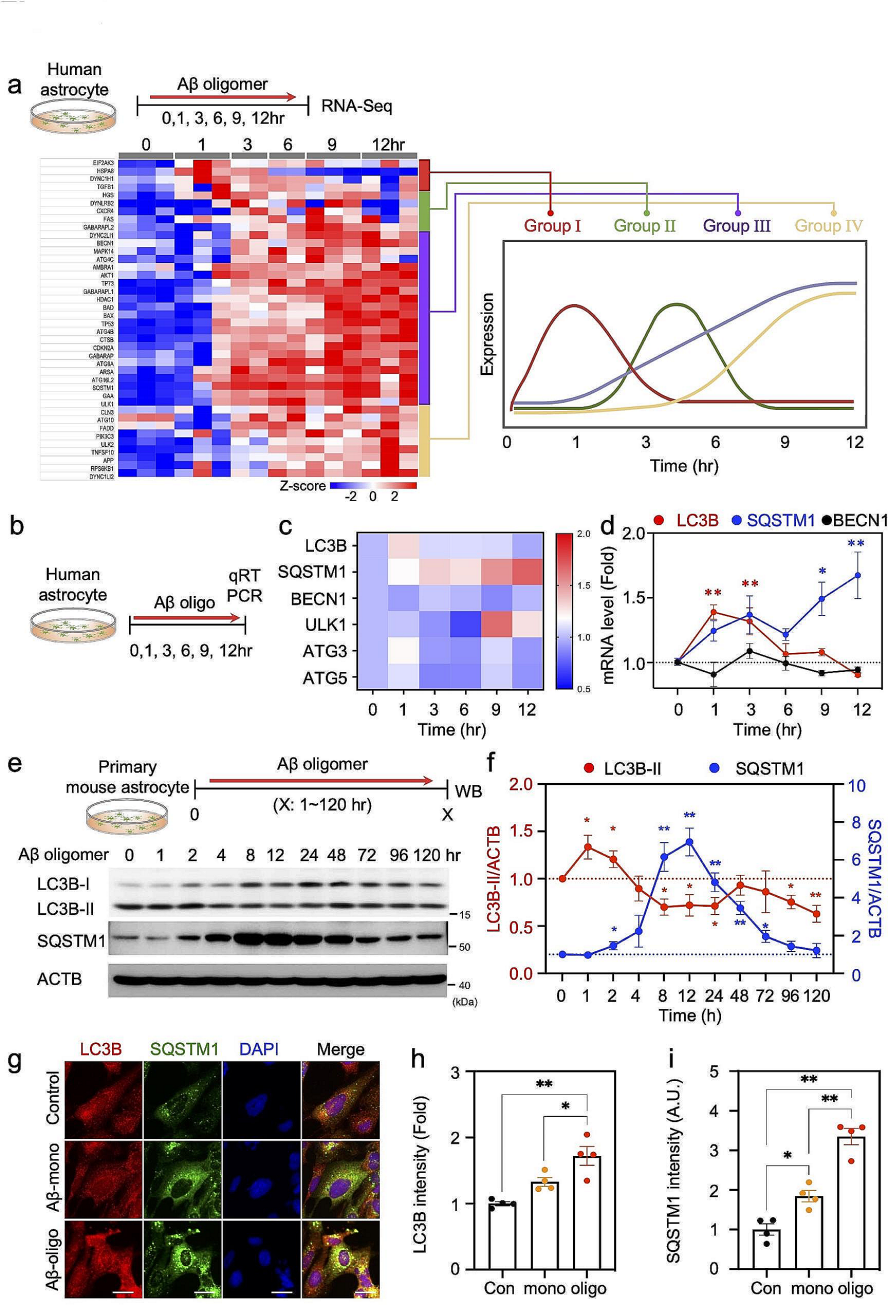
Astrocytes exhibit temporal expression of autophagy-related genes and autophagy flux in response to Aβ oligomer in a timely manner. **a**, RNA-seq analysis in human astrocyte culture at 1, 3, 6, 9 and 12 h after Aβ oligomer treatment. The altered gene expression was categorized into four groups: Group I, II, III and IV depending on the time point of peak expression. **b**, Experimental design for a time course of qRT-PCR in Aβ oligomer-treated human astrocytes. **c**, Heatmap of the expression pattern for autophagy-associated genes. **d**, qRT-PCR analysis for LC3B, SQSTM1 and BECN1 mRNA levels in response to Aβ oligomer. Line graphs present the mean ± SEM of three separate experiments. **e**, Western blot analysis data showing that levels of endogenous LC3B-II and SQSTM1 are time-dependently changed in Aβ oligomer-treated primary mouse astrocytes. **f**, Quantification of the band intensities of LC3B-II and SQSTM1 normalized by β-actin (ACTB). Line graphs present the mean ± SEM of three separate experiments. **g**, Double immunostaining with LC3B and SQSTM1 antibodies in monomeric or oligomeric Aβ-treated human astrocyte culture. Scale bars (white): 20 μm. **h & i**, Bar graphs showing the average intensity of LC3B (**h**) and SQSTM1 signals (**i**). A total of 20 cell count, 5 cells/well, *n* = 4 wells. Data are presented as mean ± SEM. Significantly different at *, *p* < 0.05; **, *p* < 0.01

Based on the above data, we hypothesized that Aβ can modulate the expression of autophagy-related genes for Aβ detoxification in astrocytes. To prove this hypothesis, we performed whole RNA-seq to detect gene expression patterns in Aβ-treated human astrocytes at 1, 3, 6, 9–12 h. We observed that autophagy-related genes show a significant increase of RNA levels upon Aβ-treatment. We categorized those genes into four groups showing temporal patterns of RNA expression in a time-dependent manner (Fig. 2a). We validated that the mRNA levels of *LC3B* and *SQSTM1* are significantly elevated after Aβ oligomer treatment, suggesting that an increase in autophagy components occurs at gene expression level in response to Aβ oligomer in astrocytes (Fig. 2b-d). Next, to determine the precise time-course of Aβ-induced astrocytic autophagy at protein level, we assessed the expression of autophagic components such as LC3B-I, LC3B-II and SQSTM1 in response to Aβ oligomer by Western blot analysis in primary mouse astrocytes (Fig. 2e, Supplementary Fig. 2a). LC3B-I, which is a cleaved form of LC3B at its C-terminus, is shown to be diffusely distributed in the cytosol. When autophagy is induced, cytosolic LC3B-I is conjugated to phosphatidylethanolamine (PE) to form LC3B-II which is moved to the autophagosomal membranes. Interestingly, the levels of SQSTM1 and LC3B-II show dynamic changes occurring within 120 h. The time of peak expression of SQSTM1 and LC3B-II is 12 h and 1 h after Aβ oligomer treatment, respectively, and the levels were returned to the normal level (Fig. 2f). Interestingly, these temporal dynamics of autophagic components were also observed in the treatment conditions of pro-inflammatory factors such as lipopolysaccharide (LPS) and tumor necrosis factor (TNF)-alpha as well as Aβ monomer (Supplementary Fig. 2b-d). Finally, we treated either Aβ monomer or oligomer and checked the accumulation of autophagic vesicles in astrocytes by immunostaining with LC3B and SQSTM1 antibodies (Fig. 2g). We observed a punctate pattern, indicating autophagosome, in the cytosol of astrocytes and found that Aβ oligomer formed more dense vesicles than Aβ monomer (Fig. 2g-i). These results indicate that the autophagic components are increased with a transient temporal dynamic in response to Aβ monomer and oligomer in human astrocytes, and these were commonly shown in a similar manner against various pro-inflammatory factors.

### Autophagy components (LC3B and SQSTM1) are elevated in the brain of AD patients

**Fig. 3:**
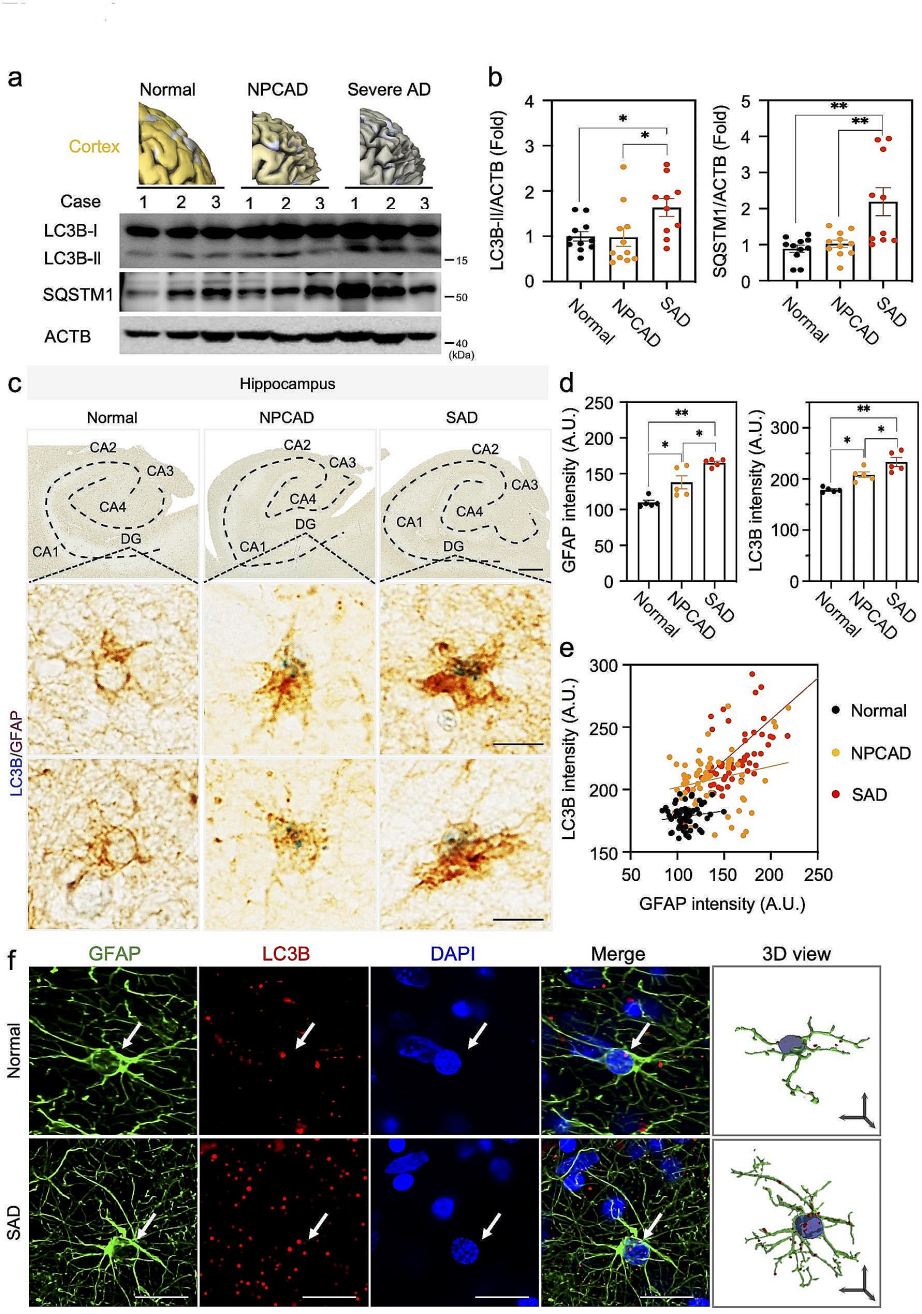
Astrocytic LC3B and SQSTM1 levels are increased in the hippocampus of AD patients. **a**, Western blot analysis for detecting the protein level of LC3B-II and SQSTM1 from the postmortem cortex of normal subject, NPCAD-MCI, and severe AD (SAD) patients. **b**, Densitometry analysis of LC3B-II and SQSTM1 protein levels form panel a. Normal, *N* = 11 cases; NPCAD, *N* = 11 cases; SAD, *N* = 10 cases. **c**, Double chromogenic immunostaining images for LC3B (blue) and GFAP (brown) in the hippocampus of normal, NPCAD, and severe AD patients. Bottom panels exhibit LC3B (blue) immunoreactivity in GFAP-positive astrocyte (brown) in the dentate gyrus (DG). Scale bars (black): top, 2 mm; middle and bottom, 10 μm. **d**, Quantification of GFAP and LC3B immunoreactivity. One dot represents an average of 12 cells (a total of 60 cells), 12 cells/case, each *N* = 5 cases for normal, NPCAD, and SAD. **e**, A correlation analysis between GFAP and LC3B intensities in the DG of normal, NPCAD, and SAD patients (derived from panel d). **f**, Representative confocal images of LC3B and GFAP immunoreactivity in the cortex of normal subject and severe AD patients. Arrow (white) indicates LC3B signal in GFAP-positive astrocyte. Scale bars: white, 20 μm; black arrows (x, y, & z in 3D view), 10 μm. Data are presented as mean ± SEM. Significantly different at *, *p* < 0.05; **, *p* < 0.01

To determine the importance of autophagy in AD, we examined whether the autophagy components change in the brain of AD patients. We measured the level of major autophagy components, LC3B and SQSTM1 by performing Western blot experiment using human brain samples from cortical regions of AD patients (Fig. 3a, b). We found the significant increase of SQSTM1 and LC3B only in severe AD, but not in the condition of NPCAD (Fig. 3a, b). Based on these results, we performed immunohistochemistry experiment for LC3B with astrocyte-specific marker, GFAP in the brain tissue of human patients with AD (Fig. 3c-e). As previously reported, the morphology of astrocytes was shown as hypertrophied in the case of AD compared to healthy group [6], indicating the presence of the increase of GFAP-positive astrocytes. As a result, we observed the increased level of LC3B in the hippocampal regions of human AD patients, compared to the case of healthy subjects (Fig. 3c-f, Supplementary Fig. 3). Interestingly, the level of LC3B signals were correlated with the level of GFAP intensity in astrocytes of AD patients (Fig. 3e), implicating that the autophagic components are accumulated depending on the reactivity of reactive astrocytes in AD brain. These alterations of protein expression implicate that the astrocytic autophagy pathway might play an important role in AD.

### Induced autophagy function is essential for astrocyte survival in response to Aβ

**Fig. 4:**
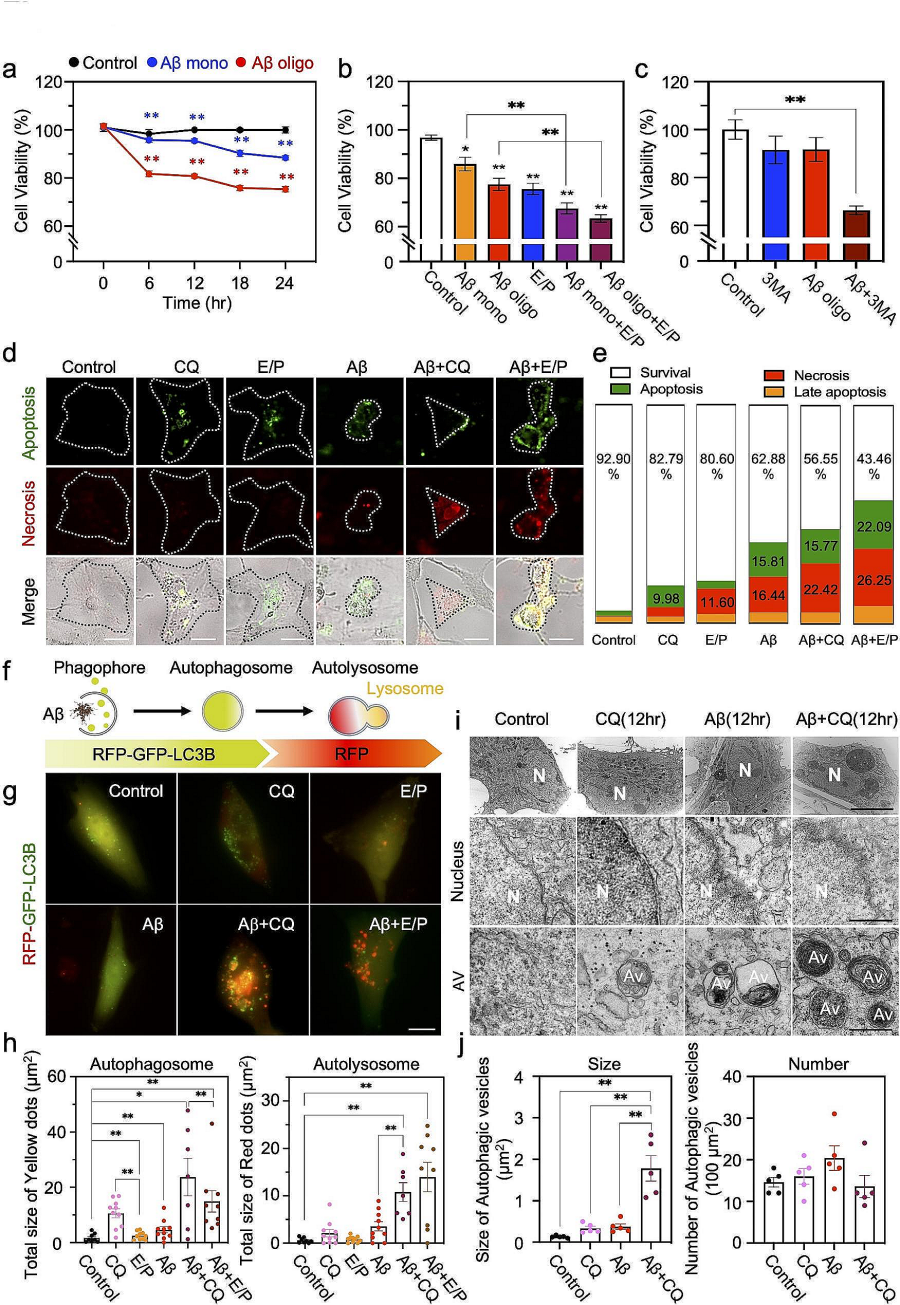
Blockage of autophagy pathway decreases survival of astrocytes in response to Aβ oligomer. **a**, Aβ monomer or oligomer induces astrocytic cell death. Treatment conditions in human astrocytes: Aβ monomer, 1 µM; Aβ oligomer, 1 µM. Cell viability was measured by MTT assay. **b**, Inhibition of autophagy function exacerbates cell death in Aβ monomer or oligomer-treated human astrocytes. Treatment conditions: Aβ monomer or oligomer, 1 µM; E64D/Pepstatin A (E/P), acidic protease inhibitors, 10 µg/ml; time, 24 h. Bar graphs represent mean ± SEM from three separate experiments. **c**, 3MA, a PI3-kinase and autophagosome formation inhibitor, exacerbates cell death of Aβ oligomer-treated primary mouse astrocytes. Treatment conditions: Aβ oligomer, 1 µM; 3MA, 1 mM; time, 24 h. **d**, Representative images of astrocyte morphology, apoptotic cell death signals (green), and necrosis cell death signals (red) with or without autophagy inhibitors [E/P or chloroquine (CQ)] in human astrocytes under Aβ oligomer treatment. Treatment conditions: Aβ oligomer, 1 µM; E64D, 10 µg/ml; Pepstatin A, 10 µg/ml; CQ, 20 µM; time, 24 h. Scale bars (white): 10 μm. **e**, Ratio change of cell death patterns (apoptosis, necrosis, and late apoptosis) in astrocytes with or without autophagy inhibitors. **f**, Working mechanism of autophagy sensor, RFP-GFP-LC3B. Green dots indicate autophagosomes and red dots indicate autolysosomes. **g**, Representative images of RFP-GFP-LC3B-expressing astrocytes in the presence or absence of autophagy inhibitors (CQ or E/P) in Aβ oligomer-treated human astrocytes. Scale bars: 10 μm. **h**, Quantification of the size of autophagosome (green dots) and autolysosome (red dots). Cell count: control, *n* = 8, CQ, *n* = 10; E/P, *n* = 10; Aβ, *n* = 10; Aβ + CQ, *n* = 7; Aβ + E/P, *n* = 9. **i**, Representative transmission electron microscopy (TEM) images representing autophagosome (double membrane vesicles) and autolysosome (dark and dense vesicles) in Aβ oligomer-treated cultured astrocytes. Scale bars (black): upper, 5 μm; middle and bottom, 0.5 μm. Av, autophagy vesicles; N, nucleus. **j**, Quantification of size and number of autophagic vesicles. Data are presented as mean ± SEM. Measurement and count of ROI (100 µm^2^)/cell. Significantly different at *, *p* < 0.05; **, *p* < 0.01

Previous studies suggest that autophagy is critical for cell survival [33, 34]. Consistently, our previous report is showing that the autophagy mechanism is related with the cell viability of astrocytes against toxic material, which is DT in the case using astrocytic inducible simian diphtheria toxin receptor (iDTR) system, a newly developed model of reactive astrocytes [6]. In order to determine whether astrocyte viability is associated with the autophagy function, we designed pharmacological experiments with several autophagy inhibitors: CQ, a lysosome fusion inhibitor; E64D, a cysteine protease inhibitor; Pepstatin A (P), an aspartate protease inhibitor; 3-MA, a PI3-kinase and autophagosome formation inhibitor. When the autophagy machinery did not work properly in Aβ-treated human and mouse astrocytes through pharmacological inhibition, the viability of astrocytes was significantly reduced (Fig. 4a-c). First, we performed MTT assay to measure the NAD(P)H-dependent oxidoreductase activity of cells for assessing metabolic activity in human cultured astrocytes. We observed that not only Aβ monomer and oligomer reduce the viability of astrocyte but also E64D/Pepstatin A (E/P) significantly reduce the viability of astrocytes. Notably, we further found that co-treatment of Aβ and autophagy inhibitors accelerates the cell death of astrocytes (Fig. 4a, b). On the other hand, we observed that the co-treatment of Aβ and 3-MA consistently decreased the viability in primary mouse astrocyte culture (Fig. 4c). The patterns of cell death (apoptosis versus necrosis) were also examined through the morphological analysis of astrocytes in CQ and E/P -treated and/or Aβ-treated astrocytes accompanied with cell death markers (Fig. 4d, e, Supplementary Fig. 4a). Interestingly, both apoptotic and necrotic cell death of astrocytes were significantly elevated by autophagy inhibition in response to Aβ treatment. The alteration of astrocyte morphology was also observed in an animal model of AD by knockdown of *MAP1LC3B/LC3B* gene using LC3B shRNA CreON system, which is a Cre-dependent shRNA-expressing system along with fluorescent protein (mCherry) expression [30]. We found that the astrocyte morphology was markedly disrupted in LC3B shRNA-expressed and Aβ oligomer-treated astrocytes, accompanying with the accumulation of Aβ aggregates (Supplementary Fig. 5a-c). This result indicates that astrocytic autophagy mechanism is necessary for astrocytic survival against toxic stimulus, implicating the resiliency of astrocytes under toxic environments or disease conditions is dependent upon the autophagy function.

Autophagy process shows unique morphological alterations by forming autophagosome, which is a double-membrane sequestered vesicle, or autolysosome, which is a fusion form of autophagosome with lysosome [35–37]. These unique subcellular features can be directly seen through electron microscopy techniques [29]. Therefore, we performed transmission electron microscopy (TEM) to observe autophagosome and autolysosome in Aβ-treated primary mouse astrocytes. As a result, we could detect an increase of cytosolic double membrane vesicles, which are autophagosomes, in Aβ oligomer-treated astrocytes and the dark and dense vesicles, which are possibly autolysosomes, in Aβ oligomer-treated astrocytes (Fig. 4i, j). Additionally, we performed EM analysis and obsevred that astrocytes around the vicinity of Aβ plaques in the hippocampus of 5xFAD mouse contain increased filament structures and show autophagic vesicles (double membrane) while astrocytes in the hippocampus of WT mouse contain less filamnt strutures and did not show apparent formation of autophagic vesicles (Fig. S7). Then, we examined whether the increased autophagic machinery leads to the increased autophagic flux in astrocytes by applying CQ, which is an inhibitor of autophagosome-autolysosome conversion, or a lysosomal enzyme inhibitor, E64D (10 ﻿µg/ml) and Pepstatin A (10 ﻿µg/ml) (E/P). Firstly, we directly immunostained with LC3B or SQSTM1 antibodies to detect autophagosome and observed that Aβ-induced accumulation of LC3B or SQSTM1 was greater in CQ or E/P-treated conditions than in untreated conditions (Supplementary Fig. 6a-c). To further confirm an increase in the autophagic flux induced by Aβ, we monitored the autophagic flux with generally used fluorescence-based autophagy probe, RFP-GFP-LC3B fusion construct [38–40], which can show yellow signals in early autophagosomes and red signal in autolysosome (Fig. 4f-h, Supplementary Fig. 4b). Aβ induced an increase of both the number and the size of autophagosome (yellow puncta) and autolysosomes (red puncta) in each astrocyte and these signals were further accumulated in co-treatment of CQ or E/P (Fig. 4g-h). Additionally, we showed that the enhanced autophagic flux was also observed in the treatment of Aβ monomer as well as Aβ oligomer one through Western blot analysis (Supplementary Fig. 2). We measured the levels of LC3B or SQSTM1 from primary astrocyte culture lysates and compared the accumulated ratio of each markers by treatment of CQ or E/P between the absence and the presence of Aβ monomer in primary mouse astrocytes. As a result, these ratios were significantly increased by Aβ monomer treatment (Supplementary Fig. 6d-h). Also, we directly observed that the size of autophagic vesicles dramatically increased in the presence of CQ through TEM experiment (Fig. 4i, j). Taken together, these results indicate that Aβ, not only oligomeric form but also monomeric one, accelerates the cellular process of autophagy in astrocytes accompanying with dynamic regulation of autophagy-related proteins.

### Inhibition of autophagy prevents Aβ intoxication and exacerbates oxidative stress in Aβ-treated astrocytes

Previous studies have shown that, in astrocytes, Aβ increased GABA and H_2_O_2_ production through MAO-B-mediated putrescine degradation pathway [6, 7] and, as an upstream mechanism of putrescine production by uptake of Aβ, astrocytic urea cycle is switched-on in AD conditions [12]. In this study, the urea cycle metabolites such as aspartate, arginine, and ornithine as well as putrescine and GABA were markedly upregulated in the Aβ-treated cultured astrocytes, indicating the accelerated urea cycle [12]. Here, we postulated that autophagy pathway can acts as an upstream mechanism of urea cycle in the process of Aβ intoxication. To investigate whether the Aβ-triggered urea cycle is modulated by autophagy pathway in astrocytes, we used LC3 shRNA to inhibit autophagy pathway in Aβ-treated astrocytes and analyzed the intracellular urea cycle metabolites by liquid chromatography/mass spectrometry (LC/MS) analysis (Supplementary Fig. 8a). Notably, we observed that Aβ oligomer-induced elevation of aspartate, arginine, ornithine, putrescine, and GABA levels were significantly attenuated in LC3B-shRNA-transfected primary mouse astrocytes (Fig. 6b-g). Whereas shRNA LC3B only did not alter the level of urea cycle-associated metabolites such ornithine, arginine, and others (Fig. 6b-g). These results indicate that the Aβ-facilitated urea cycle in astrocytes is mediated by autophagy pathway.

**Fig. 5:**
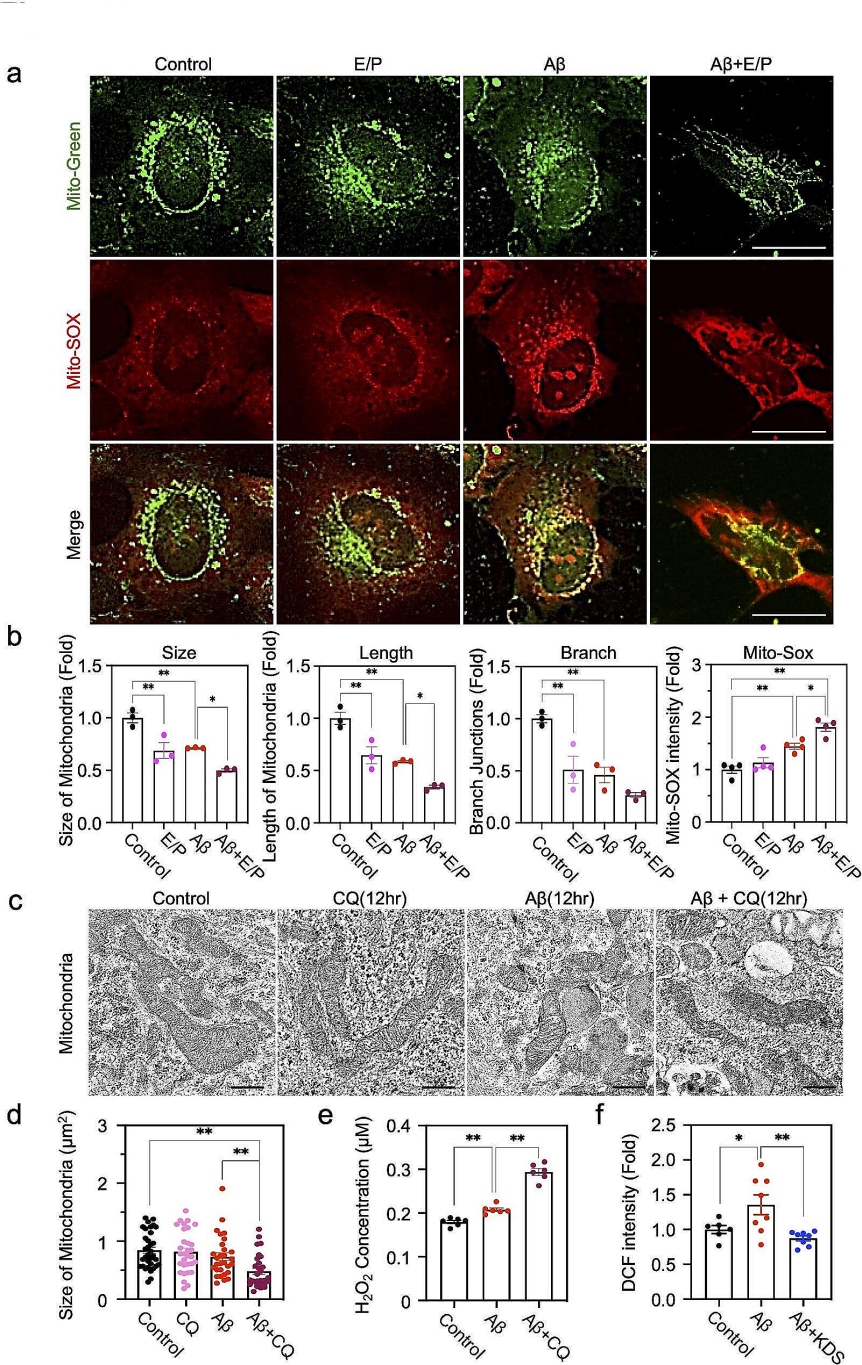
Inhibition of astrocytic autophagy function dysregulates mitochondrial membrane potential and elevates ROS in response to Aβ oligomer. **a**, Representative images of mitochondrial membrane potential (by MitoTracker staining) and mitochondrial reactive oxygen species (ROS) (by MitoSOX staining) in Aβ oligomer-treated human astrocyte culture with or without E/P. Scale bars (white): 10 μm. **b**, Quantification of MitoTracker and MitoSOX signals in Aβ oligomer-treated human astrocytes with or without E/P. A total of 20 cell counts, 5 cells/well, *n* = 4 wells. **c**, Representative TEM images presenting ultrastructural changes of mitochondria in Aβ oligomer- and/or CQ-treated astrocytes. Scale bars (black): 0.5 μm. **d**. Quantification of the mitochondria size from EM images among four groups: control, 34 mitochondria counts; CQ, 26 mitochondria counts; Aβ, 29 mitochondria counts; Aβ + CQ, 29 mitochondria counts. Measurement of mitochondria size in 100 µm^2^ of ROI. **e**, Amplex UltraRed assay for detecting H_2_O_2_ in Aβ oligomer- and/or CQ-treated astrocytes. Bar graphs represent mean ± SEM from 6 wells. **f**, DCF-DA assay for detecting ROS in Aβ oligomer-treated astrocytes with or without KDS2010 (KDS), a ROS scavenger and reversible MAO-B inhibitor. Bar graphs represent mean ± SEM from 6 wells (control), 8 wells (Aβ), and 8 wells (Aβ + KDS2010). Significantly different at *, *p* < 0.05; **, *p* < 0.01

Considering that autophagy is closely related with mitochondrial dynamics [41] and Aβ in amyloidogenic AD mouse model caused mitochondrial dysfunction, which exacerbate as Aβ plaques are formed [42], we could hypothesize that Aβ-induced autophagy of astrocytes may contribute to the modulation of mitochondrial function. Accordingly, we analyzed the RNA-seq data and found that mitochondrial genome-encoded mitochondria genes and nuclear genome-encoded mitochondria targeting genes are significantly altered in Aβ oligomer-treated human astrocytes (Supplementary Fig. 8h, i). Next, we examined mitochondrial morphological change with Mito-Tracker (green), which is a valuable tool used to observe mitochondrial morphology and activities [27, 43], in Aβ oligomer -treated human astrocytes. We found that the size and length and total branch junctions of mitochondria were significantly lower in either Aβ oligomer- or E/P-treated astrocytes than control group and these were further decreased in the condition of E/P- and Aβ oligomer-co-treatment, compared to the single treatment (Fig. 5a, b). The mitochondrial dysfunction in autophagy-depleted and Aβ oligomer-treated astrocytes was also observed in TEM image (Fig. 5c, d). Finally, we measured mitochondrial reactive oxygen species (ROS) using Mito-SOX, which a mitochondria-specific superoxide indicator, and observed that the Mito-SOX intensity was significantly increased by Aβ treatment, which was further increased in Aβ- and E/P- co-treated condition (Fig. 5a, b). Consistently, we observed an increase of H_2_O_2_ level from culture media 1 day after treating Aβ through Amplex Red assay with further increase in autophagy-inhibited condition (Fig. 5e). Detection of ROS using DCF-DA assay was conducted in Aβ oligomer-treated astrocytes with or without KDS2010, a ROS scavenger and reversible MAO-B inhibitor [6] (Fig. 5f). These results indicate that Aβ oligomer-induced astrocytic autophagy is closely associated with the intact functions of mitochondrial and ROS homeostasis.

### Astrocyte-specific knockdown of autophagy components exacerbates Aβ plaques formation and impairs cognitive function in APP/PS1 mice

**Fig. 6:**
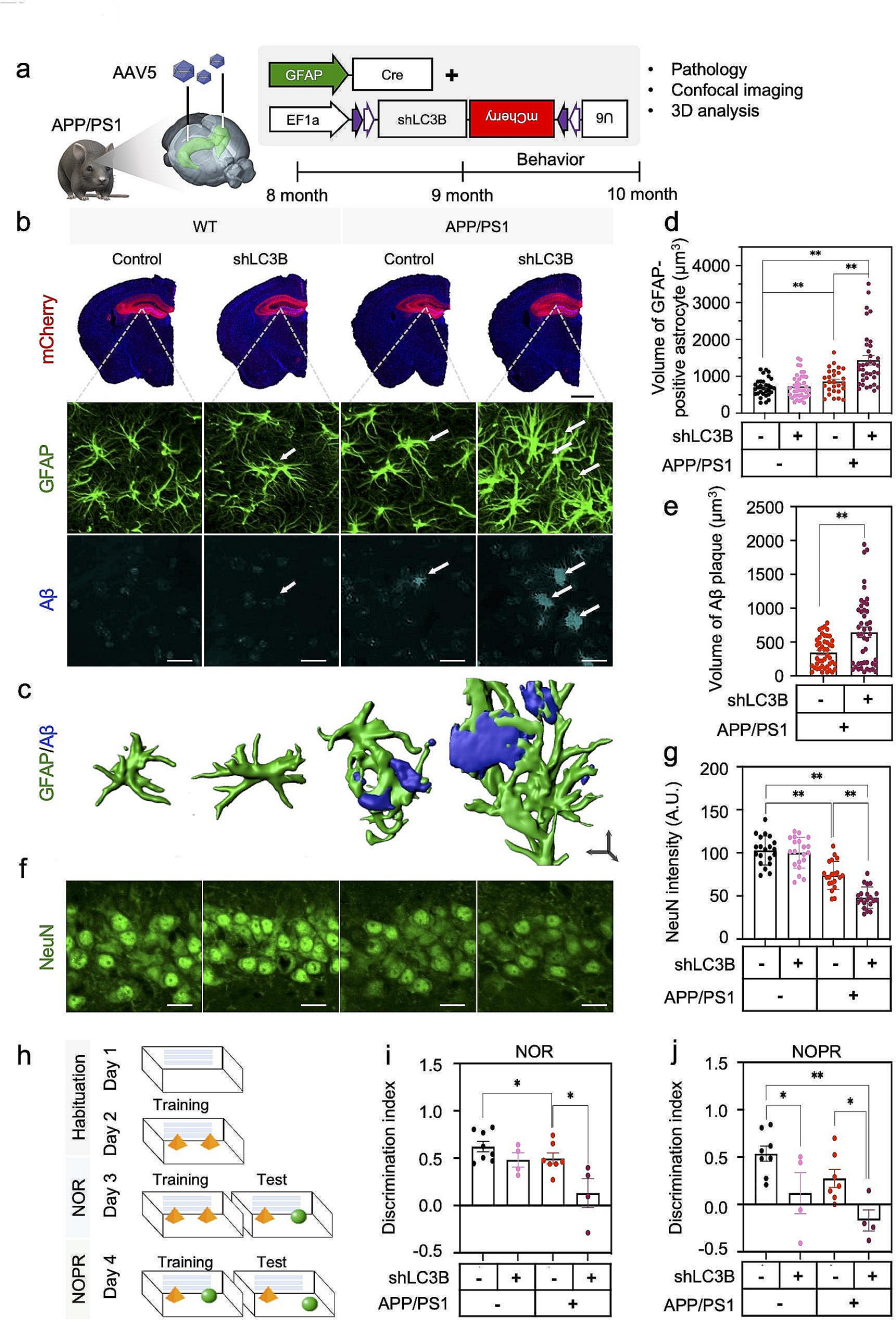
Astrocyte-specific knockdown of MAP1LC3B/LC3B escalates Aβ plaque formation, GFAP-positive astrocytes, and cognitive impairment in APP/PS1 mice. **a**, Experimental scheme for genetic inhibition of astrocytic autophagy in the hippocampus of WT and APP/PS1 mice. **b**, Double immunostaining for GFAP and Aβ plaque in four groups of mice: Group 1, WT mice with AAV-EF1a-DIO-Control shRNA + AAV-pGFAP-Cre; Group 2, WT mice with AAV- EF1a-DIO-LC3B shRNA + AAV-pGFAP-Cre; Group 3, APP/PS1 mice with AAV-EF1a-DIO-Control shRNA + AAV-pGFAP-Cre; Group 4, APP/PS1 mice with AAV-EF1a-DIO-LC3B shRNA + AAV-pGFAP-Cre. Scale bars: black, 1 mm (upper); white, 20 μm (bottom). **c**, 3D rendering images (by IMARIS) of GFAP-positive astrocytes (green) and Aβ plaques (blue). Scale bar: black arrows (x, y, & z), 10 μm (3D). **d**, Quantification for the volume of GFAP-positive astrocytes in four group of mice: WT control, a total of 31 cell counts from *N* = 4 mice; WT + LC3B shRNA, 40 cell counts from *N* = 4 mice; APP/PS1 control, 27 cell counts from *N* = 4 mice; APP/PS1 + LC3B shRNA, 36 cell counts from *N* = 4 mice. **e**, Quantification for the volume of Aβ plaques in APP/PS1 control and APP/PS1 + LC3B shRNA mice. A total of 45 plaque counts from *N* = 4 mice in each group. **f**, Immunofluorescence images for NeuN-positive neurons in four groups of mice as described in panel b. Scale bars (white): 20 μm. **g**, Quantification of NeuN immunoreactivity in four groups of mice: a total of 20 ROIs measurement from *N* = 4 mice in each group. **h**, Timeline and work flow for behavioral tests (NOR, novel object recognition test; NOPR, novel object place recognition test) in four group of mice: WT control, *N* = 8 mice; WT + LC3B shRNA, *N* = 4 mice; APP/PS1 control, *N* = 7 mice; APP/PS1 + LC3B shRNA, *N* = 4 mice. **i & j**, Bar graph showing discrimination index in NOR (**i**) and NOPR (**j**) behavior tests in four groups of mice. Data are presented as mean ± SEM. Significantly different at *, *p* < 0.05; **, *p* < 0.01

To investigate the role of astrocytic autophagy in the pathology of AD, we developed an efficient shRNA for *LC3B* gene and generated an AAV expressing LC3B shRNA based on CREon shRNA system, which is a Cre recombinase-dependent gene-silencing system (Supplementary Fig. 9a-h). We injected this virus with AAV-GFAP-Cre virus into the hippocampus of WT or APP/PS1 mice at 8-month-old (Fig. 6a) and genetically knockdown the expression of LC3B specifically in hippocampal astrocytes of WT and APP/PS1 mice. 8 weeks after virus injection, we examined the number of Aβ plaques in the brain of APP/PS1 mice by immunostaining with GFAP and Aβ antibodies or thioflavin-S staining. We observed that the size of Aβ plaques was significantly increased in the brain of APP/PS1 mice expressing LC3B shRNA, compared to control virus group (Fig. 6b, c,e, Supplementary Fig. 12a). This result indicates that astrocytic autophagy activation has a critical function in clearing up Aβ in AD. Interestingly, the astrocytes in LC3B shRNA-expressing APP/PS1 became more hypertrophied in their morphology than control group (Fig. 6b-d, Supplementary Fig. 11a-c), even though not further ramified, implicating the aggravated reactivity of astrocytes most likely due to excessive H_2_O_2_-production. Knock down of *LC3B* increased the number of GFAP-positive astrocytes and Aβ plaques accompanying with the reduction of intensity and number of NeuN signals in the hippocampus of APP/PS1 mice (Fig. 6f, g, Supplementary Fig. 11d, e). These results indicate that astrocytic autophagy mechanism has a critical function in preventing the exacerbation of AD pathology in amyloidogenic mouse model. On the other hand, to examine whether astrocyte toxicity alone is sufficient to induce Aβ pathology in vivo, we induced astrocyte-specific cell death in the hippocampus of WT and APP/PS1 mice by transducing active caspase-3 (using AAV-LoxP-active Caspase 3 and AAV-pGFAP-Cre) (Supplementary Fig. 10a-c). As a result, we found that both the number and size of Aβ aggregates were significantly increased in the hippocampus of APP/PS1 mice with astrocyte-specific active caspase-3 overexpression (Supplementary Fig. 10d, e). This data indicates that astrocyte-specific cell death exacerbates the aggregation of Aβ in AD mice.

**Fig. 7:**
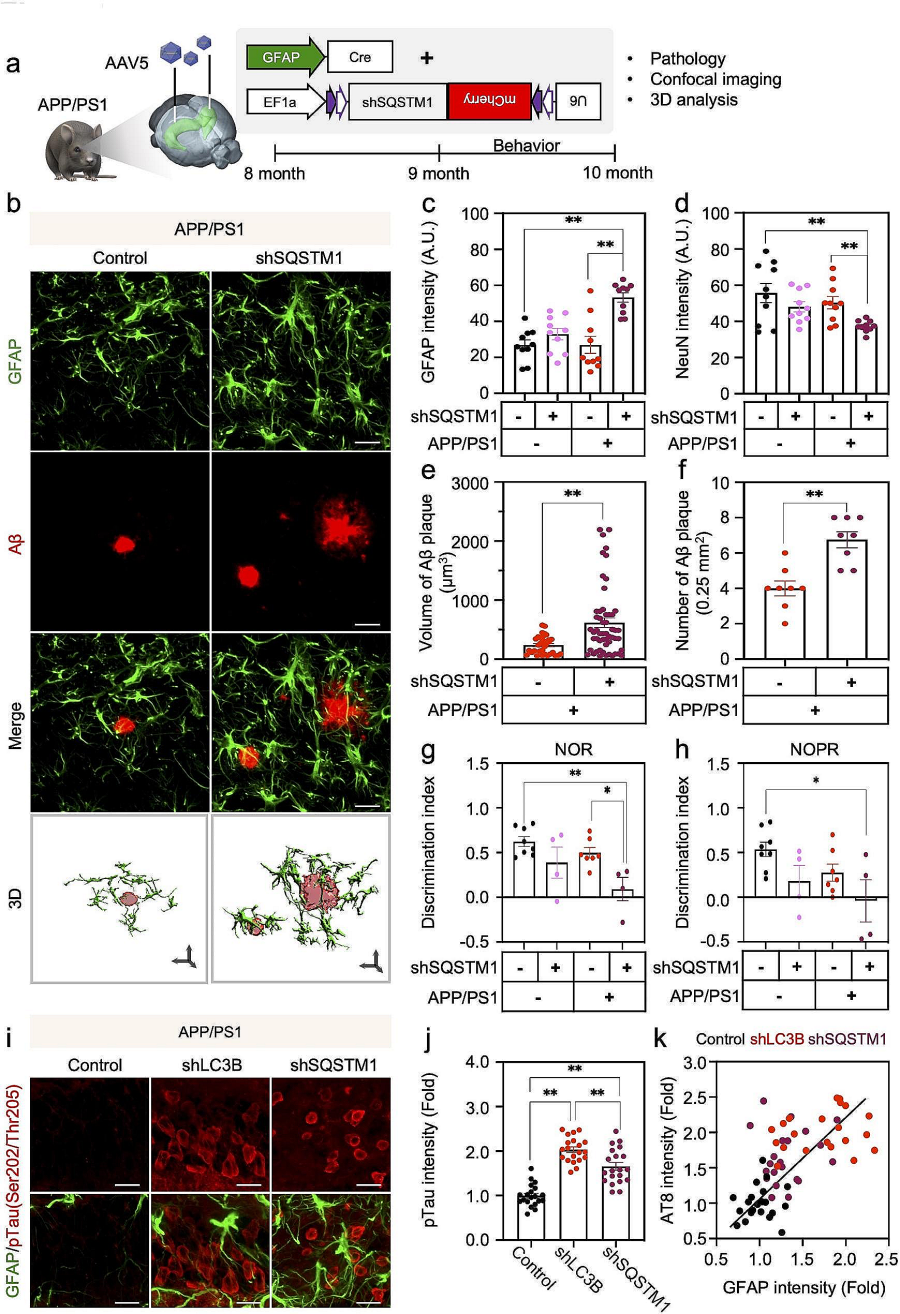
Astrocyte-specific knock down of SQSTM1 exacerbates Aβ plaque formation and cognitive impairment in APP/PS1 mice. **a**, Experimental scheme for genetic inhibition of astrocytic autophagy in the hippocampus of APP/PS1 mice: Group 1, APP/PS1 mice with AAV-EF1a-DIO-Control shRNA + AAV-pGFAP-Cre.; Group 2, APP/PS1 mice with AAV-EF1a-DIO-SQSTM1 shRNA + AAV-pGFAP-Cre. **b**, Double immunostaining for GFAP and Aβ plaque in the brain of APP/PS1 mice with or without SQSTM1 shRNA injection. Scale bars: white, 20 μm; black arrows (x, y, & z in 3D view), 2 μm. **c & d**, Quantification of mean intensity of GFAP (**c**) and NeuN (**d**) immunoreactivity in four groups of mice: a total of 10 ROIs measurement from each group of *N* = 4 mice **e**, Quantification of volume of Aβ plaque in two groups of mice: APP/PS1 control, 32 Aβ plaque measurements; APP/PS1 + SQSTM1 shRNA, 54 Aβ plaque measurements from *N* = 4 mice in each group. **f**, Quantification of the number of Aβ plaque in two groups of mice: APP/PS1 control; APP/PS1 + SQSTM1 shRNA, 8 ROIs from *N* = 4 mice in each group. **g & h**, Discrimination index from behavioral analyses of NOR (**g**) and NOPR (**h**) in four groups of mice: WT control, *N* = 8 mice; WT + SQSTM1 shRNA, *N* = 4 mice; APP/PS1 control, *N* = 7 mice; APP/PS1 + SQSTM1 shRNA, *N* = 4 mice. **i**, Double immunostaining for phosphorylated (p)-Tau (AT8: Ser202/Thr205) and GFAP in the hippocampus of LC3B shRNA or SQSTM1 shRNA virus-injected APP/PS1 mice. Scale bars (white): 20 μm. **j**, Quantification of p-Tau (Ser202/Thr205) intensity in neurons of DG in three groups of mice: a total of 20 ROIs measurements from *N* = 3 mice in each group. **k**, Correlation analysis between GFAP and p-Tau (Ser202/Thr205) intensities (derived from panel j). Data are presented as mean ± SEM. Significantly different at *, *p* < 0.05; **, *p* < 0.01

To further investigate the memory-related behavioral changes on gene-silencing of *MAP1LC3B*/*LC3B* in APP/PS1 mice, we performed open field test, novel object recognition test (NOR) and novel object place recognition test (NOPR) 3 weeks after virus injection (Fig. 6h). We found that the mild impairments of object recognition memory and object place recognition memory shown in LC3B shRNA-infected WT mice or in scrambled shRNA-injected APP/PS1 mice further aggravated by astrocytic knockdown of LC3B in the brain of APP/PS1 mice (Fig. 6i, j). These cellular and behavioral aggravations of AD phenotypes by astrocytic autophagy impairment were also evidenced by astrocytic SQSTM1 knockdown in APP/PS1 mice (Fig. 7a-h, Supplementary Fig. 11a-e). More interestingly, the astrocytic autophagy depletion through the knockdown of LC3B and SQSTM1 markedly increased the levels of tau phosphorylation at serine 202 and threonine 205 sites of paired helical filament (Fig. 7i-k). These results indicate that astrocytic autophagy activation in amyloidogenic mouse model, APP/PS1 mice, is critical for attenuating the transformation into severe cognitive impairment as well as the increase of GFAP-positive astrocytes, plaque over-production and tauopathy in APP/PS1 mice.

### Astrocyte-specific overexpression of LC3B reduces Aβ plaques and restores cognitive function in APP/PS1 mice

**Fig. 8:**
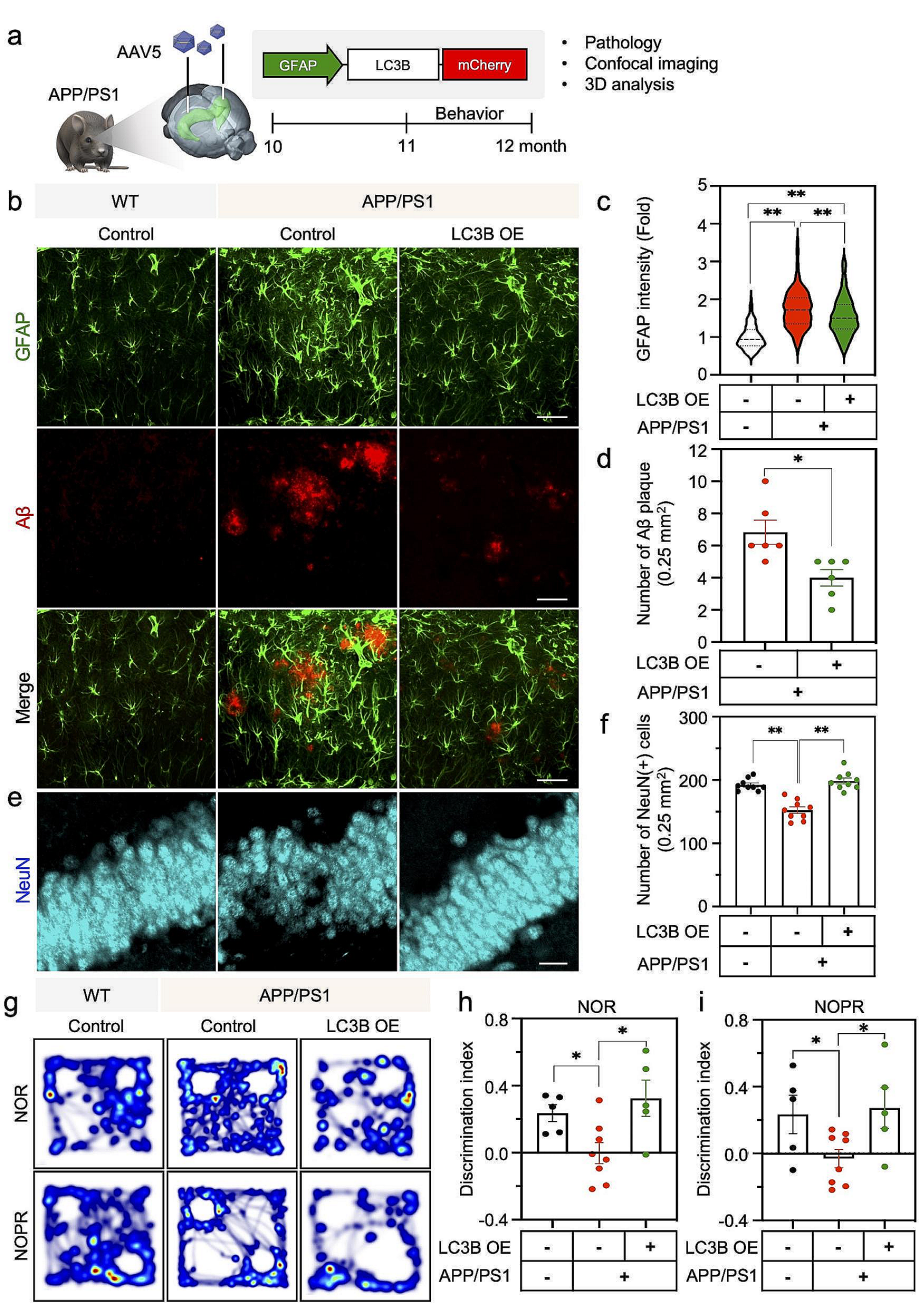
MAP1LC3B/LC3B overexpression ameliorates neuropathology and improves cognitive function in APP/PS1 mice. **a**, Experimental scheme for overexpression of LC3B in the hippocampus of three groups of mice: Group 1, WT + AAV-GFAP-mCherry; Group 2, APP/PS1 + AAV-GFAP-mCherry; Group 3, APP/PS1 + AAV-GFAP-LC3B-mCherry. **b**, Representative immunofluorescence image for GFAP-positive astrocyte (green) and Aβ plaque (red) in the brain of three groups of mice. Scale bars: 50 μm (1st to 3rd raw of images) and 20 μm (4th raw of images). **c & d**, Quantification of GFAP immunoreactivity (**c**) and number of Aβ plaque (**d**). Cell counts: panel c, 150 cell counts from each group of *N* = 3 mice; panel d, 6 ROIs (0.25 mm^2^) from each group of *N* = 3 mice. **e**, Astrocyte-specific overexpression of LC3B restores NeuN immunoreactivity in the hippocampus of APP/PS1 mice. **f**, Astrocyte-specific overexpression of LC3B restores the number of NeuN-positive neurons in APP/PS1 mice. A total of 10 ROIs cell from *N* = 3 mice in each group. **g**, Representative heatmaps of NOR and NOPR behavioral tests in three group of mice. **h & i**, LC3B overexpression rescues cognitive (represented by NOR) (**h**) and spatial memory function (represented by NOPR) (**i**) in APP/PS1 mice. Cases: WT + AAV-GFAP-mCherry, *N* = 5 mice; APP/PS1 + AAV-GFAP-mCherry, *N* = 8 mice; APP/PS1 + AAV-GFAP-LC3B, *N* = 5 mice. Data are presented as mean ± SEM. Significantly different at *, *p* < 0.05; **, *p* < 0.01

In order to further determine whether the gain of LC3B function in astrocytes can ameliorate the Aβ-associated pathology in an animal model of AD, we transduced AAV with GFAP promoter-driven LC3B overexpression vector (AAV-pGFAP-LC3B) to the hippocampus of WT and APP/PS1 mice. (Fig. 8a, Supplementary Fig. 13a-g). We examined the number of Aβ plaques in the brain of APP/PS1 mice at 8 weeks after AAV injection. As expected, astrocyte-specific overexpression of LC3B significantly decreased the number of Aβ plaques in the hippocampus of APP/PS1 compared to control APP/PS1 mice (Fig. 8d). Notably, astrocyte-specific overexpression of LC3B lowered the intensity of GFAP-positive hypertrophic astrocytes in the hippocampus than control APP/PS1 mice (Fig. 8c). Moreover, astrocyte-specific overexpression of LC3B recovered the number of NeuN-positive neurons in the hippocampus of APP/PS1 mice (Fig. 8f). In addition, we performed NOR and NOPR tests to examine the effect of astrocyte-specific overexpression of LC3B on behavioral phenotypes of APP/PS1 mice (Fig. 8a, g-i). The astrocyte-specific LC3B overexpression improved cognitive and spatial memory function in APP/PS1 mice compared to control APP/PS1 mice (Fig. 8g-i).

**Fig. 9:**
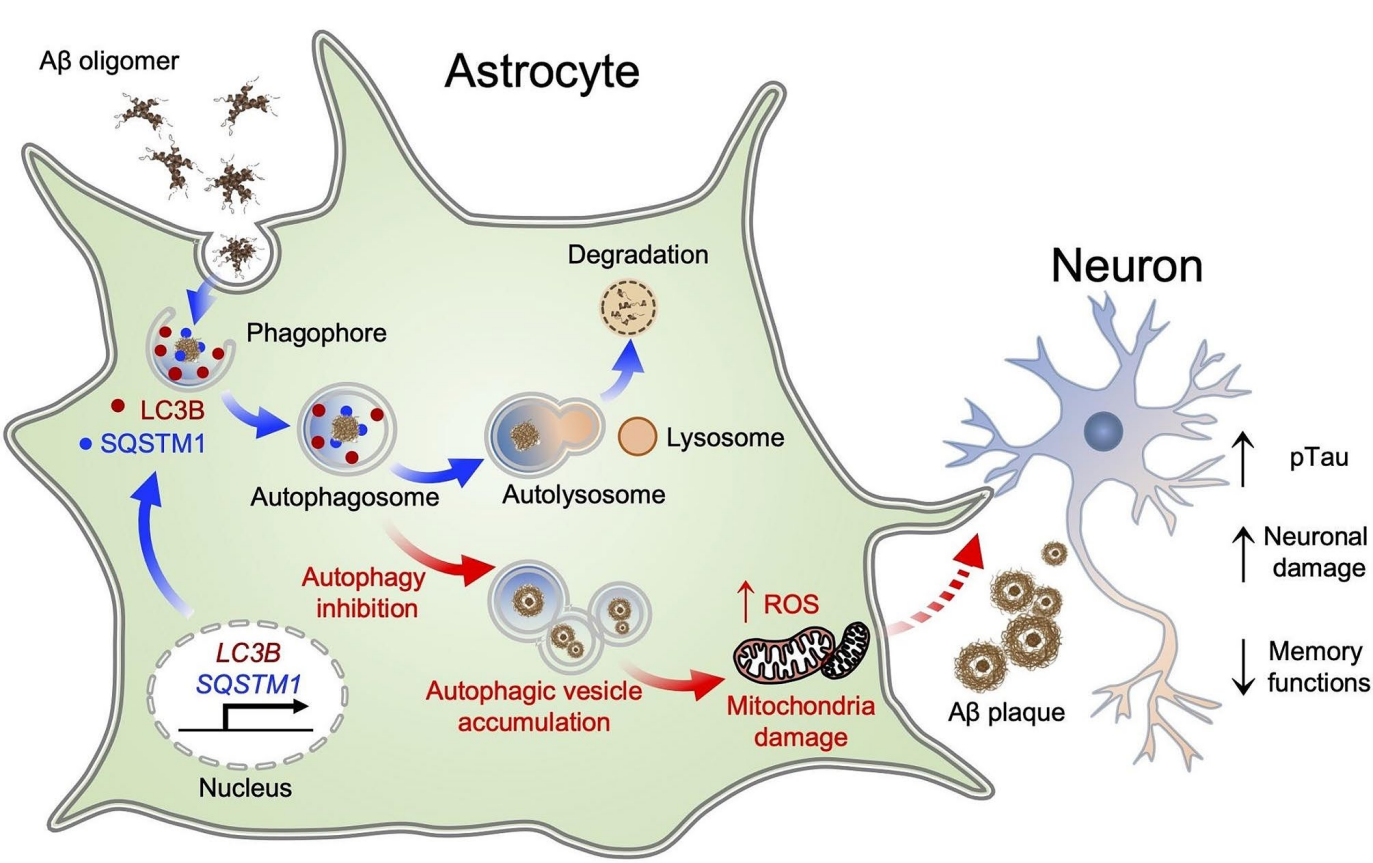
Astrocytes respond to Aβ oligomer-induced stress and undergo plastic changes in the autophagy processes to remove Aβ oligomer. Aβ oligomer transiently induces expression of *MAP1LC3B/LC3B* gene and also turns on a prolonged transcription of *SQSTM1* gene in astrocytes. These components facilitate Aβ degradation via induced astrocytic autophagy pathway. Pharmacological inhibition of autophagy function exacerbates mitochondrial dysfunction and oxidative stress, and leads to astrocytic cell death. When astrocytic autophagy plasticity is impaired by the loss of LC3B and SQSTM1 function, Aβ aggregations and GFAP-positive astrocytes are increased in AD mice, consequently leading to accelerated neuronal damage and memory dysfunction

In order to examine whether LC3B OE plays a beneficial role via a reduction of ROS/peroxide in astrocytes, we performed new experiments in vitro. Human astrocytes were transiently transfected with LC3B plasmids for 24 h and then cells were exposed to Aβ (1µM) for 24 h. ROS was measured by MitoSOX, a mitochondrial ROS marker, and cell death was detected by active caspase-3 immunoreactivity. As a result, we found that LC3B OE reduces Aβ-induced mitochondrial ROS and active caspase-3 levels in astrocytes (Supplementary Fig. 13d and e). These results indicate that LC3B-dependent astrocytic autophagy pathway plays a beneficial function in reducing Aβ pathology and improving behavioral symptoms in AD mice.

## Discussion

### Astrocytes exhibit autophagy plasticity by modulating autophagy-related gene expression in response to Aβ

We discovered that astrocytes possess an inducible autophagic plasticity in response to Aβ, and this regulatory mechanism exhibits a pivotal function in removing Aβ plaques. Both monomeric form and oligomeric form of Aβ increase the expression of autophagy components, *LC3B* and *SQSTM1*, in a transient manner. The basal expression level of autophagy machinery is maintained at a lower level in astrocytes than in neurons [44, 45]. Interestingly, the expression of autophagy machinery in astrocytes is induced in a stimulus-dependent manner (a plasticity of gene induction) to maintain metabolic processes efficiently and to cope with toxic stresses appropriately in AD. In this context, we introduce the autophagy plasticity of astrocytes in response to AD stresses such as Aβ oligomer treatment. In the case of neuronal synaptic plasticity, it is known that stimulus-dependent *de novo* gene expression is associated with brain functions. Though it has not yet been clearly identified whether astrocytes harbor stimulus-dependent *de novo* gene expression, our current study suggests that astrocytes can express autophagy-related genes in response to Aβ temporally and sequentially. In this context, we propose that Aβ-dependent induction of autophagy-associated genes can facilitate autophagy function to cope with Aβ burden, and this event may be regarded as astrocytic autophagy plasticity. In the present study, we verified that the acceleration of autophagy activity is consistently evidenced in various experimental models of cultured astrocytes, brain tissue of AD models, and human AD patients. In addition, using various molecular tools such as RFP-GFP-LC3B (a reporter for monitoring autophagy flux), we confirmed that astrocytes facilitate autophagic flux via LC3B- and SQSTM1-dependent initiation and elongation of autophagic vesicles in response to Aβ oligomer. On the other hand, we confirmed that Aβ-induced autophagy causes long-lasting alterations of astrocyte functions such as turning on the urea cycle to maintain mitochondrial homeostasis and preventing entrance into severe states of neuropathology under AD stresses [18]. In the current study, we demonstrate that inhibition of LC3B and SQSTM1-dependent autophagy pathway reduced the production of urea cycle-associated metabolites such as arginine, aspartate, ornithine, and putrescine. When we dysregulate the astrocyte-specific autophagy function under AD stresses, the homeostasis of metabolic properties such as mitochondrial membrane potential, redox status, and cell viability is disrupted. Moreover, the pathological hallmarks of AD including Aβ plaque formation, reactive astrogliosis, and neuronal damage, are aggravated [46]. These results indicate that astrocytic autophagy plasticity is a necessary event of a distinct metabolic feature in AD and plays a beneficial role to cope with AD-related stresses [47]. Our in vitro data proves, in part, that LC3B OE may provide a beneficial role for cell survival by reducing oxidative stress in astrocytes in response to Aβ. However, a precise mechanism on how LC3B-mediated autophagy reduces ROS and cellular toxicity remains to be investigated in future studies.

### GFAP-positive astrocytes play a dichotomous role in neuropathogenesis

Concurrent with previous reports, our current study has delineated the dual aspects of reactive astrocytes, which are triggered by AD-related toxic molecules such as Aβ [2, 6, 12]. Initially, astrocytes activate detoxifying mechanisms against Aβ toxins by taking up Aβ and by turning on the autophagy pathway and urea cycle, leading to the entrance of putrescine degradation pathway [18]. These resilient features of astrocytes are consistently observed in our recently reported mouse model of reactive astrocytes, GiD, in which astrocytes show both resiliency through autophagy mechanism in the toxin-mediated cell-death system and transformation into reactive states, instead of cell death [6]. As shown in our current results, when the astrocytic autophagy is depleted in APP/PS1 mice, the pathologies of AD such as Aβ plaques, GFAP-positive astrocytes, and neurodegeneration are significantly escalated. The protective function of astrocytes was also confirmed in a previous report showing that depletion of astrocytes acutely exacerbates the pathology of brain diseases [48]. On the other hand, reactive astrocytes excessively synthesize and release detrimental molecules of GABA/ammonia/H_2_O_2_ to cause AD-like symptoms, as shown in previous studies [6, 7, 12]. We propose that astrocytes act as a double-edged sword in AD and these dichotomous aspects of reactive astrocytes co-exist in the brain. In this context, we have verified that inhibition of LC3B and SQSTM1-dependent autophagy pathway reduces mitochondrial membrane potential and exacerbates oxidative stress in Aβ oligomer-treated astrocytes. As a result, the autophagy dysfunction leads to the death of astrocytes. These results imply that induction of autophagy-associated genes and an increase of autophagy flux by Aβ is indispensable for the survival of astrocytes under AD proteotoxic stress.

## Conclusions

Taken together, our studies identify the underlying molecular mechanisms of astrocytic autophagy plasticity and provide critical insights for enhancing the beneficial effects of reactive astrocytes while minimizing the detrimental effects by reactive astrocytes to ameliorate the pathology of AD. In line with this new concept, the pharmacological and genetic modulation of astrocytic autophagy pathway can be considered for boosting the detoxifying machinery of astrocytes under AD stresses and may be a therapeutic strategy for ameliorating AD pathology.

### Electronic supplementary material

Below is the link to the electronic supplementary material.

Supplementary Material 1

## Data Availability

The RNA sequencing data generated in this study deposited to the GEO data base (Accession number : GSE267554, pending until June 30). All supporting information and data are available in the article and supplementary files.

## Abbreviations

LC3B: MAP1LC3B/LC3B
SQSTM1: Sequestosome1
MAO-B: Monoamine oxidase-B
GABA: Gamma-aminobutyric acid
3-MA: 3-methyladenine
CQ: Chloroquine
AAV: Adeno-associated virus
BECN1: Beclin1
GFAP: Glial fibrillary acidic protein
S100B: Calcium-binding protein B
MAP2: Microtubule associated protein 2
NeuN: Neuronal nuclear antigen
NPCAD: NeuroPathologically and Clinically Diagnosed AD-MCI
SAD: Severe AD
ACTB: ACTB/β-actin
TUBB3: TUBB3/tubulin
Aβ: Amyloid beta
Aβ oligomer: Oligomeric form of Aβ
Aβ monomer: Monomeric form of Aβ
AD: Alzheimer’s disease
RNA-seq: RNA sequencing
TEM: Transmission electron microscope
NOR: Novel object recognition test
NOPR: Novel object place recognition test
ALS: Amyotrophic lateral sclerosis
HD: Huntington’s disease
PD: Parkinson’s disease
APP/PS1: APPswe/PSEN1dE9
PFA: Paraformaldehyde
qPCR: Quantitative real time-PCR
DCF-DA: Cell-permeable non-florescent probe 2′,7′-Dichlorofluorescin diacetate
DAPI: 4′,6-diamidino-2-phenylindole
DG: Dentate gyrus
AP: Anterior-posterior
ML: Medial lateral
DV: Dorsal ventral
SD: Standard deviation
PITC: Phenylisothiocyanate
SEM: Standard error of the mean
DMSO: Dimethyl sulfoxide
HRP: Horseradish peroxidase
MTS: 3-(4,5-dimethylthiazol-2-yl)-5-(3-carboxy-methoxyphenyl)-2-(4-sulfophenyl)-2 H-tetazolium inner salt
HBSS: Hanks′ Balanced Salt solution
MRM: Multiple reaction monitoring
ANOVA: A one-way analysis of variance
GFAP: Glial fibrillary acidic protein
NeuN: Neuronal nuclear antigen
PE: Phosphatidylethanolamine
LPS: Lipopolysaccharide
TNF: Tumor necrosis factor
NPCAD: NeuroPathologically and Clinically diagnosed AD
iDTR: Inducible simian diphtheria toxin receptor
E64D: Epoxysuccinyl peptide and an inhibitor of cysteine protease
E/P: E64D/Pepstatin A
CQ: Chloroquine
LC/MS: Liquid chromatography/mass spectrometry
