# Supplementary material for: Astrocytic autophagy plasticity modulates Aβ clearance and cognitive function in Alzheimer’s disease
Source: Mol Neurodegener. 2024 Jul 23;19:55. doi: 10.1186/s13024-024-00740-w (PMC11267931; doi:10.1186/s13024-024-00740-w)
Supplement: Supplementary file 1 — Supplementary Material 1 [file 13024_2024_740_MOESM1_ESM.docx]

**Supplementary Information**

**Astrocytic autophagy plasticity modulates Aβ clearance and cognitive function in Alzheimer’s disease**

Suhyun Kim^1†^, Heejung Chun^2†^, Yunha Kim^1†^, Yeyun Kim^1,3^, Uiyeol Park^1,4^, Jiyeon Chu^1,3^, Mridula Bhalla^5,6^, Seung-Hye Choi^1^, Ali Yousefian-Jazi^1^, Sojung Kim^1^, Seung Jae Hyeon^1^, Seungchan Kim^1^, Yeonseo Kim^7^, Yeon Ha Ju^4,5^, Seung Eun Lee^8^, Hyunbeom Lee^7^, Kyungeun Lee^9^, Soo-Jin Oh^1^, Eun Mi Hwang^10^, Junghee Lee^11,12#^, C. Justin Lee^4,5#^, and Hoon Ryu^1#^

**Supplementary Table 1. Detailed information on the statistical analysis in main figures.**

| **Figure No.** | **Result from statistical analysis** |
| --- | --- |
| 1b | **GFAP**  Aβ(-)(1 ± 0.062), Aβ(+)(1.534 ± 0.093)  A total of 400 cell counts, 50 cells/well, *n* = 8 wells in each group.  Unpaired two-tailed *t*-test, *p<*0.001.  **MAP2**  Aβ(-)(1 ± 0.014), Aβ(+)(0.844 ± 0.043)  A total of 400 cell counts, 50 cells/well, *n* = 8 wells in each group.  Unpaired two-tailed *t*-test, *p*<0.001. |
| 1d | **LC3B**  Aβ(-)(43.09 ± 1.881), Aβ(+)(108.8 ± 2.594)  A total of 200 cell counts, 50 cells/well, *n* = 4 wells in each group.  Unpaired two-tailed *t*-test, *p*<0.001.  **Simple liner regression test**  Aβ(-)(*F*=36.86, *p*<0.001, *R^2^*=0.156), Aβ(+)(*F*=32.79, *p*<0.001, *R^2^*=0.142)  A total of 200 cell counts, 50 cells/well, *n* = 4 wells in each group. |
| 1f | **SQSTM1**  Aβ(-)(20.24 ± 1.137), Aβ(+)(78.78 ± 2.151)  A total of 200 cell counts, 50 cells/well, *n* = 4 wells in each group.  Unpaired two-tailed *t*-test, *p*<0.001.  **Simple liner regression test**  Aβ(-)(*F*=1.163, *p*=0.686, *R^2^*<0.001), Aβ(+)(*F*=25.16, *p*<0.001, *R^2^*=0.113)  A total of 200 cell counts, 50 cells/well, *n* = 4 wells in each group. |
| 1h | **LC3B**  Aβ(-)(93.31 ± 3.861), Aβ(+)(24.94 ± 1.883)  A total of 200 cell counts, 50 cells/well, *n* = 4 wells in each group.  Unpaired two-tailed *t*-test, *p*<0.001  **Simple liner regression test**  Aβ(-)(*F*=54.82, *p*<0.001, *R^2^*=0.217), Aβ(+)(*F*=106.5, *p*<0.001, *R^2^*=0.350)  A total of 200 cell counts, 50 cells/well, *n* = 4 wells in each group. |
| 1j | **SQSTM1**  Aβ(-)(125.5 ± 2.088), Aβ(+)(77.75 ± 1.919)  A total of 200 cell counts, 50 cells/well, *n* = 4 wells in each group.  Unpaired two-tailed *t*-test, *p*<0.001.  **Simple liner regression test**  Aβ(-)(*F*=138.3, *p*<0.001, *R^2^*=0.411), Aβ(+)(*F*=150.6, *p*<0.001, *R^2^*=0.432)  A total of 200 cell counts, 50 cells/well, *n* = 4 wells in each group. |
| 2d | **LC3B**  0 hr (1 ± 0.016), 1 hr (1.390 ± 0.055), 3 hr (1.318 ± 0.104), 6 hr (1.064 ± 0.081), 9 hr (1.079 ± 0.027), 12 hr (0.903 ± 0.012)  One-way ANOVA with Tukey’s multiple comparisons test; *F*=9.638, *p*<0.001.  0 hr *vs* 1 hr, *p*<0.001; 0 hr *vs* 3 hr, *p*=0.006.  **SQSTM1**  0 hr (1 ± 0.027), 1 hr (1.244 ± 0.078), 3 hr (1.370 ± 0.145), 6 hr (1.215 ± 0.445), 9 hr (1.492 ± 0.129), 12 hr (1.674 ± 0.180)  One-way ANOVA with Tukey’s multiple comparisons test; *F*=4.170, *p*=0.0025.  0 hr *vs* 9 hr, *p*=0.041; 0hr *vs* 12hr, *p*=0.001.  **Beclin 1 (BECN1)**  0 hr (1 ± 0.010), 1 hr (0.907 ± 0.094), 3 hr (1.089 ± 0.057), 6 hr (0.993 ± 0.053), 9 hr (0.919 ± 0.216), 12 hr (0.944 ± 0.021)  One-way ANOVA with Tukey’s multiple comparisons test; *F*=1.90, *p*=0.106.  All data were normalized to average of mRNA level at 0 hr.  All data were generated from 4 separate experiment, triplicate/experiment. |
| 2f | **LC3B-II/ATCB**  1 hr (1.335 ± 0.154), 2 hr (1.206 ± 0.107), 4 hr (0.897 ± 0.160), 8 hr (0.701 ± 0.104), 12 hr (0.720 ± 0.194), 24 hr (0.712 ± 0.110), 48 hr (0.932 ± 0.128), 72 hr (0.863 ± 0.269), 96 hr (0.755 ± 0.087), 120 hr (0.629 ± 0.109)  Unpaired two-tailed *t*-test; 0 hr *vs* 1hr, *p*=0.028; 0 hr *vs* 2 hr, *p*=0.039; 0 hr *vs* 8hr, *p*=0.012; 0 hr *vs* 12 hr, *p*=0.033; 0 hr *vs* 24 hr, *p*=0.016; 0 hr *vs* 96 hr, *p*=0.013; 0 hr *vs* 120 hr, *p*=0.007.  **SQSTM1/ATCB**  1 hr (0.967 ± 0.106), 2 hr (1.463 ± 0.251), 4 hr (2.229 ± 0.109), 8 hr (6.154 ± 0.926), 12 hr (6.946 ± 0.894), 24 hr (4.812 ± 0.597), 48 hr (3.457 ± 0.426), 72 hr (1.956 ± 0.389), 96 hr (1.429 ± 0.340), 120 hr (1.208 ± 0.453)  Unpaired two-tailed *t*-test; 0 hr *vs* 2 hr, *p*=0.043; 0 hr *vs* 8 hr, *p*=0.001; 0 hr *vs* 12 hr, *p*=0.001; 0 hr *vs* 24 hr, *p*=0.006; 0 hr *vs* 48 hr, *p*=0.0007; 0 hr *vs* 72 hr, *p*=0.019.  All data were generated from 3 separate experiment, triplicate/experiment.  All data were normalized to the average of ACTB level. |
| 2h | Control (1 ± 0.03), Aβ monomer (1.328 ± 0.067), Aβ oligomer (1.721 ± 0.143)  A total of 20 cell counts, 5 cells/well, *n* = 4 wells in each group.  One-way ANOVA with Tukey’s multiple comparisons test; *F*=15.12, *p=*0.0013.  Unpaired two-tailed *t*-test; Control *vs* Aβ oligomer, *p=*0.001; Aβ monomer *vs* Aβ oligomer, *p=*0.036. Normalized to the average of control. |
| 2i | Control (1 ± 0.143), Aβ monomer (1.845 ± 0.145), Aβ oligomer (3.346 ± 0.207)  A total of 20 cell counts, 5 cells/well, *n* = 4 wells in each group.  One-way ANOVA with Tukey’s multiple comparisons test; *F*=49.98, *p*<0.001; Control *vs* Aβ monomer, *p=*0.015; Control *vs* Aβ oligomer, *p*<0.001; Aβ monomer *vs* Aβ oligomer, *p*<0.001. Normalized to the average of control. |
| 3b | **LC3B-II/ATCB**  Normal (1 ± 0.103, *N* = 11 cases), NPCAD (0.980 ± 0.200, *N* = 11 cases), SAD (1.636 ± 0.229, *N* = 10 cases)  One-way ANOVA with Tukey’s multiple comparisons test; *F*=4.552, *p*=0.019; Normal *vs* SAD, *p*=0.038; NPCAD *vs* SAD, *p*=0.031.  **SQSTM1/ATCB**  Normal (0.889 ± 0.1, *N* = 11 cases), NPCAD (1.025 ± 0.102, *N* = 11 cases), SAD (2.190 ± 0.389, *N* = 10 cases)  One-way ANOVA with Tukey’s multiple comparisons test; *F*=12.18, *p<*0.001. Normal *vs* SAD, *p*=0.001; NPCAD *vs* SAD, *p*=0.003.  All data were normalized to the average of ACTB level. |
| 3d | **GFAP**  Normal (109.9 ± 3.221), NPCAD (138.0 ± 9.101), SAD (165.4 ± 2.314)  One-way ANOVA with Tukey’s multiple comparisons test; *F*=2.536, *p*=0.025.  Normal *vs* NPCAD, *p*=0.012; Normal *vs* SAD, *p*<0.001; NPCAD *vs* SAD, *p*=0.014.  **LC3B**  Normal (178.7 ± 2.130), NPCAD (208.1 ± 5.701), SAD (233.2 ± 8.681)  One-way ANOVA with Tukey’s multiple comparisons test; *F*=1.751, *p<*0.001.  Normal *vs* NPCAD, *p*=0.013; Normal *vs* SAD, *p*<0.001; NPCAD *vs* SAD, *p*=0.033.  One dot represents an average of 12 cells (a total of 60 cells), 12 cells/case, each *N* = 5 cases for normal, NPCAD, and SAD. |
| 3e | **Simple liner regression test**  Normal (*F*=1.173, *p*=0.283, *R^2^*=0.019), NPCAD (*F*=2.790, *p*=0.1, *R^2^*=0.046), SAD (*F*=21.92, *p*<0.001, *R^2^*=0.274), Total (*F*=135.2, *p*<0.001, *R^2^*=0.432)  A total of 60 cell counts, 12 cells/case, *N* = 5 cases in each group. |
| 4a | **Control**  0 hr (100 ± 1.589 %, *n* = 15 wells), 6 hr (98.32 ± 1.900 %, *n* = 15 wells), 12 hr (100 ± 1.391 %, *n* = 15 wells), 18 hr (100 ± 0.663 %, *n* = 15 wells), 24 hr (100 ± 0.304 %, *n* = 15 wells)  One-way ANOVA with Tukey’s multiple comparisons test; *F*=0.439, *p=*0.779.  **Aβ monomer**  0 hr (101.2 ± 1.040 %, *n* = 11 wells), 6 hr (95.77 ± 0.934 %, *n* = 13 wells), 12 hr (95.48 ± 0.966 %, *n* = 11 wells), 18 hr (90.25 ± 1.372 %, *n* = 13 wells), 24 hr (88.39 ± 0.834 %, *n* = 15 wells)  One-way ANOVA with Tukey’s multiple comparisons test; *F*=23.44, *p<*0.001.  0 hr *vs* 6hr, *p*=0.006; 0 hr *vs* 12 hr, *p*=0.005; 0 hr *vs* 18 hr, *p*<0.001; 0 hr *vs* 24 hr, *p*<0.001.  **Aβ oligomer**  0 hr (101.6 ± 1.130 %, *n* = 12 wells), 6 hr (81.73 ± 1.223 %, *n* = 15 wells), 12 hr (80.77 ± 0.846 %, *n* = 14 wells), 18 hr (75.82 ± 1.069 %, *n* = 14 wells), 24 hr (75.35 ± 1.175 %, *n* = 14 wells)  One-way ANOVA with Tukey’s multiple comparisons test; *F*=86.10, *p<*0.001.  0 hr *vs* 6hr, *p*<0.001; 0 hr *vs* 12 hr, *p*<0.001; 0 hr *vs* 18 hr, *p*<0.001; 0 hr *vs* 24 hr, *p*<0.001. |
| 4b | Control (96.80 ± 1.097 %, *n* = 10 wells), Aβ monomer (85.91 ± 2.779 %, *n* = 12 wells), Aβ oligomer (77.52 ± 2.522 %, *n* = 10 wells), E/P (75.63 ± 2.353 %, *n* = 13), Aβ monomer + E/P (67.60 ± 2.301 %, *n* = 13 wells), Aβ oligomer + E/P (63.43 ± 1.512 %, *n* = 15 wells)  One-way ANOVA with Tukey’s multiple comparisons test, *F*=30.45, *p<*0.001.  Control *vs* Aβ monomer, *p*=0.016; Control *vs* Aβ monomer, *p*<0.001; Control *vs* Aβ oligomer, *p*<0.001; Control *vs* E/P, *p*<0.001; Control *vs* Aβ monomer + E/P, *p*<0.001; Control *vs* Aβ oligomer + E/P, *p*<0.001; Aβ monomer *vs* Aβ monomer + E/P, *p*<0.001; Aβ oligomer *vs* Aβ oligomer + E/P, *p*=0.001. |
| 4c | Control (100 ± 4.044 %), 3MA (91.530 ± 5.757 %), Aβ oligomer (91.722 ± 4.992 %), 3MA+Aβ oligomer (66.313 ± 1.819 %)  Unpaired two-tailed *t*-test; Control *vs* 3MA+ Aβ oligomer, *p*<0.001.  All data were generated from *n* = 4 wells in each group. |
| 4e | **Survival**  Control (92.90 ± 0.527 %), CQ (82.79 ± 2.201 %), E/P (80.60 ± 4.371 %), Aβ oligomer (62.88 ± 3.890 %), CQ+Aβ oligomer (56.55 ± 1.715 %), E/P+Aβ oligomer (43.46 ± 3.595 %)  One-way ANOVA with Tukey’s multiple comparisons test; *F*=13.55, *p<*0.018.  Control *vs* E/P, *p=*0.040; Control *vs* Aβ oligomer, *p<*0.001; Control *vs* Aβ oligomer + CQ, *p<*0.001; Control *vs* Aβ oligomer + E/P, *p<*0.001; CQ *vs* Aβ oligomer + CQ, *p<*0.001; E/P *vs* Aβ oligomer + E/P, *p<*0.001.  **Apoptosis**  Control (2.498 ± 0.392 %), CQ (9.978 ± 2.048 %), E/P (3.596 ± 1.373 %), Aβ oligomer (15.81 ± 2.620 %), CQ+Aβ-oligomer (15.77 ± 2.020 %), E/P + Aβ oligomer (22.09 ± 0.946 %)  One-way ANOVA with Tukey’s multiple comparisons test; *F*=19.56, *p<*0.001.  Control *vs* Aβ oligomer, *p<*0.001; Control *vs* Aβ oligomer + CQ, *p<*0.001; Control *vs* Aβ oligomer + E/P, *p<*0.001; E/P *vs* Aβ oligomer + E/P, *p<*0.001.  **Necrosis**  Control (0.311 ± 0.196 %), CQ (4.394 ± 0.669 %), E/P (11.60 ± 3.554 %), Aβ oligomer (16.44 ± 2.013 %), CQ+Aβ oligomer (22.42 ± 1.103 %), E/P+Aβ oligomer (26.25 ± 2.694 %)  One-way ANOVA with Tukey’s multiple comparisons test; *F*=23.92, *p<*0.001.  Control *vs* E/P, *p=*0.008; Control *vs* Aβ oligomer, *p<*0.001; Control *vs* Aβ oligomer + CQ, *p<*0.001; Control *vs* Aβ oligomer + E/P, *p<*0.001; CQ *vs* Aβ oligomer + CQ, *p<*0.001; E/P *vs* Aβ oligomer + E/P, *p<*0.001.  **Late apoptosis**  Control (2.927 ± 0.510 %), CQ (2.835 ± 0.628 %), E/P (4.20 ± 0.426 %), Aβ oligomer (4.869 ± 0.731 %), CQ + Aβ oligomer (5.262 ± 0.280 %), E/P + Aβ oligomer (8.210 ± 1.641 %)  One-way ANOVA with Tukey’s multiple comparisons test; *F*=5.655, *p=*0.0014.  Control *vs* Aβ oligomer + E/P, *p=*0.001; E/P *vs* Aβ oligomer + E/P, *p=*0.024.  All data were generated 5 wells in each group. |
| 4h | **Autophagosome**  Control (1.686 ± 0.557 μm^2^, *n* = 8 cells), CQ (10.60 ± 1.605 μm^2^, *n* = 10 cells), E/P (2.464 ± 0.459 μm^2^, *n* = 10 cells), Aβ (4.637 ± 1.124 μm^2^, *n* = 10 cells), Aβ + CQ (23.70 ± 6.784 μm^2^, *n* = 7 cells), Aβ + E/P (14.90 ± 3.856 μm^2^, *n* = 9 cells),  One-way ANOVA with Tukey’s multiple comparisons test; *F*=8.062, *p<*0.001.  Control *vs* Aβ oligomer + CQ, *p=*0.025; Control *vs* Aβ oligomer + E/P, *p<*0.001; CQ *vs* Aβ oligomer + CQ, *p=*0.030; E/P *vs* Aβ oligomer + E/P, *p=*0.026.  **Autolysosome**  Control (1.711 ± 1.230 μm^2^, *n* = 8 cells), CQ (2.119 ± 0.788 μm^2^, *n* = 10 cells), E/P (0.813 ± 0.203 μm^2^, *n* = 10 cells), Aβ (3.551 ± 0.977 μm^2^, *n* = 10 cells), Aβ + CQ (10.22 ± 2.312 μm^2^, *n* = 7 cells), Aβ + E/P (13.95 ± 3.111 μm^2^, *n* = 9 cells)  One-way ANOVA with Tukey’s multiple comparisons test; *F*=10.64, *p<*0.001.  Control *vs* Aβ oligomer + CQ, *p=0*.001; Control *vs* Aβ oligomer + E/P, *p<*0.001; CQ *vs* Aβ oligomer + CQ, *p=*0.004; E/P *vs* Aβ oligomer + E/P, *p<*0.001. |
| 4j | **Size**  Control (0.132 ± 0.012 μm^2^), CQ (12hr) (0.336 ± 0.049 μm^2^), Aβ (12hr) (0.376 ± 0.065 μm^2^), Aβ + CQ (12hr) (1.779 ± 0.304 μm^2^)  One-way ANOVA with Tukey’s multiple comparisons test; *F* = 22.94, *p<* 0.001.  Control *vs* Aβ oligomer + CQ, *p<0*.001; CQ *vs* Aβ oligomer + CQ, *p<*0.001; Aβ oligomer *vs* Aβ oligomer + CQ, *p<0*.001.  **Number**  Control (14.60 ± 1.166), CQ (16.0 ± 1.871), Aβ (20.40 ± 2.960), Aβ + CQ (13.60 ± 2.657)  One-way ANOVA with Tukey’s multiple comparisons test; *F*=1.740, *p=*0.199.  A total of 5 ROIs (100μm^2^) in each group was examined. |
| 5b | **Size**  Control (1 ± 0.046), E/P (0.688 ± 0.075), Aβ oligomer (0.713 ± 0.003), Aβ oligomer + E/P (0.497 ± 0.015)  One-way ANOVA with Tukey’s multiple comparisons test; *F*=21.04, *p<*0.001.  Control *vs* E/P, *p=*0.005; Control *vs* Aβ oligomer, *p=*0.008; Control *vs* Aβ oligomer + E/P, *p<*0.001; Aβ oligomer *vs* Aβ oligomer + E/P, *p=*0.039.  A total of 15 cell counts, 5 cells/well, *n* = 3 wells in each group.  **Length**  Control (1 ± 0.058), E/P (0.646 ± 0.080), Aβ oligomer (0.585 ± 0.01), Aβ oligomer + E/P (0.343 ± 0.017)  One-way ANOVA with Tukey’s multiple comparisons test; *F*=28.50, *p<*0.001.  Control *vs* E/P, *p=*0.005; Control *vs* Aβ oligomer, *p=*0.001; Control *vs* Aβ oligomer + E/P, *p<*0.001; Aβ oligomer *vs* Aβ oligomer + E/P, *p*=0.039.  A total of 15 cell counts, 5 cells/well, *n* = 3 wells in each group.  **Branch junction**  Control (1 ± 0.388), E/P (0.509 ± 0.129), Aβ oligomer (0.459 ± 0.075), Aβ oligomer + E/P (0.266 ± 0.023)  One-way ANOVA with Tukey’s multiple comparisons test; *F*=15.97, *p=*0.001.  Control *vs* E/P, *p=*0.009; Control *vs* Aβ oligomer, *p=*0.005; Control *vs* Aβ oligomer + E/P, *p<*0.001.  Unpaired two-tailed *t*-test; Aβ oligomer *vs* Aβ oligomer + E/P, *p*=0.012.  A total of 15 cell counts, 5 cells/well, *n* = 3 wells in each group.  **Mito-SOX**  Control (1 ± 0.069), E/P (1.132 ± 0.097), Aβ oligomer (1.446 ± 0.057), Aβ oligomer + E/P (1.810 ± 0.079)  One-way ANOVA with Tukey’s multiple comparisons test; *F*=21.57, *p<*0.001.  Control *vs* Aβ oligomer, *p=*0.0075; Control *vs* Aβ oligomer + E/P, *p<*0.001; E/P *vs* Aβ oligomer + E/P, *p<*0.001; Aβ oligomer *vs* Aβ oligomer + E/P, *p=*0.027.  A total of 20 cell counts, 5 cells/well, *n* = 4 wells in each group.  Normalized to the average of control. |
| 5d | **Size**  Control (0.842 ± 0.054 μm^2^, *n* = 34 mitochondria), CQ (12hr) (0.816 ± 0.065 μm^2^, *n* = 31 mitochondria), Aβ oligomer (12hr) (0.732 ± 0.066 μm^2^, *n* = 29 mitochondria,), Aβ oligomer + CQ (12hr) (0.480 ± 0.053 μm^2^, *n* = 29 mitochondria)  A total of 5 ROIs (100 μm^2^) in each group was examined.  One-way ANOVA with Tukey’s multiple comparisons test, *F*=7.395, *p<*0.001.  Control *vs* Aβ oligomer + CQ, *p<*0.001; CQ *vs* Aβ oligomer + CQ, *p<*0.001; Aβ oligomer *vs* Aβ oligomer + CQ, *p=*0.024. |
| 5e | Control (0.179 ± 0.003 μM, *n* = 6 wells), Aβ oligomer (0.207 ± 0.004 μM, *n* = 6 wells), Aβ oligomer + CQ (0.294 ± 0.007 μM, *n* = 6 wells)  One-way ANOVA with Tukey’s multiple comparisons test, *F*=114.4, *p<*0.001.  Control *vs* Aβ oligomer, *p=*0.007; Control *vs* Aβ oligomer + CQ, *p<*0.001; Aβ oligomer *vs* Aβ oligomer + CQ, *p<*0.001. |
| 5f | Control (1 ± 0.057, *n* = 6 wells), Aβ oligomer (1.355 ± 0.143, *n* = 8 wells), Aβ oligomer + KDS2010 (0.874 ± 0.036, *n* = 8 wells)  Normalized to the average of intensity of control.  One-way ANOVA with Tukey’s multiple comparisons test, *F*=7.041, *p=*0.0052.  Unpaired two-tailed *t*-test; Control *vs* Aβ oligomer, *p*=0.032; Aβ oligomer *vs* Aβ oligomer + KDS2010, *p*=0.0029. |
| 6d | WT + Control (703.2 ± 44.15 μm^3^, *n* = 31 cells), WT + LC3B shRNA (725.6 ± 50.70 μm^3^, *n* = 40 cells), APP/PS1 + Control (937.2 ± 105.4 μm^3^, *n* = 27 cells), APP/PS1 + LC3B shRNA (1435 ± 125.3 μm^3^, *n* = 36 cells)  A total of 8 ROIs (0.25 mm^2^) was examined from *N* = 4 mice in each group.  One-way ANOVA with Tukey’s multiple comparisons test; *F*= 19.12, *p<*0.001.  WT + Control *vs* APP/PS1 + LC3B shRNA, *p*<0.001; LC3B shRNA *vs* APP/PS1 + LC3B shRNA, *p*<0.001, APP/PS1 + Control *vs* APP/PS1 + LC3B shRNA, *p*<0.001. |
| 6e | APP/PS1 + Control (344.8 ± 33.09 μm^3^, 45 plaques), APP/PS1 + LC3B shRNA (720.3 ± 72.68 μm^3^, 53 plaques)  A total of 8 ROIs (0.25 mm^2^) was examined from *N* = 4 mice in each group.  Unpaired two-tailed *t*-test; *p<*0.001. |
| 6g | WT + Control (102.8 ± 3.760), WT + LC3B shRNA (100.2 ± 4.011), APP/PS1+ Control (73.99 ± 3.615), APP/PS1 + LC3B shRNA (48.01 ± 2.763)  A total of 20 ROIs (0.25 mm^2^) was examined from *N* = 4 mice in each group.  One-way ANOVA with Tukey’s multiple comparisons test, *F*=51.78, *p<*0.001.  WT + Control *vs* APP/PS1 + Control, *p*<0.001; APP/PS1 + Control *vs* APP/PS1 + LC3B shRNA, *p*<0.001, WT + Control *vs* APP/PS1 + LC3B shRNA, *p*<0.001. |
| 6i | WT + Control (0.623 ± 0.055, *N* = 8 mice), WT + LC3B shRNA (0.481 ± 0.075, *N* = 4 mice), APP/PS1 + Control (0.497 ± 0.058, *N* = 7 mice), APP/PS1 + LC3B shRNA + (0.131 ± 0.153, *N* = 4 mice)  One-way ANOVA with Tukey’s multiple comparisons test; *F*=6.193, *p=*0.004.  WT + Control *vs* APP/PS1 + LC3B shRNA, *p*=0.002; APP/PS1 + Control *vs* APP/PS1 + LC3B shRNA, *p=*0.026.  Unpaired two-tailed *t*-test; WT + Control *vs* APP/PS1 + LC3B shRNA, *p*=0.0037; APP/PS1 + Control *vs* APP/PS1 + LC3B shRNA, *p*=0.025.  Discrimination index = (Duration*_(new)_*-Duration*_(old)_*)/Duration*_(total)_* |
| 6j | WT + Control (0.535 ± 0.080, *N* = 8 mice), WT + LC3B shRNA (0.119 ± 0.216, *N* = 4 mice), APP/PS1 + Control (0.275 ± 0.095, *N* = 7 mice), APP/PS1 + LC3B shRNA (-0.169 ± 0.112, *N* = 4 mice)  One-way ANOVA with Tukey’s multiple comparisons test; *F*=3.450, *p=*0.003.  WT + Control *vs* APP/PS1 + LC3B shRNA, *p*=0.002.  Unpaired two-tailed *t*-test; WT + Control *vs* APP/PS1 + LC3B shRNA, *p*=0.048; APP/PS1 + Control *vs* APP/PS1 + LC3B shRNA, *p*=0.017.  Discrimination index = (Duration*_(new)_*-Duration*_(old)_*)/Duration*_(total)_* |
| 7c | WT + Control (26.91 ± 2.776), WT + SQSTM1 shRNA (32.93 ± 3.218), APP/PS1 + Control (26.90 ± 4.702), APP/PS1 + SQSTM1 shRNA (53.26 ± 2.657)  A total of 10 ROIs (0.25mm^2^) was examined from *N* = 4 mice in each group.  One-way ANOVA with Tukey’s multiple comparisons test; *F*=13.23, *p<* 0.001.  WT + Control *vs* APP/PS1 + SQSTM1 shRNA, *p<*0.001; Control + APP/PS1 *vs* APP/PS1 + SQSTM1 shRNA, *p<*0.001. |
| 7d | WT + Control (55.68 ± 5.302), WT + SQSTM1 shRNA (48.05 ± 2.811), APP/PS1 + Control (50.33 ± 3.396), APP/PS1 + SQSTM1 shRNA (37.33 ± 2.657)  A total of 10 ROIs (0.25 mm^2^) was examined from *N* = 4 mice in each group.  One-way ANOVA with Tukey’s multiple comparisons test; *F*=4.894, *p=*0.005.  WT + Control *vs* APP/PS1 + SQSTM1 shRNA, *p<*0.001; APP/PS1 + Control *vs* APP/PS1 + SQSTM1 shRNA, *p*<0.001.  Unpaired two-tailed *t*-test; WT + Control *vs* APP/PS1 + SQSTM1 shRNA, *p=*0.003; APP/PS1 + Control *vs* APP/PS1 + SQSTM1 shRNA, *p=*0.001 |
| 7e | APP/PS1 + Control (237.6 ± 27.52 μm^3^, 32 plaques), APP/PS1 + SQSTM1 shRNA (618.1 ± 79.60 μm^3^, 54 plaques)  A total of 8 ROIs (0.25 mm^2^) was examined from *N* = 4 mice in each group.  Unpaired two-tailed *t*-test; *p*<0.001. |
| 7f | APP/PS1 + Control (4 ± 0.422), APP/PS1 + SQSTM1 shRNA (6.750 ± 0.453)  A total of 8 ROIs (0.25 mm^2^) was examined from *N* = 4 mice in each group.  Unpaired two-tailed *t*-test; *p*< 0.001. |
| 7g | WT + Control (0.620 ± 0.055, *N* = 8 mice), WT + SQSTM1 shRNA (0.388 ± 0.074, *N* = 4 mice), APP/PS1 + Control (0.497 ± 0.058, *N* = 7 mice), APP/PS1 + SQSTM1 shRNA (0.091 ± 0.130, *N* = 4 mice)  One-way ANOVA with Tukey’s multiple comparisons test; *F*=5.617, *p=*0.0063.  WT + Control *vs* APP/PS1 + SQSTM1 shRNA, *p=*0.003; APP/PS1 + Control *vs* APP/PS1 + SQSTM1 shRNA, *p*=0.039.  Discrimination index = (Duration*_(new)_*-Duration*_(old)_*)/Duration*_(total)_* |
| 7h | WT + Control (0.535 ± 0.080, *N* = 8 mice), WT + SQSTM1 shRNA (0.180 ± 0.174, *N* = 4 mice), APP/PS1 + Control (0.275 ± 0.095, *N* = 7 mice), SQSTM1 shRNA + APP/PS1 (-0.040 ± 0.236, *N* = 4 mice)  One-way ANOVA with Tukey’s multiple comparisons test; *F*=3.450, *p=*0.037.  WT + Control *vs* APP/PS1 + SQSTM1 shRNA, *p=*0.029.  Discrimination index = (Duration*_(new)_*-Duration*_(old)_*)/Duration*_(total)_* |
| 7j | APP/PS1 + Control (1 ± 0.054), APP/PS1 + LC3B shRNA (2.031 ± 0.062), APP/PS1 + SQSTM1 shRNA (1.654 ± 0.087)  A total of 20 ROIs (0.01 mm^2^) was examined from *N* = 4 mice in each group.  Normalized to the average of control + APP/PS1.  One-way ANOVA with Tukey’s multiple comparisons test; *F*=55.90, *p<*0.001.  APP/PS1 + Control *vs* APP/PS1 + LC3B shRNA, *p* <0.001; APP/PS1 + Control *vs* APP/PS1 + SQSTM1 shRNA, *p*<0.001; APP/PS1 + Control *vs* APP/PS1 + SQSTM1 shRNA, *p*<0.001; APP/PS1 + LC3B shRNA *vs* APP/PS1 + SQSTM1 shRNA, *p*=0.001. |
| 7k | **Simple liner regression test**  APP/PS1+Control (*F*=0.279, *p=*0.603, *R^2^*=0.014); APP/PS1+ LC3B shRNA (*F*=0.868, *p=*0.363, *R^2^*=0.043); APP/PS1+ SQSTM1 shRNA (*F*=0.351, *p*=0.560, *R^2^*=0.018); Total groups (*F*=41.96, *p*<0.001, *R^2^*=0.407, *n* = 60 cells)  A total of 20 ROIs (0.01 mm^2^) was examined from *N* = 4 mice in each group. |
| 8c | WT + Control (1 ± 0.025), APP/PS1 + Control (1.734 ± 0.041), APP/PS1 + LC3B OE (1.556 ± 0.041)  Normalized to the average of WT + Control.  A total of 150 cell counts was examined from *N* = 3 mice in each group.  One-way ANOVA with Tukey’s multiple comparisons test; *F*=107.7, *p<*0.001.  WT + Control *vs* APP/PS1 +Control, *p<*0.001; WT + Control *vs* APP/PS1 + LC3B OE, *p<*0.001; WT + Control *vs* APP/PS1 + LC3B OE, *p=*0.002. |
| 8d | Control + APP/PS1 (27.33 ± 2.88), LC3 OE + APP/PS1 (17.50 ± 1.92)  A total of 6 ROIs (0.25 mm^2^) was examined from *N* = 3 mice in each group.  Unpaired two-tailed *t*-test; *p* = 0.017. |
| 8f | WT + Control (192.2.8 ± 3.126 cells), APP/PS1 + Control (152.3 ± 5.235 cells), APP/PS1 + LC3B OE (198.5 ± 4.670 cells)  Normalized to the average of WT + Control.  A total of 9 ROIs (0.25 mm^2^) was examined from *N* = 3 mice in each group.  One-way ANOVA with Tukey’s multiple comparisons test; *F*=150.8, *p<*0.001.  WT + Control *vs* APP/PS1 +Control, *p<*0.001; APP/PS1 + Control *vs* APP/PS1 + LC3B OE, *p<*0.001. |
| 8h | WT + Control (0.235 ± 0.049, *N* = 5 mice), APP/PS1 + Control (-0.002 ± 0.063, *N* = 8 mice), APP/PS1 + LC3 OE (0.324 ± 0.107, *N* = 5 mice)  One-way ANOVA with Tukey’s multiple comparisons test; *F*=5.605, *p=*0.015.  APP/PS1 + Control *vs* APP/PS1 + LC3B OE, *p=*0.017.  Unpaired two-tailed *t*-test; WT + Control *vs* APP/PS1 + Control, *p=*0.021.  Discrimination index = (Duration*_(new)_*-Duration*_(old)_*)/Duration*_(total)_* |
| 8i | WT + Control (0.233 ± 0.115, *N* = 5 mice), APP/PS1 + Control (- 0.03 ± 0.054, *N* = 8 mice), APP/PS1 + LC3B OE (0.271 ± 0.122, *N* = 5 mice)  One-way ANOVA with Tukey’s multiple comparisons test; *F*=3.695, *p=*0.049.  Unpaired two-tailed *t*-test; WT + Control *vs* APP/PS1 + Control, *p=*0.039; APP/PS1 + Control *vs* APP/PS1 +LC3B OE, *p=*0.025.  Discrimination index = (Duration*_(new)_*-Duration*_(old)_*)/Duration*_(total)_* |

**Supplementary Table 2. Detailed information on the statistical analysis in supplementary figures.**

| **Figure No.** | **Result from statistical analysis** |
| --- | --- |
| S1b | **Simple linear regression test**  Aβ(-)(*F*=200.9, *p*<0.001, *R^2^*=0.503), Aβ(+)(*F*=188.3, *p*<0.001, *R^2^*=0.487)  A total of 200 cell counts, 50 cells/well, *n* = 4 wells in each group. |
| S1d | **Simple linear regression test**  Aβ(-)(*F*=87.09, *p*<0.001, *R^2^*=0.305), Aβ(+)(*F*=142.5, *p*<0.001, *R^2^* =0.418)  A total of 200 cell counts, 50 cells/well, *n* = 4 wells in each group. |
| S1f | **Simple linear regression test**  Aβ(-)(*F*=32.25, *p*<0.001, *R^2^*=0.140), Aβ(+)(*F*=20.44, *p*<0.001, *R^2^*=0.093)  A total of 200 cell counts, 50 cells/well, *n* = 4 wells in each group. |
| S1h | **Simple linear regression test**  Aβ(-)(*F*=25.52, *p*<0.001, *R^2^*=0.114), Aβ(+)(*F*=59.34, *p*<0.001, *R^2^*=0.230)  A total of 200 cell counts, 50 cells/well, *n* = 4 wells in each group. |
| S2d | **LC3B-II/ACTB**  1 hr (1.603 ± 0.026), 2 hr (1.709 ± 0.108), 4 hr (1.168 ± 0.004), 8 hr (0.949 ± 0.135), 12 hr (0.897 ± 0.192), 24 hr (0.979 ± 0.209), 48 hr (0.979 ± 0.209), 72 hr (1.098 ± 0.260), 96 hr (1.016 ± 0.215), 120 hr (0.797 ± 0.104)  Unpaired two-tailed *t*-test; 0 hr *vs* 1hr, *p*<0.001; 0 hr *vs* 2 hr, *p*=0.031, 0 hr *vs* 4hr, *p*<0.001.  **SQSTM1/ACTB**  1 hr (1.338 ± 0.025), 2 hr (1.813 ± 0.162), 4 hr (2.065 ± 0.472), 8 hr (2.061 ± 0.343), 12 hr (1.195 ± 0.003), 24 hr (0.990 ± 0.101), 48 hr (0.913 ± 0.140), 72 hr (0.698 ± 0.220), 96 hr (0.370 ± 0.097), 120 hr (0.396 ± 0.095)  All data were generated from 2 separate experiment, triplicate/experiment.  Normalized to the average of ACTB level.  Unpaired two-tailed *t*-test; 0 hr *vs* 1 hr, *p*=0.005; 0 hr *vs* 2 hr, *p*=0.037, 0 hr *vs* 12 hr, *p*<0.001; 0 hr *vs* 96 hr, *p*=0.023; 0 hr *vs* 120 hr, *p*=0.023. |
| S3c | **CA1**  Normal (1 ± 0.035), NPCAD (1.349 ± 0.055), SAD (2.622 ± 0.0.93)  One-way ANOVA with Tukey’s multiple comparisons test; *F*=166.5, *p<*0.001.  Normal *vs* NPCAD, *p<*0.001; Normal *vs* SAD, *p*<0.001; NPCAD *vs* SAD, *p<*0.001.  **CA2**  Normal (1 ± 0.036), NPCAD (1.738 ± 0.061), SAD (2.331 ± 0.060)  One-way ANOVA with Tukey’s multiple comparisons test; *F*=154.3, *p<*0.001.  Normal *vs* NPCAD, *p<*0.001; Normal *vs* SAD, *p*<0.001; NPCAD *vs* SAD, *p<*0.001.  **CA3**  Normal (1 ± 0.043), NPCAD (1.924 ± 0.075), SAD (3.239 ± 0.093)  One-way ANOVA with Tukey’s multiple comparisons test; *F*=231.6, *p<*0.001.  Normal *vs* NPCAD, *p<*0.001; Normal *vs* SAD, *p*<0.001; NPCAD *vs* SAD, *p<*0.001.  **CA4**  Normal (1 ± 0.052), NPCAD (1.849 ± 0.048), SAD (3.020 ± 0.084)  One-way ANOVA with Tukey’s multiple comparisons test; *F*=269.7, *p<*0.001.  Normal *vs* NPCAD, *p<*0.001; Normal *vs* SAD, *p*<0.001; NPCAD *vs* SAD, *p<*0.001.  **DG**  Normal (1 ± 0.050), NPCAD (2.118 ± 0.060), SAD (3.312 ± 0.086)  One-way ANOVA with Tukey’s multiple comparisons test; *F*=292.5, *p<*0.001.  Normal *vs* NPCAD, *p<*0.001; Normal *vs* SAD, *p*<0.001; NPCAD *vs* SAD, *p<*0.001.  **Ent Cx**  Normal (1 ± 0.040), NPCAD (1.533 ± 0.044), SAD (2.368 ± 0.072)  One-way ANOVA with Tukey’s multiple comparisons test; *F*=160.9, *p<*0.001.  Normal *vs* NPCAD, *p<*0.001; Normal *vs* SAD, *p*<0.001; NPCAD *vs* SAD, *p<*0.001.  A total of 60 cell counts, 12 cells/case, *N* = 5 cases in each group.  Normalized to the average of normal. |
| S6b | **LC3B**  Control (45.51 ± 3.114), E/P (60.91 ± 3.474), CQ (52.18 ± 2.886), Aβ oligomer (89.71 ± 6.769), Aβ oligomer + E/P (145.4 ± 11.46), Aβ oligomer + CQ (136.6 ± 9.340)  One-way ANOVA with Tukey’s multiple comparisons test; *F*=38.64, *p<*0.001.  Control *vs* Aβ oligomer, *p*<0.001; Control *vs* Aβ oligomer + E/P, *p*<0.001; Control *vs* Aβ oligomer + CQ, *p*<0.001; Aβ oligomer *vs* Aβ oligomer + E/P, *p*<0.001; Aβ oligomer *vs* Aβ oligomer + CQ, *p*<0.001.  A total of 20 cell counts was examined from *n* = 4 wells in each group. |
| S6c | **SQSTM1**  Control (44.79 ± 3.475), E/P (44.87 ± 5.244), CQ (63.81 ± 4.836), Aβ oligomer (93.99 ± 6.608), Aβ oligomer + E/P (158.6 ± 10.43), Aβ oligomer + CQ (142.8 ± 8.252)  One-way ANOVA with Tukey’s multiple comparisons test; *F*=50.38, *p<*0.001.  Control *vs* Aβ oligomer, *p*<0.001; Control *vs* Aβ oligomer + E/P, *p*<0.001; Control *vs* Aβ oligomer + CQ, *p*<0.001; Aβ oligomer *vs* Aβ oligomer + E/P, *p*<0.001; Aβ oligomer *vs* Aβ oligomer + CQ, *p*<0.001.  A total of 20 cell counts was examined from *n* = 4 wells in each group. |
| S8b | Control (1 ± 0.165, *n* = 6 wells), LC3B shRNA (1.022 ± 0.085, *n* = 6 wells), Aβ oligomer (2.406 ± 0.251, *n* = 7 wells), Aβ oligomer + LC3B shRNA (0.831 ± 0.100, *n* = 6 wells)  Normalized to the average of control.  One-way ANOVA with Tukey’s multiple comparisons test; *F*=18.87, *p*<0.001.  Control *vs* Aβ oligomer, *p*<0.001; Aβ oligomer *vs* Aβ oligomer + LC3B shRNA, *p*<0.001; LC3B shRNA *vs* Aβ oligomer, *p*<0.001. |
| S8c | Control (1 ± 0.141, *n* = 6 wells), LC3B shRNA (0.885 ± 0.027, *n* = 6 wells), Aβ oligomer (1.153 ± 0.121, *n* = 7 wells), Aβ oligomer + LC3B shRNA (1.047 ± 0.061, *n* = 6 wells)  Normalized to the average of control.  One-way ANOVA with Tukey’s multiple comparisons test; *F*=0.490, *p=*0.621. |
| S8d | Control (1 ± 0.078, *n* = 6 wells), LC3B shRNA (0.813 ± 0.078, *n* = 6 wells), Aβ-oligomer (1.196 ± 0.100, *n* = 7 wells), Aβ-oligomer + LC3B shRNA (0.722 ± 0.049, *n* = 6 wells)  Normalized to the average of control.  One-way ANOVA with Tukey’s multiple comparisons test; *F*=6.988, *p*=0.0019.  LC3B shRNA *vs* Aβ oligomer, *p=*0.013; Aβ oligomer *vs* Aβ oligomer + LC3B shRNA, *p*=0.002. |
| S8e | Control (1 ± 0.077, *n* = 6 wells), LC3B shRNA (1.100 ± 0.088, *n* = 6 wells), Aβ oligomer (1.712 ± 0.222, *n* = 7 wells), Aβ oligomer + LC3B shRNA (0.542 ± 0.057, *n* = 6 wells)  Normalized to the average of control.  One-way ANOVA with Tukey’s multiple comparisons test; *F*=18.39, *p*<0.001.  Control *vs* Aβ oligomer, *p=*0.007; Aβ oligomer *vs* Aβ oligomer + LC3B shRNA, *p*<0.001; LC3B shRNA *vs* Aβ oligomer, *p=*0.023. |
| S8f | Control (1 ± 0.106, *n* = 6 wells), LC3B shRNA (1.336 ± 0.207, *n* = 6 wells), Aβ oligomer (1.662 ± 0.194, *n* = 7 wells), Aβ oligomer + LC3B shRNA (0.491 ± 0.029, *n* = 6 wells)  Normalized to the average of control.  One-way ANOVA with Tukey’s multiple comparisons test; *F*=10.29, *p*<0.001.  Control *vs* Aβ oligomer, *p=*0.030; Aβ oligomer *vs* Aβ oligomer + LC3B shRNA, *p*<0.001; LC3B shRNA *vs* Aβ oligomer + LC3B shRNA, *p=*0.006. |
| S8g | Control (1 ± 0.067, *n* = 6 wells), LC3B shRNA (1.066 ± 0.066, *n* = 6 wells), Aβ oligomer (1.707 ± 0.206, *n* = 7 wells), Aβ oligomer + LC3B shRNA (0.507 ± 0.034, *n* = 6 wells)  Normalized to the average of control.  One-way ANOVA with Tukey’s multiple comparisons test; *F*=15.92, *p*<0.001.  Control *vs* Aβ oligomer, *p=*0.003; Aβ oligomer *vs* Aβ oligomer + LC3B shRNA, *p*<0.001; LC3B shRNA *vs* Aβ oligomer, *p=*0.007. |
| S9a | Control (1.015 ± 0.043), Aβ oligomer (1.181 ± 0.134), LC3B shRNA (0.620 ± 0.036), Aβ oligomer + LC3B shRNA (0.555 ± 0.018)  A total of 4 experiment, triple replications/experiment.  Normalized to the average of control.  One-way ANOVA with Tukey’s multiple comparisons test; *F* = 9.638, *p* < 0.001.  Control *vs* LC3B shRNA, *p*=0.012; Control *vs* Aβ oligomer + LC3B shRNA, *p*=0.004. |
| S9b | Control (1 ± 0.012), LC3B shRNA#1 (0.691 ± 0.043), LC3B shRNA#2 (0.100 ± 0.031)  A total of 3 experiment, triple replications/experiment.  Normalized to the average of ACTB level.  One-way ANOVA with Tukey’s multiple comparisons test; *F*=205.4, *p*<0.001.  Control *vs* LC3B shRNA#2, *p<*0.001; LC3B shRNA#1 *vs* LC3B shRNA#2, *p*=0.001. |
| S9d | Control (1 ± 0.016), Aβ oligomer (0.973 ± 0.051), LC3B shRNA (0.908 ± 0.113), Aβ oligomer + LC3B shRNA (1.008 ± 0.081)  A total of 4 experiment, triple replications/experiment.  Normalized to the average of control.  One-way ANOVA with Tukey’s multiple comparisons test; *F*=0.425, *p*=0.738. |
| S9e | Control (1 ± 0.015), SQSTM1 shRNA (0.232 ± 0.068)  A total of 4 experiment, triple replications/experiment.  Normalized to the average of control.  Unpaired two-tailed *t*-test; *p<*0.001. |
| S9f | Control (1 ± 0.061), LC3B shRNA (0.912 ± 0.059), SQSTM1 shRNA (0.198 ± 0.044)  A total of 3 experiment, triple replications/experiment.  Normalized to the average of control.  One-way ANOVA with Tukey’s multiple comparisons test, *F*=61.86, *p*<0.001.  Control *vs* SQSTM1 shRNA, *p<*0.001; LC3B shRNA *vs* SQSTM1 shRNA, *p<*0.001. |
| S9h | Control + APP/PS1 (82.03 ± 5.614 %), LC3B shRNA + APP/PS1 (70.69 ± 3.199 %), SQSTM1 shRNA + APP/PS1 (78.24 ± 4.289 %)  A total of 5 ROIs (mm^2^) was examined from *N* = 3 mice in each group.  One-way ANOVA with Tukey’s multiple comparisons test; *F*=1.663, *p*=0.230. |
| S10d | WT + Control (10.55 ± 4.899), APP/PS1 + Control (610.8 ± 71.47), APP/PS1 + Casp3 (958.7 ± 105.2)  A total of 20 ROIs (2500 μm^2^) was examined from *N* = 4 mice in each group.  One-way ANOVA with Tukey’s multiple comparisons test; *F*=42.58, *p*<0.001.  WT + Control *vs* APP/PS1 + Control, *p*<0.001; APP/PS1 + Control *vs* APP/PS1 + Casp3, *p=*0.004; WT + Control *vs* APP/PS1 + Casp3, *p*<0.001. |
| S10e | WT + Control (0.045 ± 0.006 μm^2^), APP/PS1 + Control (0.196 ± 0.043 μm^2^), APP/PS1 + Casp3 (1.241 ± 0.176 μm^2^)  A total of 20 ROIs (2500μm^2^) was examined from *N* = 4 mice in each group.  One-way ANOVA with Tukey’s multiple comparisons test; *F*=38.61, *p*<0.001.  WT + Control *vs* APP/PS1 + Control, *p=*0.001; APP/PS1 + Control *vs* APP/PS1 + Casp3, *p*<0.001; WT + Control *vs* APP/PS1 + Casp3, *p*<0.001. |
| S11b | WT + Control (177.5 ± 11.15 μm), APP/PS1 + Control (301.1 ± 13.20 μm), APP/PS1 + LC3B shRNA (513.5 ± 28.73 μm), APP/PS1 + SQSTM1 shRNA (604.8 ± 29.89 μm)  A total of 20 cell counts was examined from *N* = 4 mice in each group.  One-way ANOVA with Tukey’s multiple comparisons test; *F*=75.42, *p*<0.001.  WT + Control *vs* APP/PS1 + Control, *p*=0.001; WT + Control *vs* APP/PS1 + LC3B shRNA, *p*<0.001; WT + Control *vs* APP/PS1 + SQSTM1 shRNA, *p*<0.001; APP/PS1 + Control *vs* APP/PS1 + LC3B shRNA, *p*=0.010; APP/PS1 + Control *vs* APP/PS1 + SQSTM1 shRNA, *p<*0.001; APP/PS1 + LC3B shRNA *vs* APP/PS1 + SQSTM1 shRNA, *p*=0.026. |
| S11c | WT + Control (4.015 ± 0.836 μm, 8 cells), APP/PS1 + Control (4.651 ± 0.940 μm, 12 cells), APP/PS1 + LC3B shRNA (7.692 ± 1.555 μm, 13 cells), APP/PS1 + SQSTM1 shRNA (6.793 ± 1.452 μm, 13 cells)  Mixed-effect analysis; *F*=33.38, *p*<0.001.  APP/PS1 + Control *vs* APP/PS1 + LC3B shRNA, *p*=0.010; APP/PS1 + Control *vs* APP/PS1 + LC3B shRNA, *p*=0.016. |
| S11e | WT + Control (203.8 ± 5.365 cells), APP/PS1 + Control (160.0 ± 8.375 cells), APP/PS1 + LC3B shRNA (143.5 ± 5.836 cells), APP/PS1 + SQSTM1 shRNA (136.5 ± 8.218 cells)  A total of 10 ROIs (0.25 mm^2^) was examined from *N* = 4 mice in each group.  One-way ANOVA with Tukey’s multiple comparisons test; *F*=22.25, *p*<0.001.  WT + Control *vs* APP/PS1 + Control, *p*<0.001; APP/PS1 + Control *vs* APP/PS1 + LC3B shRNA, *p=*0.038; APP/PS1 + Control *vs* APP/PS1 + SQSTM1 shRNA, *p=*0.017. |
| S13a | **LC3B**  Control (1 ± 0.350), LC3B OE (364388 ± 127210)  Unpaired two-tailed *t*-test; *p=*0.045.  **SQSTM1**  Control (1 ± 0.046), LC3B OE (0.964 ± 0.196)  Unpaired two-tailed *t*-test, *p=*0.868.  A total of 3 experiments, triple replications/experiment.  Normalized to the average of control. |
| S13c | Control (18.13 ± 1.826), LC3B OE (89.37 ± 5.033)  A total of 100 cell counts, 25 cells/well, *n* = 4 wells in each group.  Normalization to average of control.  Unpaired two-tailed *t*-test, *p<*0.001. |
| S13d | Control (1 ± 0.091), Aβ oligomer (1.927 ± 0.174), LC3B OE + Aβ oligomer (1.30 ± 0.034)  A total of 30 cell counts, 10 cells/well, *n* = 3 wells in each group.  Normalization to average of control.  One-way ANOVA with Tukey’s multiple comparisons test; *F*=16.79, *p=*0.003.  Control *vs* Aβ oligomer, *p=*0.003; Aβ oligomer *vs* LC3B OE + Aβ oligomer, *p=*0.02. |
| S13e | Control (1 ± 0.085), Aβ oligomer (1.872 ± 0.234), LC3B OE + Aβ oligomer (1.112 ± 0.021)  A total of 30 cell counts, 10 cells/well, *n* = 3 wells in each group.  Normalization to average of control.  One-way ANOVA with Tukey’s multiple comparisons test; *F*=10.78, *p=*0.01.  Control *vs* Aβ oligomer, *p=*0.0125; Aβ oligomer *vs* LC3B OE + Aβ oligomer, *p=*0.023. |
| S13f | WT + Control (82.45 ± 3.24 %), APP/PS1 + Control (81.98 ± 2.87 %), APP/PS1 + LC3B OE (89.32 ± 2.25%)  A total of 9 ROIs (0.25 mm^2^) was examined from *N* = 3 mice in each group.  One-way ANOVA with Tukey’s multiple comparisons test; *F*= 0.128, *p*=0.141. |

**Supplementary Table 3**. Information on the postmortem brain tissues used in this study. The pathological stage for AD was classified according to Braak stage as previously described (Braak H & Braak E,1991). Control, not diagnosed with AD; NPCAD, Neuropathologically and Clinically Diagnosed Alzheimer’s Disease; SAD, Severe Alzheimer’s Disease, CERAD.

| **No.** | **Neuropathological**  **Diagnosis** | **Sex** | **Age** | **Braak Stage** |
| --- | --- | --- | --- | --- |
| 1 | Control | F | 92 | I |
| 2 | Control | F | 87 | I |
| 3 | Control | F | 83 | II |
| 4 | Control | M | 94 | II |
| 5 | Control | F | 94 | III |
| 6 | Control | M | 82 | I |
| 7 | Control | M | 70 | I |
| 8 | Control | F | 78 | I |
| 9 | Control | F | 79 | I |
| 10 | Control | M | 92 | I |
| 11 | Control | F | 81 | I |
| 12 | NPCAD | M | 92 | II |
| 13 | NPCAD | M | 82 | IV |
| 14 | NPCAD | F | 88 | II |
| 15 | NPCAD | F | 93 | III |
| 16 | NPCAD | F | 72 | III |
| 17 | NPCAD | F | 83 | III |
| 18 | NPCAD | F | 95 | II |
| 19 | NPCAD | M | 87 | III |
| 20 | NPCAD | F | 82 | III |
| 21 | NPCAD | M | 92 | II |
| 22 | NPCAD | F | 93 | III |
| 23 | SAD | M | 90 | VI |
| 24 | SAD | M | 80 | V |
| 25 | SAD | F | 87 | VI |
| 26 | SAD | M | 77 | VI |
| 27 | SAD | F | 80 | VI |
| 28 | SAD | F | 92 | VI |
| 29 | SAD | M | 89 | IV |
| 30 | SAD | F | 93 | IV |
| 31 | SAD | M | 83 | V |
| 32 | SAD | F | 93 | VI |

Reference:

Braak, H. and Braak, E. (1991). Neuropathological stageing of Alzheimer-related changes. *Acta Neuropathologica*. **82** (4): 239–59. [doi](https://en.wikipedia.org/wiki/Doi_(identifier)):[10.1007/BF00308809](https://doi.org/10.1007%2FBF00308809).

**Supplementary Figures**

**
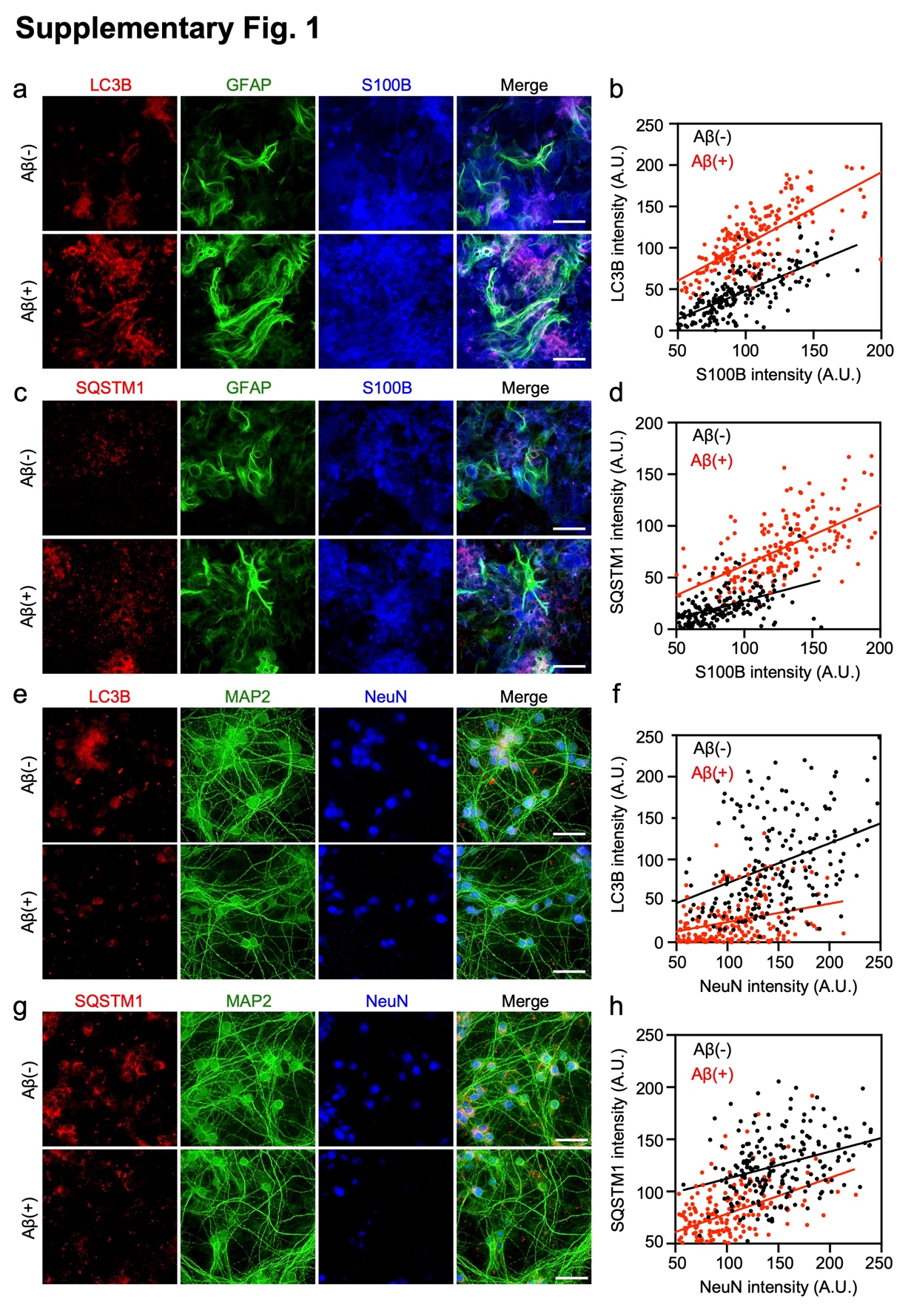
**

**Supplementary Fig. 1 Autophagy components are differentially expressed between astrocytes and neurons in response to Aβ oligomer in the primary coculture. a,** Representative images of triple immunofluorescence staining for LC3B (red), GFAP (green), and S100B (blue) in primary mouse astrocyte and neurons co-culture system. Scale bars (white): 20 μm. **b,** Correlation analysis of S100B and LC3B immunoreactivity in response to Aβ oligomer treatment. A total of 200 cell counts, 50 cells/well, *n* = 4 wells. **c,** Representative images of triple immunofluorescence staining for SQSTM1 (red), GFAP (green), and S100B (blue) in primary mouse astrocyte and neurons co-culture system. Scale bars (white): 20 μm. **d,** Correlation analysis of S100B and SQSTM immunoreactivity in response to Aβ oligomer treatment. A total of 200 cell counts, 50 cells/well, *n* = 4 wells. **e,** Representative images of triple immunofluorescence staining for LC3B (red), MAP2 (green), and NeuN (blue) in primary mouse astrocyte and neurons co-culture system. Scale bars (white): 20 μm. **f,** Correlation analysis of NeuN and LC3B immunoreactivity in response to Aβ oligomer treatment. A total of 200 cell counts, 50 cells/well, *n* = 4 wells. **g,** Representative images of triple immunofluorescence staining for SQSTM1 (red), MAP2 (green), and NeuN (blue) in primary mouse astrocyte and neuron co-culture system. Scale bars (white): 20 μm. **h,** Correlation analysis of NeuN and SQSTM1 immunoreactivity in response to Aβ oligomer treatment. A total of 200 cell counts, 50 cells/well, *n* = 4 wells. Significantly different at *, *p*<0.05; **, *p*<0.01.


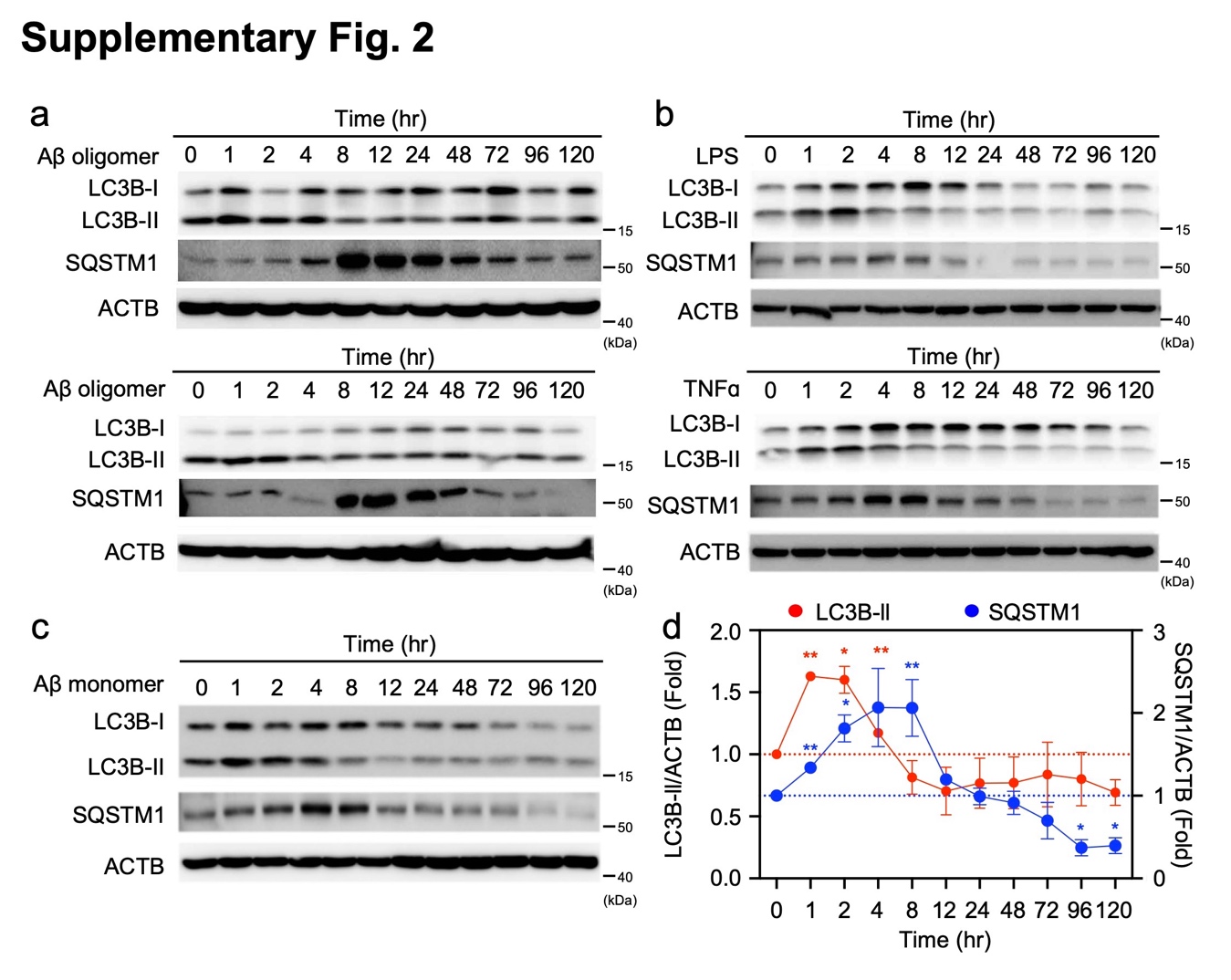


**Supplementary Fig. 2 Autophagy components are increased in primary mouse astrocytes in response to pro-inflammatory factors (LPS or TNFα), Aβ monomer, and Aβ oligomer. a,** Western blot showing that levels of endogenous SQSTM1 and LC3B-II are time-dependently increased in Aβ oligomer-treated mouse astrocytes. **b,**Western blot showing that levels of endogenous SQSTM1 and LC3B-II are time-dependently increased in LPS- or TNFα- treated mouse astrocytes. **c,** Western blot showing that levels of endogenous SQSTM1 and LC3B-II are time-dependently increased in Aβ monomer-treated mouse astrocytes. **d,** Quantification of the band intensities of LC3B-II and SQSTM1 normalized by ACTB. Data are presented as mean ± SEM from three separate experiments. Significantly different at *, p<0.05; **, p<0.01.


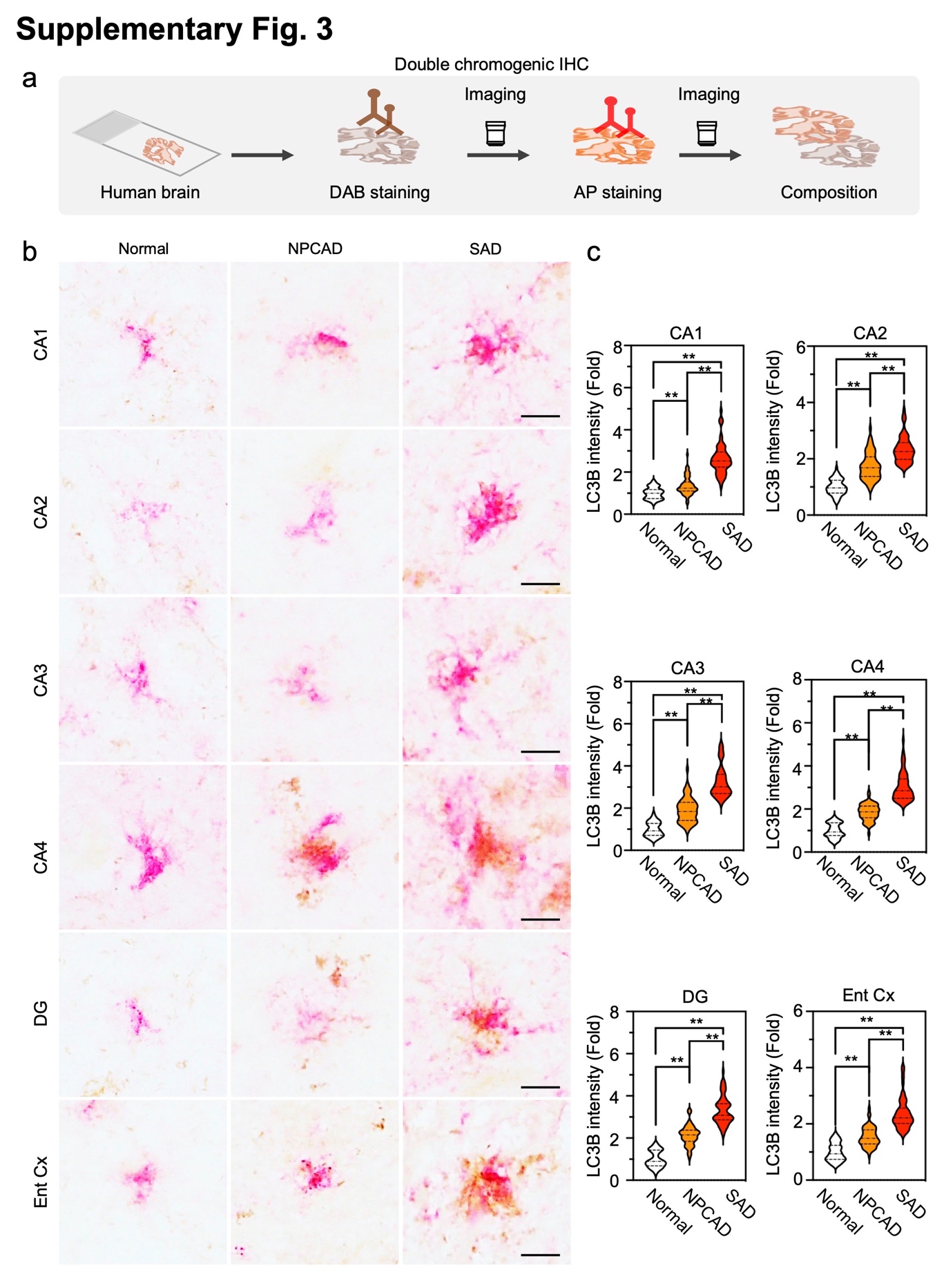


**Supplementary Fig. 3 Autophagy component (LC3B) is upregulated in the astrocyte of AD postmortem brains. a,** Immunostaining scheme for human control and AD postmortem brain tissue sections. **b**, Immunostaining for LC3B (brown) and GFAP (red) in CA1, CA2, CA3, CA4 and DG of hippocampus and entorhinal cortex (Ent Cx) in normal, NPCAD, and SAD patients. Scale bars: 10 μm. **c,** Quantification of average intensity of LC3B in CA1, CA2, CA3, CA4 and DG of hippocampus and Ent Cx in normal, NPCAD, and SAD patients. A total of 60 cell counts, 12 cells/case from N = 5 cases in each group. Data are presented as mean ± SEM. Significantly different at *, p<0.05; **, p<0.01.


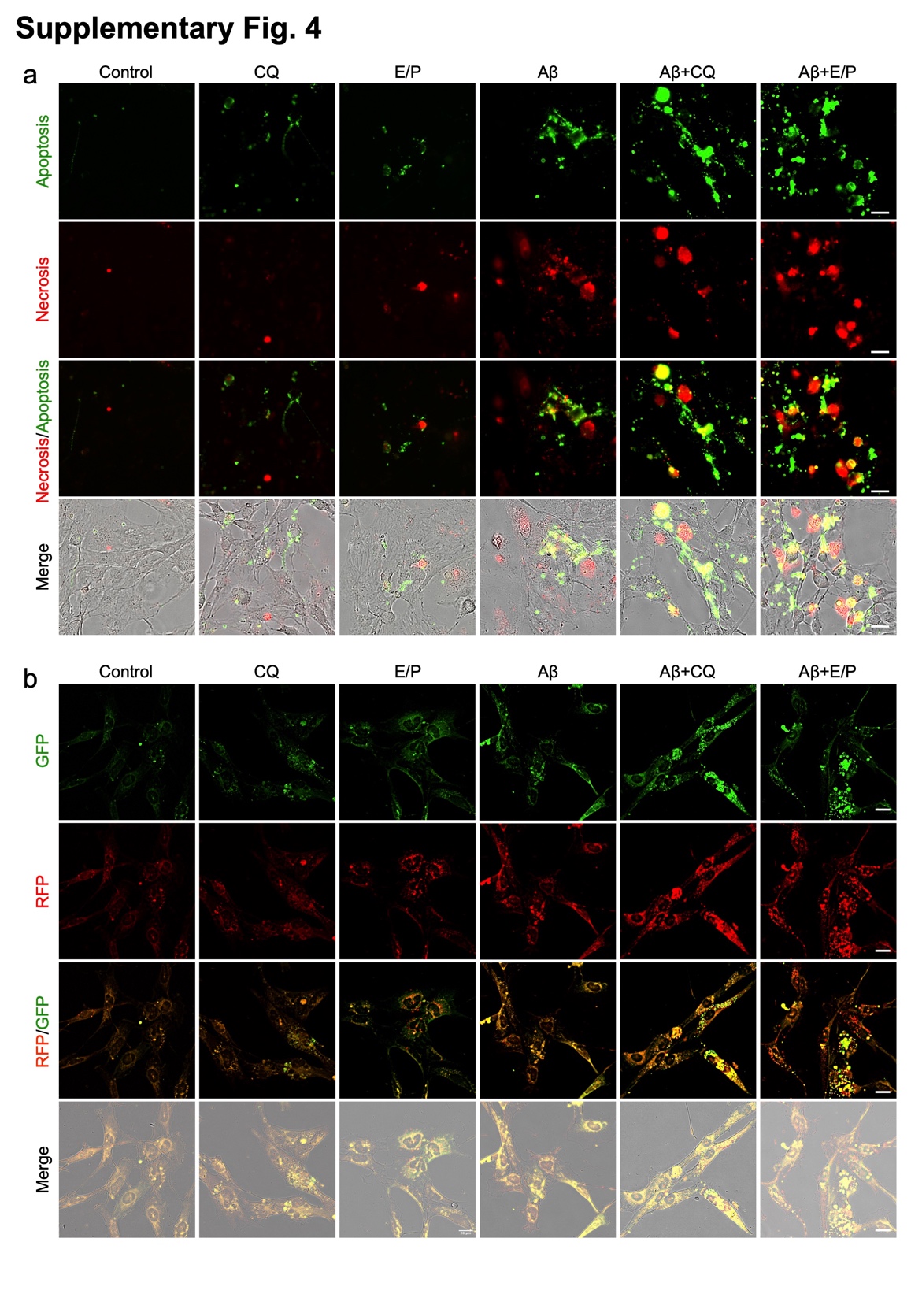


**Supplementary Fig. 4 Representative images for the patterns of astrocytic cell death with or without autophagy inhibitors in vitro. a,** Low magnification view of astrocyte morphology, apoptotic cell death signals (green), and necrotic cell death signals (red) in autophagy inhibitor- and/or Aβ oligomer-treated human astrocytes. Treatment conditions: Aβ oligomer, 1 μM; CQ (Chloroquine), 20 μM; E/P (E64D/ Pepstatin A), 10 μg/ml; time, 24 hr. Scale bars (white): 20 μm.  **b,**Low magnification view of RFP-GFP-LC3B-expressing astrocytes with or without autophagy inhibitors (CQ or E/P) in Aβ oligomer-treated human astrocytes. Green dots indicate autophagosomes and red dots indicate autolysosomes. Scale bars (white): 20 μm.


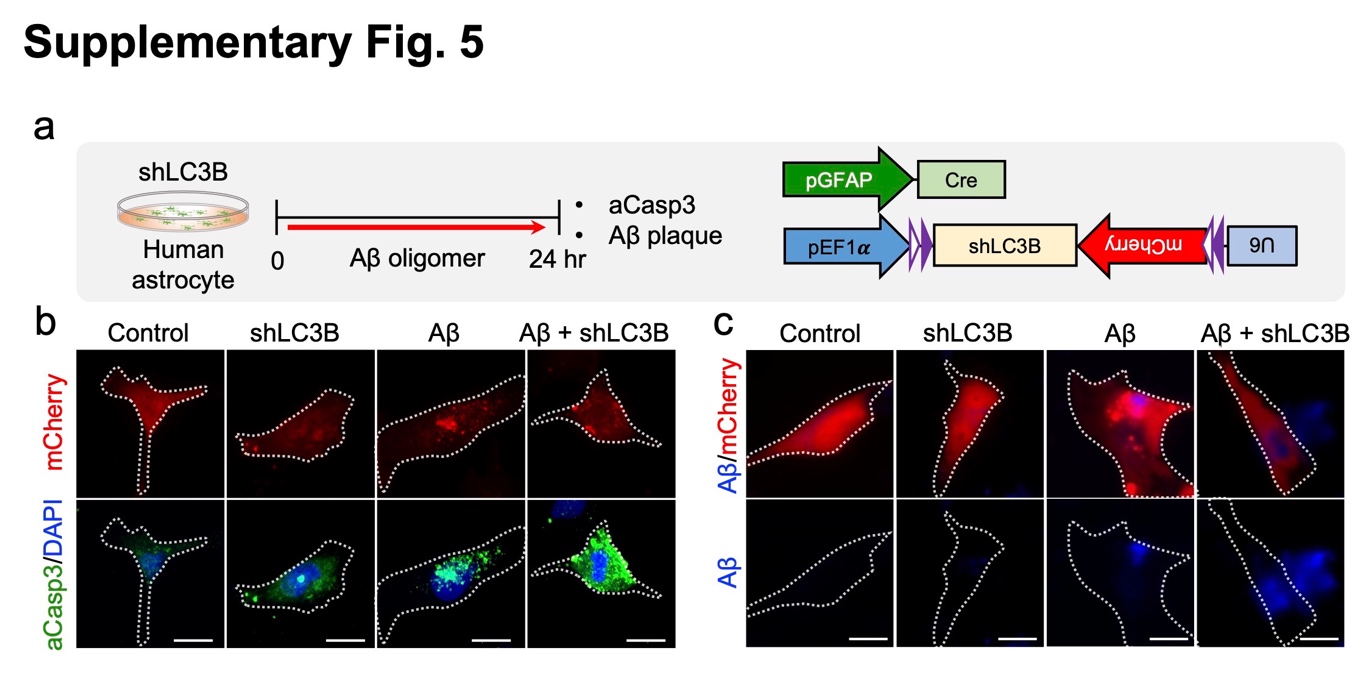


**Supplementary Fig. 5 Validation of shRNAs (LC3B and SQSTM1) in astrocytes. a**, Experimental timeline for AAV infection, active caspase-3 staining, and Aβ oligomer treatment in LC3B shRNA-expressed cultured astrocytes. **b & c,** Representative images of active caspase-3 (green) (**b**), and Aβ oligomer-uptake (blue) (**c**) in LC3B shRNA-expressed human astrocytes. Scale bars (white): 10 μm.


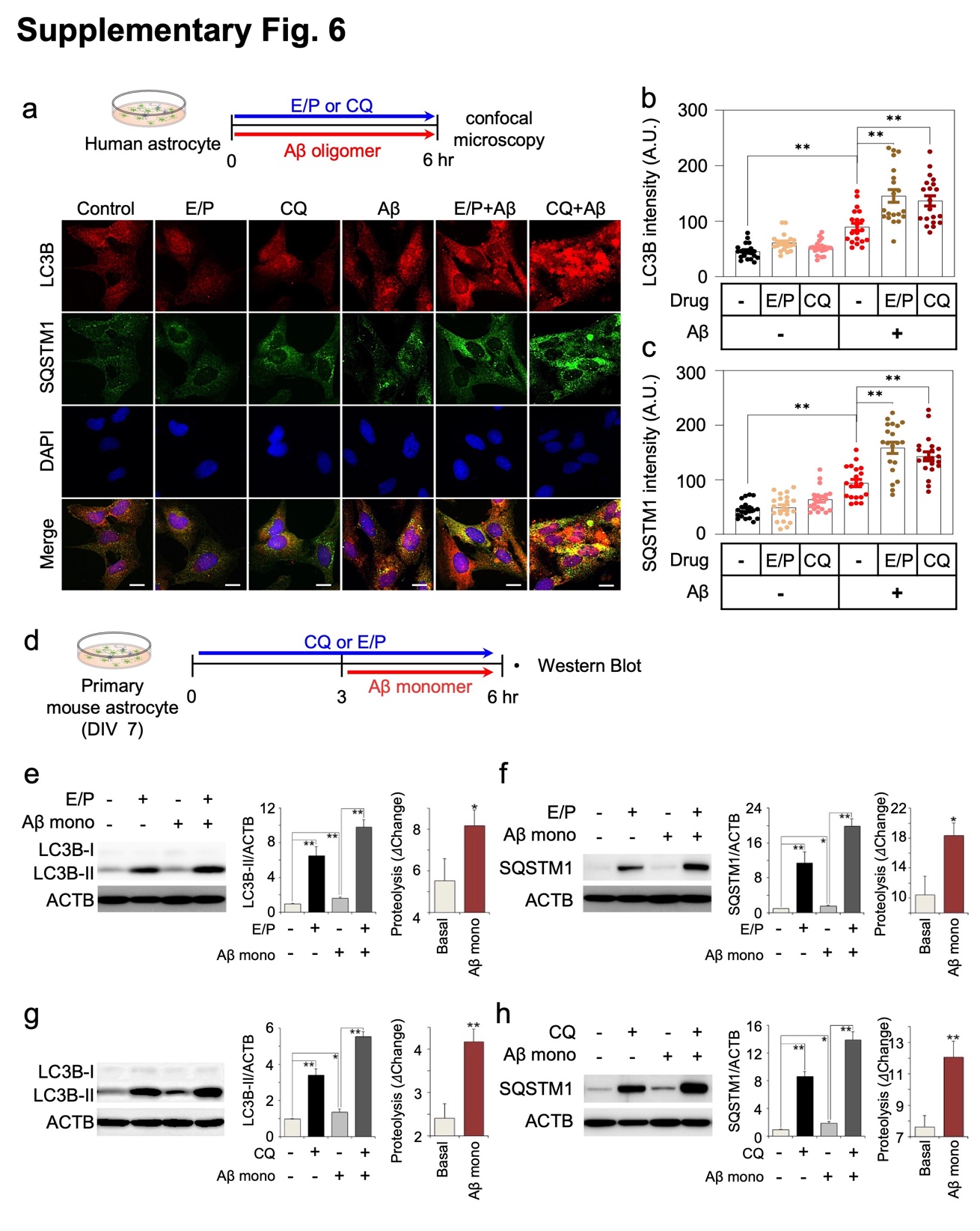


**Supplementary Fig. 6 Autophagy flux is elevated in response to Aβ oligomer in both human astrocytes and primary mouse astrocytes. a,** Experimental design for autophagy flux assay in oligomeric Aβ-treated human astrocytes. **b & c,** Autophagic flux assay and densitometry analysis in Aβ oligomer-treated human astrocytes. A total of 20 cell counts, 5 cells/well, n = 4 wells in each group. Treatment condition: Aβ, 1 µg/ml; E/P, E64D (10 µg/ml) + Pepstatin A (10 µg/ml); CQ, 20 µM; time, 6 hr. **d,** Experimental timeline of Western blots in primary mouse astrocytes. **e-h,** Western blot showing that levels of endogenous SQSTM1 and LC3B-II levels with or without autophagy in Aβ oligomer-treated primary astrocytes. Quantification for the intensity of LC3B (**e & g**) and SQSTM1 (**f & h**) protein signals. Bar graphs represent mean ± SEM from three separate experiments. Significantly different at *, p<0.05; **, p<0.01.


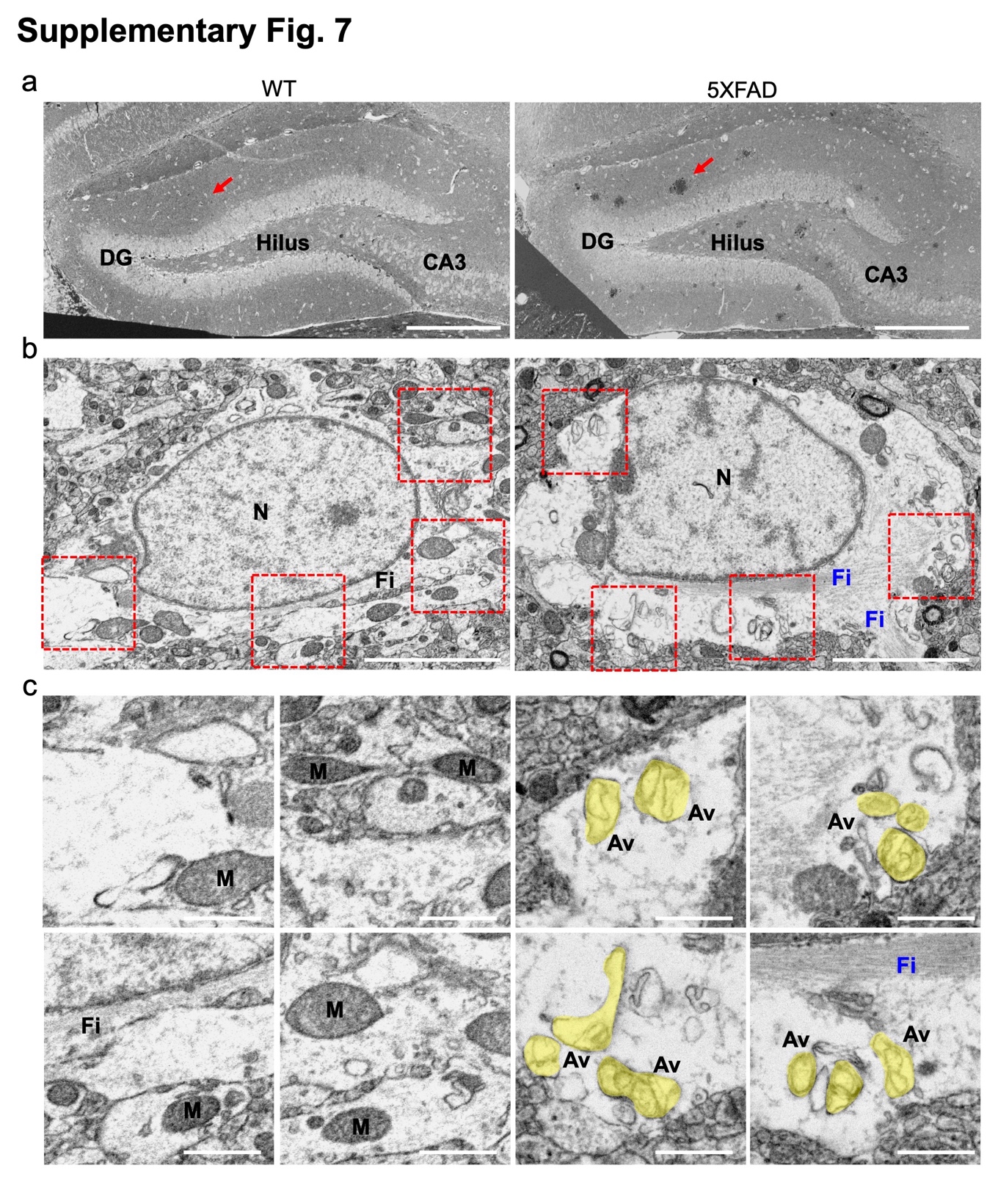


**Supplementary Fig. 7 EM images representing an increase in autophagic vesicles (double membrane vesicles) in astrocytes of 5xFAD mouse.** **a**, Low magnification views of the hippocampus in WT and 5xFAD mouse. An arrow (red) in the DG molecular layer of 5xFAD mouse indicates a ROI at the vicinity of Aβ plaque while an arrow (red) in WT mouse indicates the same region as matched to the ROI of 5xFAD. **b**, Representative image of the hippocampal astrocyte in WT and 5xFAD mouse. **c**, Zoom-in views of subcellular compartments (Av, autophagic vesicles; Fi, filament; M, mitochondria; N, nucleus) in the hippocampal astrocyte of WT and 5xFAD mouse. The number of Avs (double-membrane structure) (yellow) are increased in the hippocampal astrocyte of 5xFAD mouse. Scale bars (white): panel a, 0.5 mm; panel b, 5 μm; panel c, 100 μm.


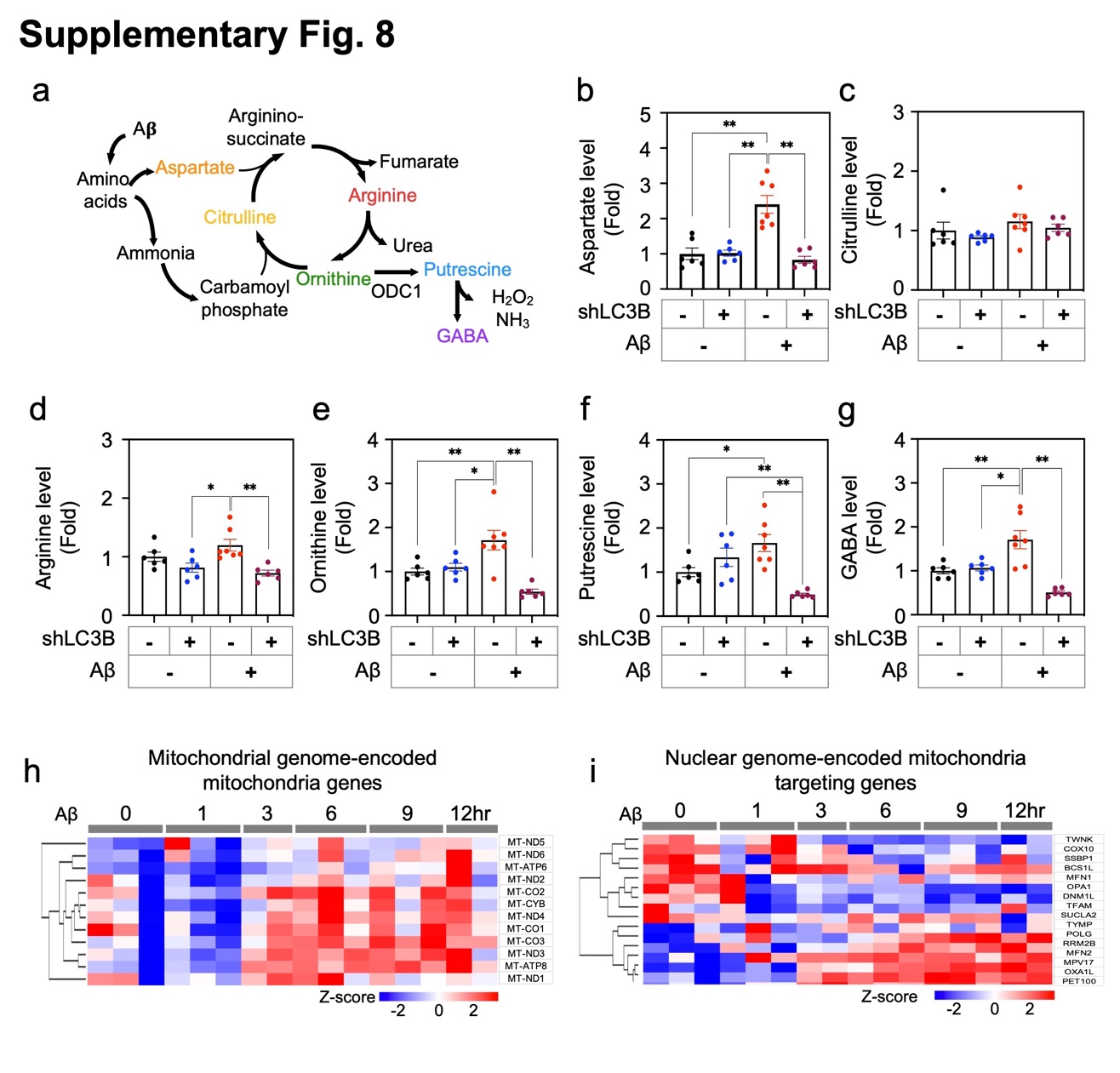


**Supplementary Fig. 8 Level of urea cycle-related metabolites and the expression of mitochondria targeting genes are altered in response to Aβ oligomer treatment in astrocytes. a,** Schematic diagram of Aβ oligomer-induced urea cycle. **b-g,** Bar graphs showing relative levels of metabolites including aspartate (**b**), citrulline (**c**), arginine (**d**), ornithine (**e**), putrescine (**f**), and GABA (**g**) in AAV-LC3B shRNA-infected primary mouse astrocyte. Data are presented as mean ± SEM. Significantly different at *, p<0.05; **, p<0.01. **h & i,** Heatmap analyses for mitochondrial genome-encoded mitochondria genes (**h**), and nuclear genome-encoded mitochondria targeting genes (**i**) in RNA-sequencing data from human astrocytes at 1, 3, 6, 9, and 12 hr after Aβ oligomer treatment.


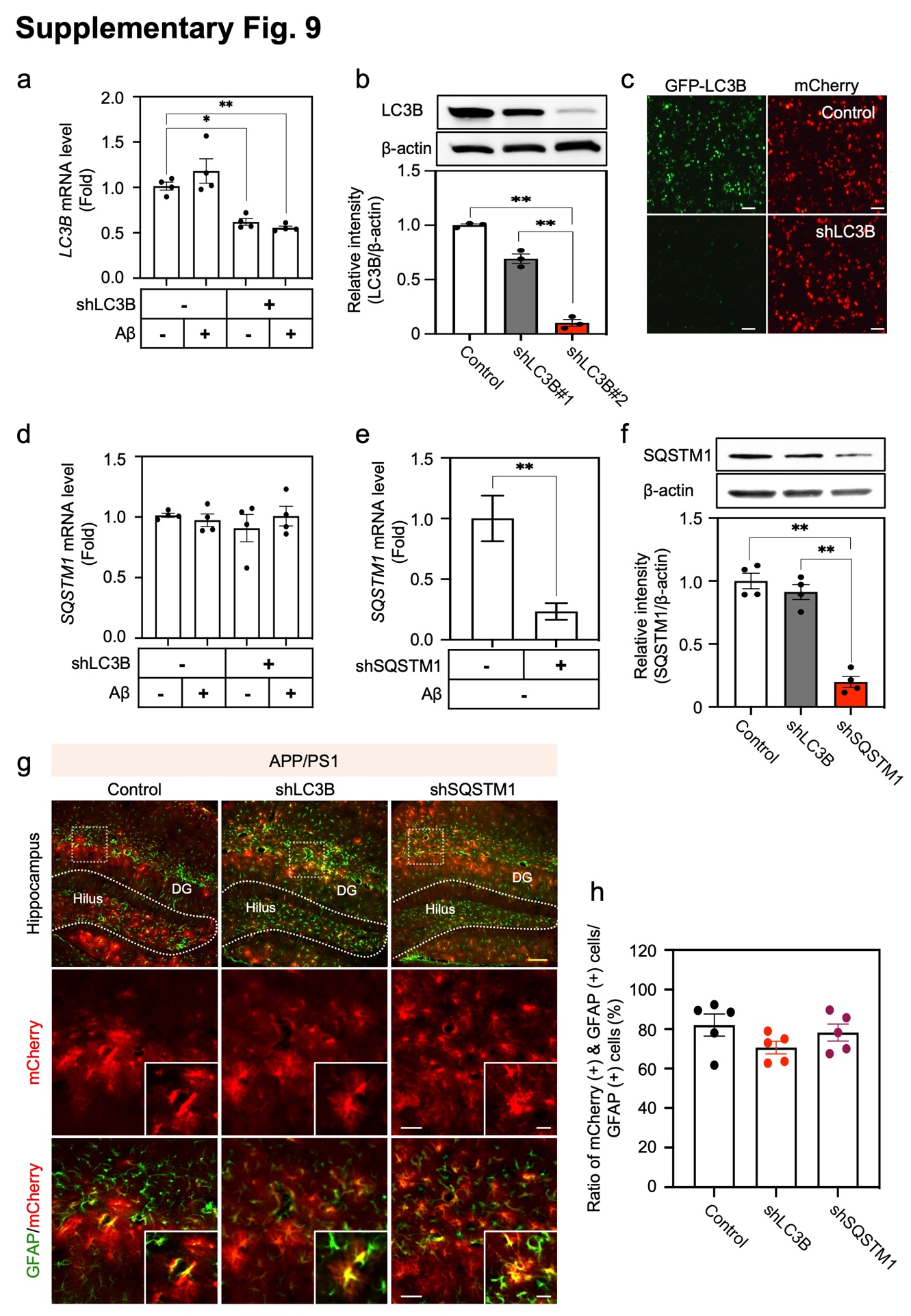


**Supplementary Fig. 9 Validation of shRNAs-mediated down regulation of LC3B and SQSTM1. a-c,** The knockdown efficiency of LC3B shRNA was verified by qRT-PCR (**a**), Western blot analysis (**b**), and immunocytochemistry (**c**). Scale bars (white): 20 μm. **d**, LC3B shRNA did not affect the expression of *SQSTM1*. **e & f,** Knockdown efficiency of SQSTM1 shRNA was verified by qRT-PCR (**e**), and Western blot analysis (**f**)**. g**, Astrocyte-specific knockdown of *LC3B* and SQSTM1 was verified by immunostaining in APP/PS1 mice. A total of 5 ROIs (mm^2^) from *N* = 3 mice in each group. Scale bars:100 μm (top), 20 μm (2^nd^ & 3^rd^ panel). Red color is shown to AAV virus infected cells. **h**, The infection efficiency of AAV-LC3B shRNA and SQSTM1 shRNA in astrocytes was found at 70 to 80% levels. Data are presented as mean ± SEM. Significantly different at *, *p*<0.05; **, *p*<0.01.


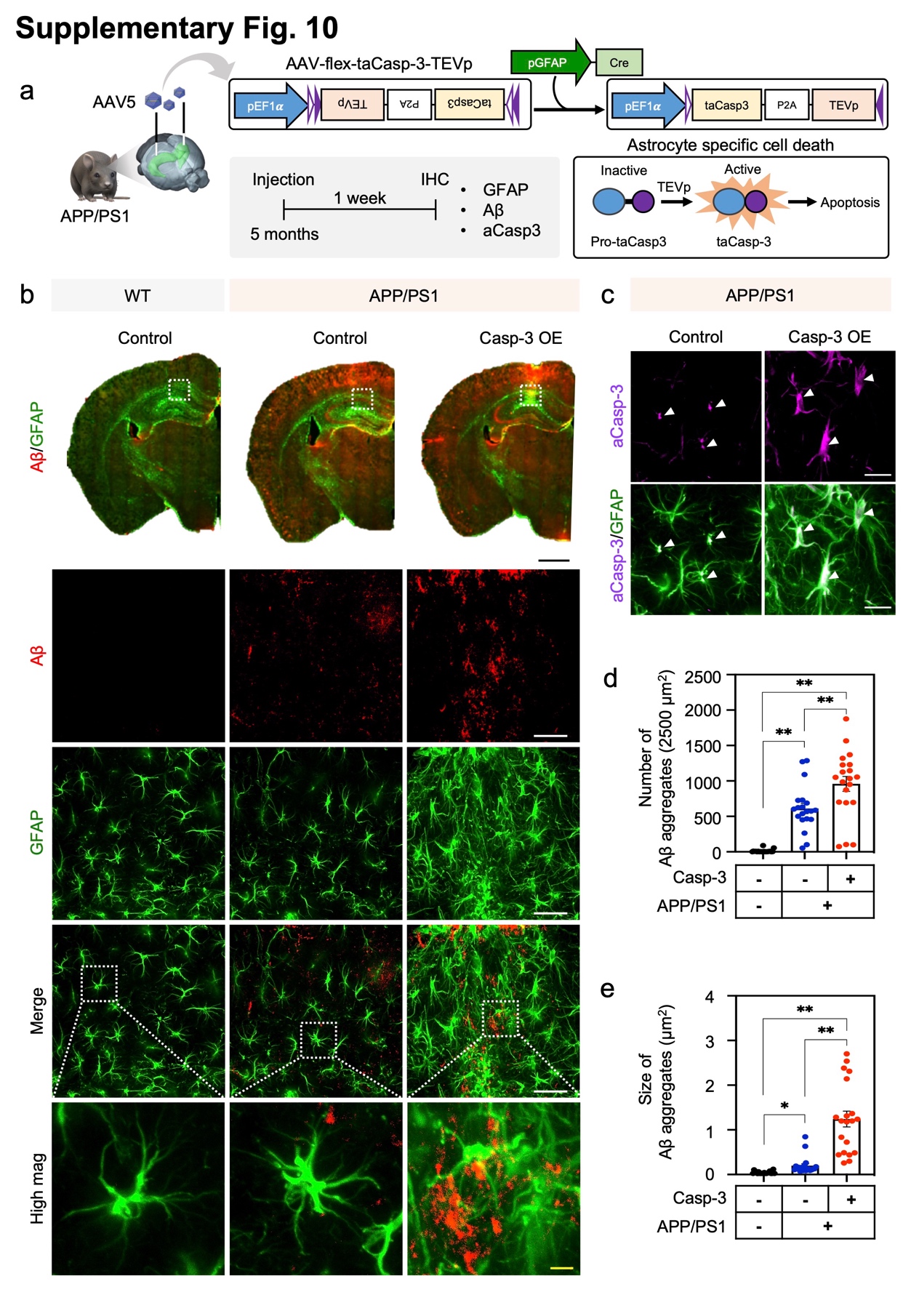


**Supplementary Fig. 10 Astrocyte-specific cell death via Cre-dependent active caspase-3 (taCasp3-2A-TEVp) expression increases Aβ aggregates and GFAP-positive astrocytes in the hippocampus of APP/PS1 mice. a,** Experimental design for inducing astrocyte-specific apoptosis via Cre-dependent active caspase-3 (taCasp3-2A-TEVp) in the hippocampus of WT and APP/PS1 mice. **b,** Representative images of immunofluorescence staining for GFAP-positive astrocytes and Aβ aggregates in three groups of mice: Group 1, WT mice with AAV-pGFAP-Cre; Group 2, APP/PS1 mice with AAV-pGFAP-Cre; Group 3, APP/PS1 mice with AAV-flex-taCasp3-2A-TEVp + AAV-pGFAP-Cre. Scale bars: black, 1mm; white (top 3 rows), 50 μm; white (bottom) 10 μm. **c,** Representative images of immunofluorescence staining for GFAP-positive astrocytes and aCasp3 in AAV-virus injected mice. Scale bar: 10 μm (white). **d,** Quantification of the number of Aβ aggregates in three groups of mice as descried in panel b. A total of 20 ROIs (2500 μm^2^) measurements from N = 4 mice in each group. **e,** Quantification of the size of Aβ aggregate in three groups of mice as descried in panel b. A total of 20 ROIs (2500 μm^2^) measurements from N = 4 mice in each group. Data are presented as mean ± SEM. Significantly different at *, p<0.05; **, p<0.01.

**
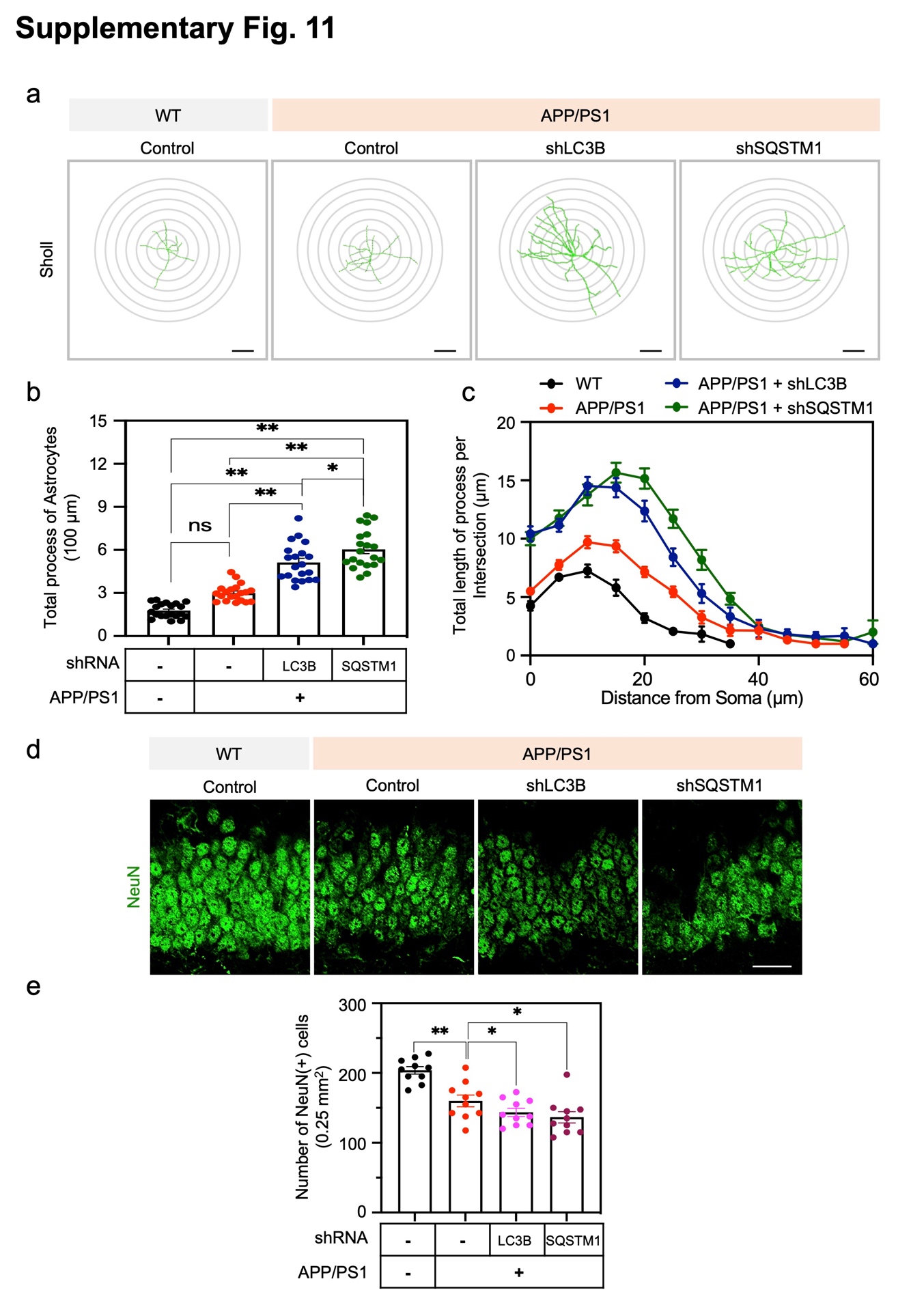
**

**Supplementary Fig. 11 Inhibition of astrocytic autophagy increases process of GFAP-positive astrocytes and exacerbates neuronal damage in APP/PS1 mice.**  **a,** Representative images of Sholl analysis for GFAP-positive astrocytes in four groups of mice: Group 1, WT mice with AAV-EF1a-DIO-Control shRNA + AAV-pGFAP-Cre; Group 2, APP/PS1 mice with AAV- EF1a-DIO- Control shRNA + AAV-pGFAP-Cre; Group 3, APP/PS1 mice with AAV-EF1a-DIO-LC3 shRNA + AAV-pGFAP-Cre; Group 4, APP/PS1 mice with AAV-EF1a-DIO-SQSTM1 shRNA + AAV-pGFAP-Cre. Scale bars (black): 10 μm. A total of 20 cell counts from N = 5 mice in each group. **b & c,** Quantification of total processes of astrocytes (**b**), and total length of processes per intersection (**c**) via Sholl analysis. A total of 20 cell counts measurements from N = 4 mice in each group. **d,** Immunofluorescence staining for NeuN in four groups of mice as described in panel a. Scale bar (white): 20 μm. **e,** Quantification of the number of NeuN-positive neurons in four groups of mice. A total of 10 ROIs (0.25mm^2^) measurements, N = 4 mice in each group. Data are presented as mean ± SEM. Significantly different at *, p<0.05; **, p<0.01.


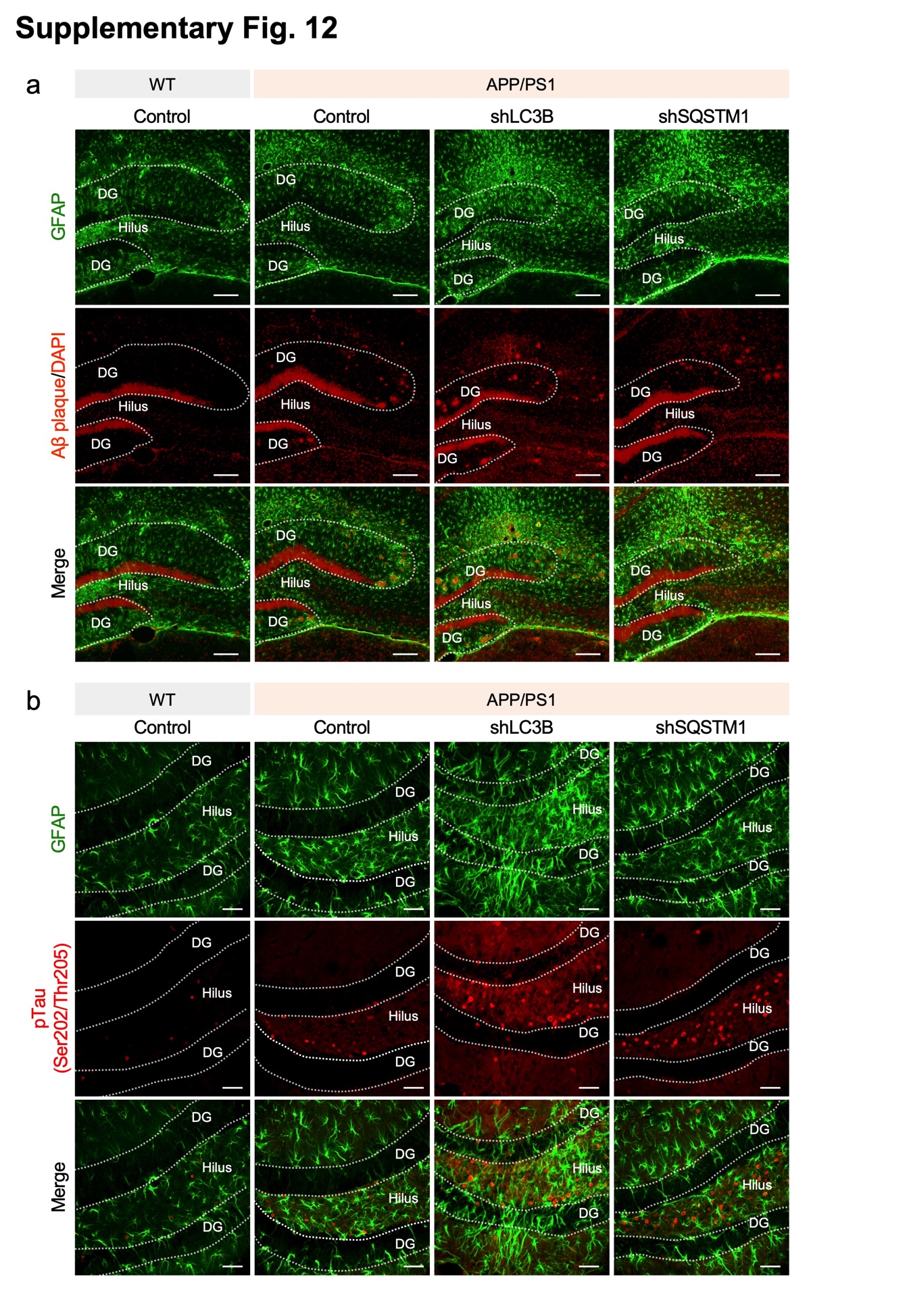


**Supplementary Fig. 12 Inhibition of astrocytic autophagy increases GFAP-positive astrocytes and exacerbates Aβ plaque formation in APP/PS1 mice.**  **a,** Representative images of immunofluorescence staining for GFAP-positive astrocytes and Aβ aggregates in four groups of mice: Group 1, WT mice with AAV-EF1a-DIO-Control shRNA + AAV-pGFAP-Cre; Group 2, APP/PS1 mice with AAV-EF1a-DIO-Control shRNA + AAV-pGFAP-Cre; Group 3, APP/PS1 mice with AAV-EF1a-DIO-LC3B shRNA + AAV-pGFAP-Cre; Group 4, APP/PS1 mice with AAV-EF1a-DIO-SQSTM1 shRNA + AAV-pGFAP-Cre. Scale bars (white): 100 μm. **b,** Representative images of immunofluorescence staining for GFAP-positive astrocytes and pTau (Ser202/Thr205) in four groups of mice as described in panel a. Scale bars (white): 50 μm.


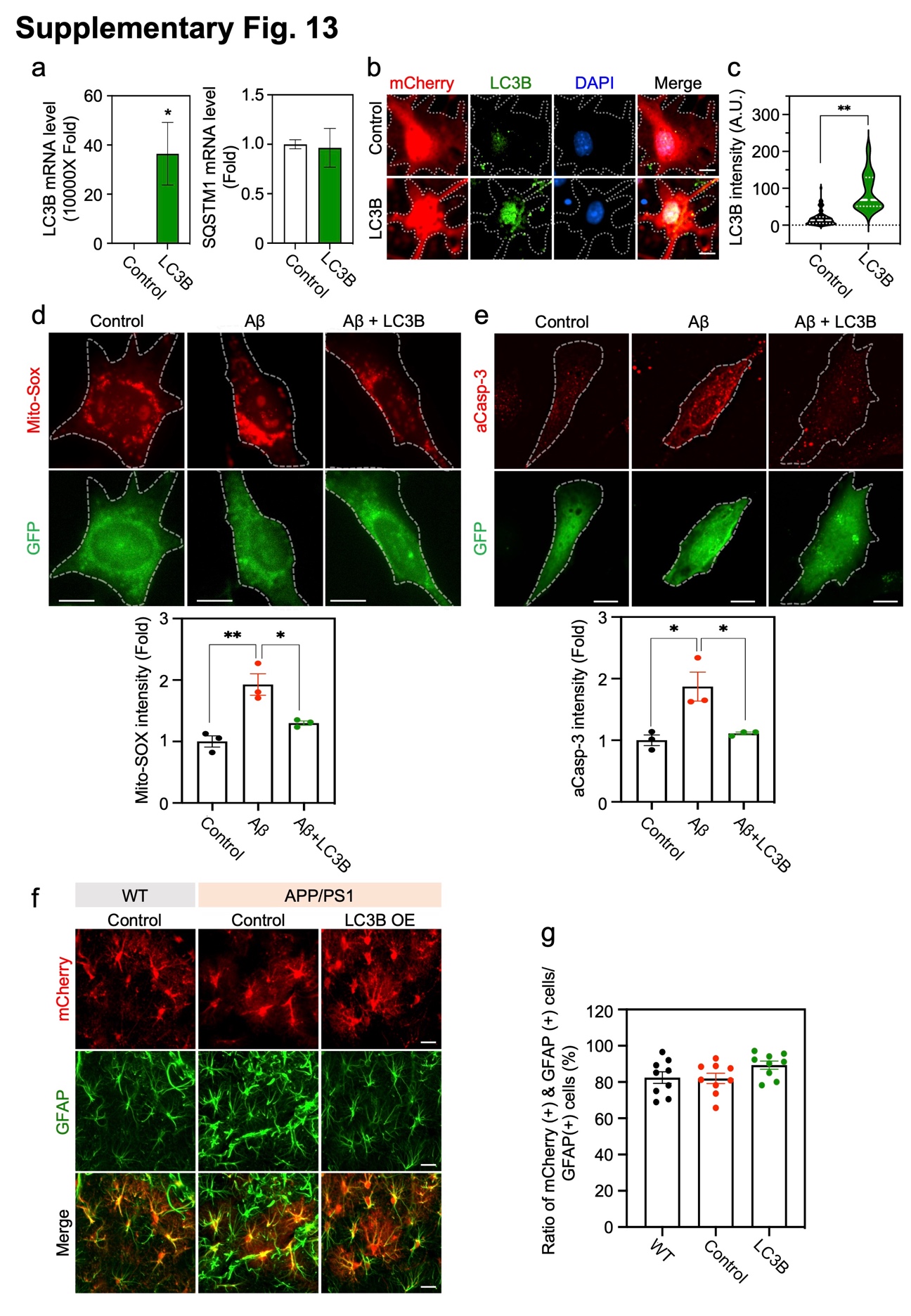


**Supplementary Fig. 13 Verification of LC3B overexpression in both astrocyte culture *in vitro* and mice *in vivo*. a,** Astrocyte-specific overexpression of LC3B by AAV-GFAP-*MAPT1LC3B(LC3B*) in primary astrocytes. Astrocyte-specific overexpression of LC3B overexpression (OE) by AAV-GFAP-*LC3B* increases LC3B mRNA level but not SQSTM1 mRNA. **b,** Representative images of astrocyte-specific overexpression of LC3B by AAV-GFAP-*LC3B* in astrocytes *in vitro*. Scale bars (white): 10 μm. **c,** Immunoreactivity of LC3B protein is significantly increased by infection of AAV-GFAP-*LC3B*. A total of 100 cell counts, 25 cells/well, *n* = 4 well. **d,** Representative images and quantification of Mito-SOX in LC3B overexpressing human astrocytes with Aβ treatment for 24hr. Representative images of Mito-SOX (red), GFP (green) (c) in LC3B overexpressed human astrocytes. Scale bars (white): 10 μm***.*** A total of 30 cell counts, 10 cells/well, *n* = 3 well. **e,** Representative images and quantification of active caspase-3 staining in LC3B overexpressing human astrocytes with Aβ treatment for 24hr. Representative images of active caspase-3 (red), GFP (green) (c) in LC3B overexpressed human astrocytes. Scale bars (white): 10 μm***.*** A total of 30 cell counts, 10 cells/well, *n* = 3 well. **f,** Representative images of astrocyte-specific overexpression of Control and *LC3B* in astrocytes of WT, APP/PS1 Control, and APP/PS1 + LC3B mice. Scale bars (white): 20μm. **g,** Immunoreactivity of control-mCherry and LC3B-mCherry are found at the similar level between three group of mice: WT, APP/PS1 Control, and APP/PS1+LC3B OE. A total of 9 ROI (0.25mm^2^) measurements from *N* = 3 mice in each group. Data are presented as mean ± SEM. Significantly different at *, *p*<0.05; **, *p*<0.01.
